# Targeting Carbohydrate Mimetics of Tetrahydrofuran-Containing Acetogenins to Prostate Cancer

**DOI:** 10.3390/molecules28072884

**Published:** 2023-03-23

**Authors:** Patricia Gonzalez Periche, Jacky Lin, Naga V. S. D. K. Bhupathiraju, Teja Kalidindi, Delissa S. Johnson, Nagavarakishore Pillarsetty, David R. Mootoo

**Affiliations:** 1 Department of Chemistry, Hunter College and The Graduate Center, City University of New York, New York, NY 10065, USA; 2 Department of Radiology, Memorial Sloan Kettering Cancer Center, New York, NY 10065, USA

**Keywords:** acetogenin, mannose, mimetic, PSMA, selectivity, prostate cancer, PC-3, LNCaP

## Abstract

The high potency of the tetrahydrofuran-containing acetogenins (THF-ACGs) against a broad range of human cancer cell lines has stimulated interest in structurally simpler mimetics. In this context, we have previously reported THF-ACG mimetics in which the THF and butenolide moieties of a mono-THF-ACG were replaced with carbohydrate and thiophene residues, respectively. In the present study, towards the targeting of these carbohydrate analogues to prostate cancer (PCa), we synthesized prodrugs in which a parent thiophene or butenolide congener was conjugated through a self-immolative linker to 2-[3-(1,3-dicarboxypropyl)ureido] pentanedioic acid (DUPA), a highly specific ligand for prostate-specific membrane antigen (PSMA), which is overexpressed on prostate tumors. Both prodrugs were found to be more active against receptor positive LNCaP than receptor-negative PC-3 cells, with 2.5 and 12 times greater selectivity for the more potent thiophene analog and the less active butenolide congener, respectively. This selectivity for LNCaP over PC-3 contrasted with the behavior of the parent drugs, which showed similar or significantly higher activity for PC-3 compared to LNCaP. These data support the notion that higher activity of these DUPA-derived prodrugs against LNCaP cells is connected to their binding to PSMA and suggest that the conjugation of PSMA ligands to this family of cytotoxic agents may be effective for targeting them to PCa.

## 1. Introduction

**Figure 1:**
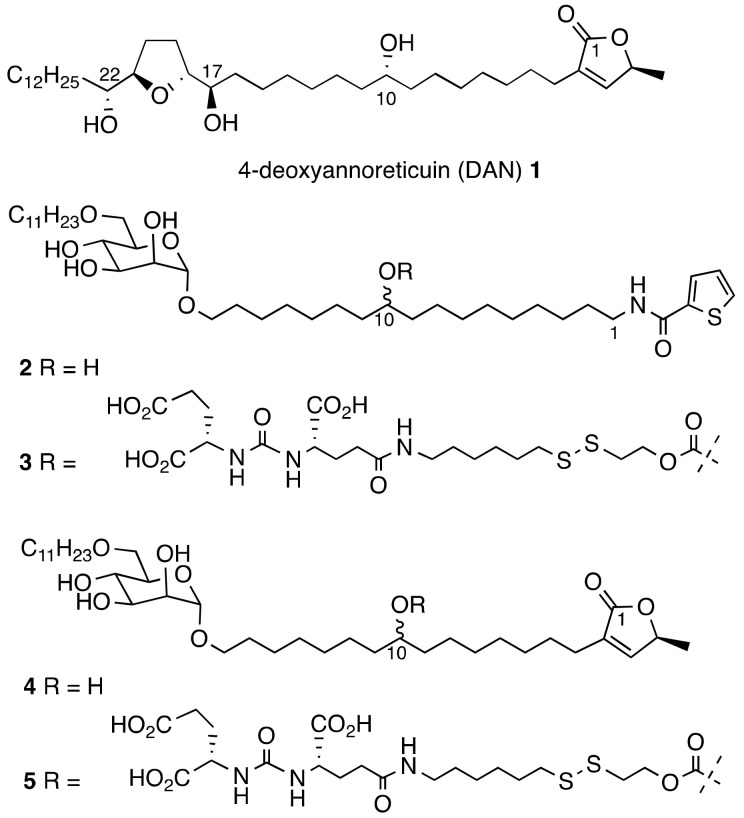
Natural and unnatural analogues of 4-DAN.

Natural product-inspired small molecules remain a primary line of cancer therapeutics. In this context, tetrahydrofuran-containing acetogenins (THF-ACGs) have attracted attention for their high potencies against a broad range of human cancer cell lines [1,2,3,4]. Their unusual mechanism of action, which may in part be due to mitochondrial ATP disruption, may make them particularly effective against tumors that are significantly dependent on oxidative phosphorylation [5,6,7,8]. Accordingly, a variety of THF ACG analogues have been examined, with a major focus being on structures in which more complex segments of naturally occurring compounds have been replaced with simpler or easily accessed substitutes [3]. In this vein, we recently reported carbohydrate analogues of naturally occurring ACG 4-deoxyannomontacin (4-DAN) **1**, wherein the THF and butenolide moieties were replaced with mannose and thiophene residues, respectively (Figure 1) [9]. These analogues **2** and **4** were prepared and screened as a mixture of C-10 epimers, as earlier studies on similar carbohydrate congeners suggested that the configuration at this central carbinol position should not impact on cytotoxicity, and for simplification of the synthesis [10]. Similar to 4-DAN, these mimetics showed cytotoxicity in the low-to-submicromolar range against two human prostate cancer (PCa) cell lines, PC-3 and LNCaP. Although their mode of action remains to be determined, **2** and **4** are attractive therapeutic leads given the prevalence of PCa [11,12], their relatively simple syntheses, and the possibility that their sugar motifs may confer favorable tumor selectivity and solubility properties.

Against this backdrop, we considered targeting PCa with prodrugs of **2** and **4** in which a tumor vector is conjugated via a self-immolative linker to the C-10 carbinol [13,14,15]. For this preliminary study, we employed an established methodology using 2-[3-(1,3-dicarboxypropyl)ureido] pentanedioic acid (DUPA) as a vector for the prostate-specific membrane antigen (PSMA) and a known disulfide-based linker. PSMA is a transmembrane glycoprotein that is overexpressed on a variety of primary and metastatic PCa and is a target for cancer diagnostics and therapeutics [16,17,18]. DUPA binds PSMA with high specificity and affinity and the receptor promotes endocytosis of bound ligands [19]. DUPA-linked conjugates of imaging and cytotoxic agents have shown significantly increased selectivity for PSMA-expressing tumors [20,21,22,23]. Drug release from prodrugs **3** and **5** is facilitated by interaction with thiols, which exist in higher concentration in cells than in plasma, thereby reducing the possibility of nonspecific uptake of the parent drugs **2** and **4,** respectively [16,17,18,19,20,21,22,23,24]. Thus, the guiding hypothesis is that the DUPA ligand would elicit selective uptake of the prodrugs in PSMA-positive cells over PSMA-negative cells, thereby leading to increased activity against the former. Herein, we describe the synthesis and initial cytotoxicity data for **3** and **5**.

## 2. Results and Discussion

### 2.1. Synthesis

**Scheme 1:**
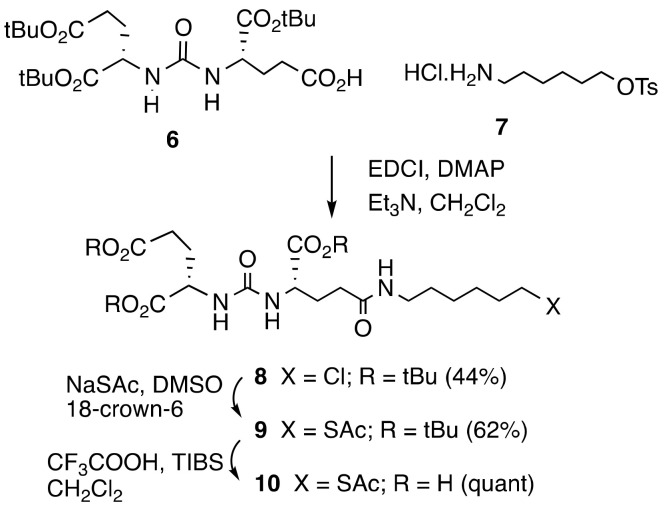
Synthesis of DUPA segment.

The synthesis of the DUPA thiol segment started with 1-ethyl-3-(3-dimethylamino-propyl) carbodiimide (EDCI)-promoted coupling of the known DUPA carboxylic acid **6** and the HCl salt of amino tosylate **7** [20,21,22,23,25] (Scheme 1). This reaction delivered the chloride **8**, which presumably resulted from the substitution of tosylate with in situ chloride. Treatment of **8** with potassium thioacetate yielded **9**. Exposure of **9** to trifluoroacetic acid in the presence of triisopropylbutylsilane (TIBS) provided **10** [26].

**Scheme 2:**
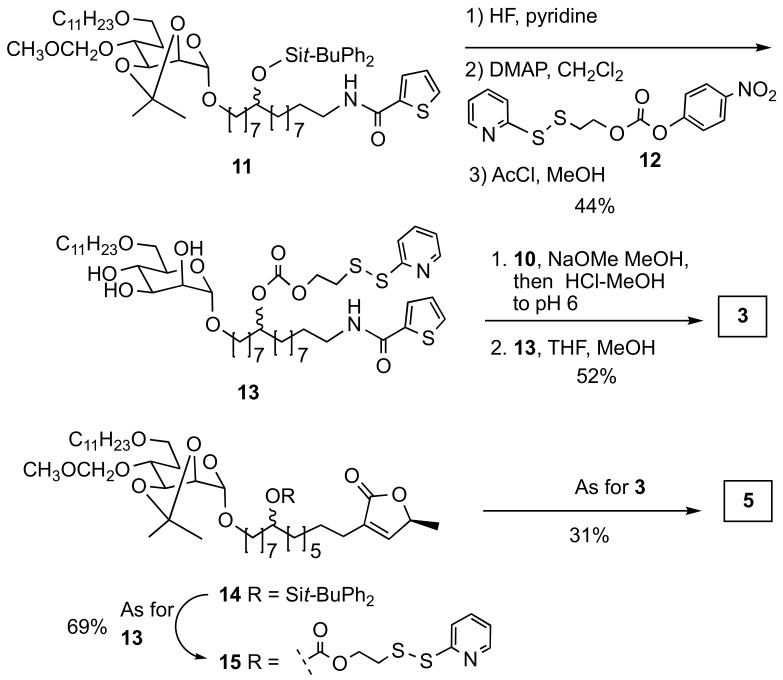
Synthesis of drug-DUPA conjugates.

For prodrug **3**, the reaction of **11**, an intermediate in the earlier synthesis of **2**, and **12**, the *p*-nitrophenyl carbonate of the 2-pyrido disulfide of 2-mercaptoethanol, followed by the removal of the acetal-protecting groups in the product, provided **13** [9,27,28] (Scheme 2). Finally, disulfide exchange on **13** with the in situ-generated thiol derivative of **10** delivered the prodrug conjugate **3**. A similar sequence of reactions on **14**, the precursor to **4**, led to prodrug **5** [9]. Prodrugs **3** and **5** were characterized by NMR and HRMS (Supplementary Material).

### 2.2. Cytotoxicity Data

The cytotoxicity of **2**–**5** against the two human prostate tumor cell lines PC-3 and LNCaP was determined by the XTT cell viability assay after 48 h of treatment with the test compounds (Table 1 and Supplementary Material). LNCaP is known to overexpress the PSMA receptor, whereas PC-3 is receptor-negative [29]. The thiophene parent drug **2** showed low-micromolar–nanomolar-range activity, with eight times higher activity against PC-3 compared to LNCaP. The butenolide drug **4** was at least an order of magnitude less potent compared to **2**, with very similar activity against both cell lines. In contrast, both prodrugs **3** and **5** were more selective for LNCaP than PC-3, albeit at lower potency compared to their respective drugs **2** and **4**. Prodrug **3**, the derivative of the thiophene drug **2** that was more selective for PC-3 over LNCaP cells, showed a reversal in selectivity, with approximately 2.6 times selectivity for LNCaP. Interestingly, the prodrug **5**, the derivative of the less potent butenolide drug **4** that was similarly active against both cell lines, was 12 times more selective for LNCaP over PC-3. In comparison, doxorubicin, which is clinically used against PCa, was found to be 3.2 times more active against LNCaP cells [30].

That both prodrugs **3** and **5** are more selective against LNCaP cells compared to their parent drugs **2** and **4,** respectively, which are more or similarly active against PC-3 cells, supports our hypothesis that the selectivity of these prodrugs is promoted by their binding to—and internalization by—PSMA on LNCaP cells. More detailed studies, for example, using cotreatment of cells with prodrug and a competing PSMA ligand, will be needed to confirm whether DUPA recognition is the basis for selectivity. Investigations with stable drug-DUPA conjugates that are not degradable intracellularly, as well as quantification of intracellular drugs and prodrug concentrations, will also be needed to interrogate whether the active drug entity is in fact the original drug. In the latter context, it could be argued that the activity of these prodrugs is primarily due to the DUPA moiety and not the original drug. However, this seems unlikely, given the fact that both **3** and **5** have the identical DUPA residue but are appreciably different in potency.

It is also noteworthy that the LNCaP selectivity observed for prodrug **5** is appreciably higher than for prodrug **3**. This higher selectivity may be connected to the lower potency of **4**, the parent drug in **5**, compared to the drug entity in **3**. A contributing factor may be the fact that **4** shows no selectivity for either cell line, whereas **2**, the parent drug in **3,** appears to be intrinsically selective for PC-3 compared to LNCaP cells. In the context of increasing selectivity of this class of DUPA conjugates to PSMA-expressing cells, known DUPA linkers that decrease overall prodrug hydrophobicity and increase PSMA affinity are envisaged [20,21,22]. Potency can be improved by increasing the activity of the drug entity, which may be possible by modifications in the lipid segments, as observed for the parent class of THF ACGs and analogues thereof [1,2,3,31,32].

## 3. Materials and Methods

Unless otherwise stated, all reactions were carried out under a nitrogen atmosphere in oven-dried glassware using standard syringe and septa technique. NMR spectra were obtained on 500 or 600 MHz instruments. Chemical shifts are relative to the deuterated solvent peak or the tetramethylsilane (TMS) peak at (δ 0.00) and are in parts per million (ppm). Assignments for selected nuclei were determined from ^1^H COSY and HSQC experiments. Thin-layer chromatography (TLC) was performed on 0.25 mm thick precoated silica gel or C18-W silica, HF254 aluminum sheets. Chromatograms were observed under UV (short- and long-wavelength) light, and/or were visualized by heating plates that were dipped in a solution of ammonium (VI) molybdate tetrahydrate (12.5 g) and cerium (IV) sulfate tetrahydrate (5.0 g) in 10% aqueous sulfuric acid (500 mL). Flash column chromatography (FCC) was performed using silica gel 60 A (230–400 mesh) or C18 (Carbon 17%) 60 A (230–400 mesh) and employed a stepwise solvent polarity gradient, correlated with TLC mobility. Hexanes used for FCC had a boiling point in the 40–60 °C range.

### 3.1. Chemical Synthesis

#### 3.1.1. 6-aminohexyl 4-methylbenzenesulfonate Hydrochloride (**7**)

6-((*tert*-butoxycarbonyl)amino)hexyl 4-methylbenzenesulfonate (5.0 g, 0.013 mol) was stirred in a solution of 4M HCl in dioxane at rt for 16 h. At that time, the mixture was concentrated in vacuo. The residue was triturated with ethyl acetate to provide **7** (4.13 g, quantitative) as a white solid. ^1^H NMR (C_5_H_5_N, 500 MHz) δ 8.01 (d, *J* = 8.1 Hz, 2H), 7.33 (d, *J* = 8.1 Hz, 2H), 4.07 (t, *J* = 6.4 Hz, 2H), 3.18 (t, *J* = 7.5 Hz, 2H), 2.24 (s, 3H), 1.90 (m, 2H), 1.50 (m, 2H), 1.23 (m, 2H), 1.17 (m, 2H); ^13^C NMR (C_5_H_5_N, 125 MHz) δ 145.6, 134.2, 129.5, 128.8, 71.5, 40.0, 29.3, 28.4, 26.7, 25.5, 21.8. HRMS (ESI) calcd for C_13_H_22_ClNO_3_SNa (M + Na)^+^ 330.0901 found 330.0898.

#### 3.1.2. Di-tert-butyl(((S)-1-(tert-butoxy)-5-((6-chlorohexyl)amino)-1,5-dioxopentan-2-yl)carbamoyl)-L-glutamate (**8**)

To a mixture of **6** (0.70 g, 1.4 mmol) and CH_2_Cl_2_ (70 mL) at 0 °C under N_2_, **7** (0.64 g, 2.1 mmol), EDCI (0.81 g, 4.24 mmol), Et_3_N (0.73 mL, 5.72 mmol), and DMAP (5 mg, 0.043 mmol) were added. The reaction mixture was then stirred at rt for 16 h, then washed successively with 1M HCl, saturated aqueous NaHCO_3_, and brine. The organic extract was dried (Na_2_SO_4_) and evaporated in vacuo. FCC of the residue afforded **8** (0.38 g, 44%) as an oil. *Rf* = 0.30 (30% EtOAc/hexanes). ^1^H NMR (CDCl_3_, 500 MHz) δ 6.61 (br s, 1H), 5.90 (m, 1H), 5.45 (m, 1H), 4.27 (m, 1H), 4.24 (m, 1H), 3.45 (m, 2H), 3.19 (m, 2H), 2.34 (m, 1H), 2.20 (m, 3H), 1.98 (m, 2H), 1.80 (m, 2H), 1.75 (m, 2H), 1.70-1.27 (m, 33H); ^13^C NMR (CDCl_3_, 125 MHz) δ 173.5, 172.6, 172.3, 172.1, 157.3, 82.2, 81.1, 80.8, 53.7, 53.2, 45.2, 39.6, 32.6, 32.1, 31.7, 29.5, 28.5, 28.3, 28.2, 26.7, 26.3. HRMS (ESI) calcd for C_29_H_52_ClN_3_O_8_Na (M + Na)^+^ 628.3335 found 628.3343.

#### 3.1.3. Tri-tert-butyl (14S,18S)-2,11,16-trioxo-3-thia-10,15,17-triazaicosane-14,18,20-tricarboxylate (**9**)

KSAc (140 mg, 1.23 mmol) was added to a mixture of **8** (235 mg, 0.39 mmol) and 18-crown-6 ether (20 mg, 0.076 mmol) in anhydrous DMSO (2 mL). The mixture was stirred at rt for 4 h, after which the solvent was evaporated in vacuo. FCC of the residue afforded **9** (154 mg, 62%) as a white solid. *Rf* = 0.55 (50% EtOAc/hexanes). ^1^H NMR (CDCl_3_, 500 MHz) δ 6.48 (m, 1H), 5.50 (br s, 1H), 5.05 (m, 1H), 4.30 (m, 1H), 4.19 (m, 1H), 3.22 (m, 2H), 2.82 (t, *J* = 7.3 Hz, 2H), 2.40 (m, 1H), 2.29 (s, 3H), 2.25 (m, 3H), 2.03 (m, 2H), 1.84 (m, 2H), 1.58-1.25 (m, 35H); ^13^C NMR (CDCl_3_, 150 MHz) δ 196.5, 173.5, 172.6, 172.2, 172.1, 157.3, 82.2, 81.1, 80.8, 53.7, 53.2, 39.5, 32.1, 31.7, 30.9, 29.5, 29.4, 29.1, 28.5, 28.4 (2 signals), 28.3 (2 signals), 28.2, 26.4. HRMS (ESI) calcd for C_31_H_55_N_3_O_9_SNa (M + Na)^+^ 668.3551 found 668.3543. 

#### 3.1.4. (14. S,18S)-2,11,16-trioxo-3-thia-10,15,17-triazaicosane-14,18,20-tricarboxylic Acid (**10**)

TFA (0.25 mL) was added to a mixture of **9** (25 mg, 0.039 mmol), TIBS (0.02 mL), and CH_2_Cl_2_ (0.25 mL) at rt. The mixture was stirred for 1.5 h, then the volatiles were removed in vacuo. FCC of the residue (25% MeOH/CH_2_Cl_2_to HOAc:MeOH: CH_2_Cl_2_, 0.1:2:8) afforded **10** (20 mg, quant.) as a white solid. *Rf* = 0.30 (30% MeOH/ CH_2_Cl_2_). ^1^H NMR (MeOD, 400 MHz) δ 4.30 (dd, *J* = 5.1, 8.0 Hz, 1H), 4.20 (dd, 5.3, 8.4 Hz, 1H), 3.21 (m, 2H), 2.88 (t, *J* = 7.2 Hz, 2H), 2.38 (m, 4H), 2.32 (s, 3H), 2.16 (m, 1H), 2.04 (m, 1H), 1.91 (m, 2H), 1.58 (m, 4H), 1.39 (m, 4H); ^13^C NMR (CDCl_3_, 100 MHz) δ 197.8, 176.8, 174.7, 160.0, 54.7, 54.0, 40.4, 31.4, 30.8, 30.6, 30.3, 29.9, 29.6, 29.5, 29.0, 27.5. HRMS (ESI) calcd for C_19_H_32_N_3_O_9_S (M + H)^+^ 478.1854 found 478.1843.

2-(pyridin-2-yldisulfaneyl)ethyl(17-(thiophene-2-carboxamido)-1-(((2*S*,3*S*,4*S*,5*S*,6*R*)-3,4,5-trihydroxy-6-((undecyloxy)methyl)tetrahydro-2*H*-pyran-2-yl)oxy)heptadecan-8-yl)carbonate (**13**)

Compound **11** [9] (200 mg, 0.19 mmol) was dissolved in pyridine (2 mL) and 30% HF/pyridine (1 mL) was added. The reaction was stirred at 45 °C for 24 h at rt for additional 24 h, then diluted with saturated aqueous NaHCO_3_ and extracted with ethyl ether. FCC of the residue afforded the desilylated alcohol derivative (91 mg, 65%). *Rf* = 0.45 (40% EtOAc/hexanes). A mixture of this material (90 mg, 0.11 mmol), **12** (80 mg, 0.23 mmol), and 4-dimethylaminopyridine (DMAP) (30 mg, 0.25 mmol) in CH_2_Cl_2_ (1 mL) was stirred at rt for 16 h. The solvent was then evaporated, and the residue subject to FCC. The partially purified product (*Rf* = 0.45 (40% EtOAc/hexanes) was dissolved in CH_2_Cl_2_ (4 mL) and 5% AcCl in MeOH (0.6 mL) was added. The mixture was stirred at rt for 30 min, then evaporated under reduced pressure at rt. FCC of the residue afforded **13** (76 mg, 44% three steps) as a pale-yellow gum. *Rf* = 0.42 (8% MeOH/CH_2_Cl_2_). ^1^H NMR (CDCl_3_, 600 MHz) δ 8.44 (m, 1H), 7.67 (dt, *J* = 1.0, 8.1 Hz, 1H), 7.62 (dt, *J* = 1.8, 7.3 Hz, 1H), 7.46 (dd, *J* = 1.1, 3.8 Hz, 1H), 7.43 (dd, *J* = 1.1, 4.5 Hz, 1H), 7.08 (ddd, *J* = 1.0, 4.8, 7.2 Hz, 1H), 7.04 (dd, *J* = 3.7, 5.0 Hz 1H), 5.99 (br s, 1H), 4.80 (d, *J* = 1.0 Hz, 1H), 4.67 (m, 1H), 4.35 (t, *J* = 6.5 Hz, 2H), 3.89 (d, *J* = 1.7 Hz, 1H), 3.82 (dd, *J* = 2.0, 8.0 Hz, 1H), 3.76 (t, *J* = 8.9 Hz, 1H), 3.72-3.62 (m, 4H), 3.48 (m, 2H), 3.38 (m, 3H), 3.05 (t, *J* = 6.5 Hz, 2H), 1.57 (m, 10H), 1.27 (m, 36H), 0.86 (t, *J* = 7.1 Hz, 3H); ^13^C NMR (CDCl_3_, 600 MHz) δ 162.1, 159.7, 155.0, 149.9, 139.3, 137.3, 129.8, 128.0, 127.7, 121.1, 120.1 (2 signals), 99.7, 79.6, 72.3, 71.9, 71.8 (2 signals), 70.7 (2 signals), 69.4 (2 signals), 68.0, 67.9, 65.2, 40.2, 37.3, 34.1 (2 signals), 32.1, 29.8 (3 signals), 29.7, 29.6, 29.5 (3 signals), 29.4 (2 signals), 27.1, 26.2 (2 signals), 25.3, 22.9, 14.3. ^1^H NMR (CD_3_OD, 600 MHz) δ 8.42 (m, 1H), 7.87 (bdd, *J* = 0.6, 8.0 Hz, 1H), 7.81 (dt, *J* = 1.8, 8.1 Hz, 1H), 7.67 (dd, *J* = 1.0, 3.7 Hz, 1H), 7.64 (bdd, *J* = 0.5, 5.0 Hz, 1H), 7.24 (dd, *J* = 5.8, 7.2 Hz, 1H), 7.13 (dd, *J* = 3.8, 4.9 Hz, 1H), 4.72 (d, *J* = 1.2 Hz, 1H), 4.70 (m, 1H), 4.37 (t, *J* = 6.1 Hz, 2H), 3.79 (m, 1H), 3.78 (dd, *J* = 1.6, 9.2 Hz, 1H), 3.73 (m, 1H), 3.68 (m, 2H), 3.62 (dd, *J* = 3.8, 10.7 Hz, 1H), 3.55 (t, *J* = 9.6 Hz, 1H), 3.52 (t, *J* = 6.6 Hz, 2H), 3.43 (m, 1H), 3.35 (t, *J* = 7.2 Hz, 2H), 3.12 (t, *J* = 6.1 Hz, 2H), 1.60 (m, 10H), 1.35 (m, 36H), 0.91 (t, *J* = 7.1 Hz, 3H). ^1^H NMR (CD_3_OD, 150 MHz) δ 164.5, 161.2, 156.6, 150.6, 140.6, 139.3, 131.5, 129.5, 128.9, 122.6, 121.4, 101.7, 80.3, 73.5, 72.9, 72.8, 72.3, 72.0, 69.1, 68.7, 66.2, 41.1, 38.9, 35.3, 32.3, 31.0 (2 signals), 30.8, 30.7 (2 signals), 30.6, 30.5, 28.2, 27.5, 27.4, 26.4, 23.9, 14.6. HRMS (ESI) calcd for C_47_H_79_N_2_O_10_S_3_ (M + H)^+^ 927.4890 found 927.4891.

#### 3.1.5. Mannose-thiophene-SS-DUPA (**3**)

Under a nitrogen atmosphere, **10** (31 mg, 0.06 mmol) was dissolved in MeOH (0.75 mL), and 1M NaOMe in MeOH (0.24 mL, 0.24 mmol) was added until pH 8–10. After 10 min, 5% AcCl in in MeOH (0.15 mL) was added until pH 6–7 to give a pale-brown suspension, which was added to a solution of **13** (38 mg, 0.04 mmol) in MeOH (0.5 mL) under nitrogen. The reaction mixture was stirred for 1h, then adjusted to pH 4 by addition of 5% AcCl in MeOH and evaporated at rt under reduced pressure. The residue was triturated with MeOH and the combined washings evaporated in vacuo. FCC of the residue on C18-SiO_2_ afforded unreacted **11** (15 mg). *Rf* = 0.05 (20% H_2_O/MeOH), and **3** (16 mg, 52% brsm) as a white solid: *Rf* = 0.32 (20% H_2_O/MeOH) ^1^H NMR (CD_3_OD, 600 MHz) δ 7.69 (dd, *J* = 1.1, 3.8 Hz, 1H, ArH), 7.64 (dd, *J* = 1.1, 5.0 Hz, 1H, ArH), 7.13 (dd, *J* = 3.8, 5.0 Hz, 1H, ArH), 4.74 (s, 1H, H-1′), 4.72 (m, 1H, H-10), 4.37 (m, 2H, H-a), 4.12 (dd, *J* = 6.2, 8.9 Hz, 2H, H-k, l), 3.82-3.54 (m, 7H, H-2′, 3′, 4′, 5′, 17, CH_2_-6′), 3.53 (t, *J* = 6.6 Hz, 2H, CH_2_-1″), 3.44 (m, 1H, H-17), 3.35 (t, *J* = 7.3 Hz, 2H, CH_2_-1), 3.20 (t, *J* = 6.6 Hz, 2H, H-h), 2.96 (t, *J* = 6.3 Hz, 2H, H-b), 2.75 (t, *J* = 7.3 Hz, 2H, H-c), 2.28 (m, 4H, CH_2_-i, n), 2.20-1.85 (m, 4H, CH_2_-j, m), 1.70 (m, 2H, CH_2_-d), 1.68-1.52 (m, 12H, CH_2_-2, 9, 11, 16, 2″, g), 1.49-1.25 (m, 40H), 0.92 (t, *J* = 7.1 Hz, 3H, CH_3_-11″); ^13^C NMR (CD_3_OD, 125 MHz) δ 182.1, 181.6, 180.0, 164.5, 160.0, 156.7, 140.6, 131.6, 129.5, 128.9, 101.7, 80.1, 73.5, 72.8 (2 signals), 72.3, 71.9, 69.1, 68.7, 66.7, 57.0, 56.0, 41.1, 40.5, 39.8, 35.4 (2 signals), 35.2, 33.3, 31.6, 31.0 (2 signals), 30.9, 30.8, 30.7, 30.6 (3 signals), 30.5, 30.2, 29.4, 28.2, 27.8, 27.5, 27.4, 26.4, 23.9, 14.7. HRMS (ESI) calcd for C_59_H_103_N_4_O_18_S_3_ (M + H)^+^ 1251.6424 found 1251.6420.

#### 3.1.6. 1-((S)-5-methyl-2-oxo-2,5-dihydro-1H-1λ3-furan-3-yl)-15-(((2S,3S,4S,5S,6R)-3,4,5-trihydroxy-6-((undecyloxy)methyl)tetrahydro-2H-pyran-2-yl)oxy)pentadecan-8-yl (2-(pyridin-2-yldisulfaneyl)ethyl) Carbonate (**15**)

Compound **14** [9] (245 mg, 0.25 mmol) was dissolved in pyridine (1.5 mL), and 70% HF/pyridine (1 mL) was added. The reaction was stirred at 50 °C for a further 24 h, at which time additional 70% HF/pyridine (1 mL) was introduced and stirring continued at rt for 48 h. The reaction mixture was then diluted with saturated aqueous NaHCO_3_ and extracted with ethyl ether. FCC of the residue afforded unreacted **14** (30 mg) and the desilylated alcohol derivative (138 mg, 85% based on recovered starting material). *Rf* = 0.25 (5% acetone/CH_2_Cl_2_). ^1^H NMR (CDCl_3_, 500 MHz) δ 6.96 (s, 1H), 4.99 (s, 1H), 4.97 (m, 1H), 4.86 (apparent d, *J* = 6.2 Hz, 1H), 4.66 (apparent d, *J* = 6.3 Hz, 1H), 4.17 (m, 1H), 4.07 (d, *J* = 5.6 Hz, 1H), 3.68 (m, 4H), 3.56 (m, 2H), 3.47 (m, 1H), 3.38 (m, 5H), 2.41 (bs, 1H), 2.26 (m, 2H), 1.60-1.32 (m, 48H), 0.86 (t, *J* = 6.9 Hz, 3H); ^13^C NMR (CDCl_3_, 600 MHz) δ 174.1, 149.1, 134.5, 109.4, 97.1, 95.5, 78.7, 76.2, 73.4, 72.1, 72.0, 69.9, 68.4, 67.6, 56.2, 37.7 (2 signals), 32.1, 29.9, 29.8, 29.7 (2 signals), 29.6, 29.5 (2 signals), 29.3, 28.0, 27.6, 26.6, 26.3 (2 signals), 25.8, 25.3, 22.9, 19.4, 14.3.

A mixture of the product from the previous step (115 mg, 0.16 mmol), **12** (100 mg, 0.31 mmol), and DMAP (57 mg, 0.47 mmol) in CH_2_Cl_2_ (1 mL) was stirred at rt for 72 h. The solvent was then evaporated in vacuo. FCC of the residue provided the derived carbonate (150 mg, 93%). *Rf* = 0.55 (5% acetone/ CH_2_Cl_2_). ^1^H NMR (CDCl_3_, 600 MHz) δ 8.45 (d, *J* = 4.7 Hz, 1H), 7.63 (m, 2H), 7.07 (m, 1H), 6.95 (bs, 1H), 4.98 (s, 1H), 4.96 (m, 1H), 4.85 (apparent d, *J* = 6.2 Hz, 1H), 4.64 (m, 2H), 4.35 (t, *J* = 6.1 Hz, 1H), 4.10 (m, 1H), 4.05 (m, 1H), 3.56 (m, 3H), 3.48 (m, 1H), 3.45 (m, 1H), 3.37 (m, 5H), 3.04 (t, *J* = 6.1 Hz, 2H), 2.22 (m, 2H), 1.52 (m, 10H), 1.49 (s, 3H), 1.37 (d, *J* = 6.8 Hz, 3H), 1.29 (s, 3H), 1.27-1.21 (m, 30H), 0.84 (t, *J* = 6.9 Hz, 3H); ^13^C NMR (CDCl_3_, 500 MHz) δ 174.1, 159.7, 155.0, 149.9, 149.1, 137.3, 134.4, 121.1, 120.0, 109.4, 97.1, 96.5, 79.6, 78.6, 76.2, 73.3, 72.0, 69.8, 68.3, 67.6, 65.2, 56.2, 37.2, 34.2, 32.1, 29.9, 29.8, 29.7, 29.5, 29.4, 29.3 (2 signals), 28.0, 27.5, 26.6, 26.3, 25.3, 22.9, 19.4, 14.3. 

A portion of the product from the previous step (75 mg, 0.074 mmol) was dissolved in CH_2_Cl_2_ (4 mL) and treated with 5% AcCl in MeOH (1.5 mL). The reaction was stirred for 7 h at rt, then adjusted to pH 6 with NaOMe/MeOH. The mixture was concentrated in vacuo. FCC of the residue (5% MeOH/CH_2_Cl_2_) afforded **15** (56 mg, 87%). *Rf* = 0.50 (5% MeOH/CH_2_Cl_2_). ^1^H NMR (CDCl_3_, 500 MHz) δ 8.51 (d, *J* = 4.5 Hz, 1H), 7.69 (m, 2H), 7.14 (m, 1H), 7.01 (s, 1H), 5.02 (m, 1H), 4.85 (s, 1H), 4.60 (m, 1H), 4.41 (t, *J* = 6.5 Hz, 2H), 3.94 (br s, 1H), 3.86 (dd, *J* = 3.3, 9.1 Hz, 1H), 3.82-3.45 (m, 5H), 3.54 (m, 2H), 3.43 (m, 1H), 3.10 (t, *J* = 6.5 Hz, 2H), 2.28 (t, *J* = 8.2 Hz, 2H), 1.59 (m, 12H), 1.40 (d, *J* = 6.8 Hz, 3H), 1.28 (m, 30H), 0.90 (t, *J* = 6.8 Hz, 3H); ^13^C NMR (CDCl_3_, 125 MHz) δ 174.2, 160.0, 155.0, 149.9, 137.3, 134.4, 121.1, 120.1, 99.7, 79.6, 72.3, 71.9, 71.8, 70.8, 70.6, 69.3 (2 signals), 68.0 (2 signals), 65.2, 37.2, 34.2, 32.1, 29.9, 29.8, 29.7, 29.6, 29.5 (2 signals), 29.4 (2 signals), 29.3 (2 signals), 27.5, 26.2 (3 signals), 25.3 (2 signals), 22.9, 19.4, 14.3. HRMS (ESI) calcd for C_45_H_76_NO_11_S_2_ (M + H)^+^ 870.4860 found 870.4850.

#### 3.1.7. Mannose-butenolide-SS-DUPA (**5**)

Under a nitrogen atmosphere, **10** (80 mg, 0.16 mmol) was dissolved in MeOH (0.5 mL), and 1M NaOMe in MeOH (0.65 mL, 0.65 mmol) was added until pH 10-12. After 15 min, 5% AcCl in in MeOH (0.3 mL) was added until pH 6–7 to give a pale-brown suspension, which was added to a solution of **15** (54 mg, 0.064 mmol) in MeOH (0.5 mL) and THF (0.05 mL) under nitrogen. The reaction mixture was stirred for 30 min, then adjusted to pH 4–5 by addition of 5% AcCl in MeOH and evaporated at rt under reduced pressure. The residue was triturated with MeOH and the combined washings evaporated in vacuo. FCC of the residue on C18-SiO_2_ afforded **5** (24 mg, 31%) as a white amorphous solid. *Rf* = 0.45 (20% H_2_O/MeOH). ^1^H NMR (CD_3_OD, 600 MHz) δ 7.28 (s, 1H, H, =CH)), 5.08 (m, 1H, CH_3_CH-OC=O), 4.73 (partially buried s, 1H, H-1′), 4.71 (m, 1H, H-10), 4.38 (m, 2H, H-a), 4.12 (m, 2H, H-k, l), 3.81-3.54 (m, 7H, H-2′, 3′, 4′, 5′, 17, CH_2_-6′), 3.52 (t, J = 6.6 Hz, 2H, CH_2_-1″), 3.43 (m, 1H, H-17), 3.21 (t, *J* = 6.5 Hz, 2H, H-h), 2.97 (t, *J* = 6.3 Hz, 2H, H-b), 2.76 (t, *J* = 7.3 Hz, 2H, H-c), 2.26 (m, 6H, CH_2_- 3, i, n), 2.15–1.85 (m, 4H, CH_2_-j, m), 1.75 (m, 2H, CH_2_-d), 1.65 – 1.55 (m, 12H, CH_2_-4, 9, 11, 16, 2″, g), 1.50 – 1.30 (m, 39H), 0.92 (t, *J* = 6.8 Hz, 3H, CH_3_-11″); ^13^C NMR (CD_3_OD, 125 MHz) δ 181.9, 181.4, 179.9, 176.3, 175.7, 159.9, 156.6, 152.4, 134.5, 101.6, 80.0, 79.7, 73.4, 72.7 (2 signals), 72.2, 71.8, 68.9, 68.6, 66.6, 56.8, 55.9, 40.4, 39.7, 38.2, 35.3 (2 signals), 35.1, 33.2, 31.5, 30.9, (2 signals), 30.7, 30.6 (2 signals), 30.5, 30.4, 30.3, 30.1 (2 signals), 29.3, 28.5, 27.7, 27.4, 27.3, 26.3 (2 signals), 26.0, 23.8, 19.3, 14.5. HRMS (ESI) calcd for C_57_H_100_N_3_O_19_S_2_ (M + H)^+^ 1194.6387 found 1194.6299.

### 3.2. Cytotoxicity Measurements

#### 3.2.1. Cell Lines and Cell Number Optimization

LNCaP (PSMA+, androgen-dependent) and PC-3 (PSMA-, androgen-independent) cells were purchased from the American Type Culture Collection (ATCC, Manassas, VA, USA), authenticated and routinely checked for mycoplasma contamination. LNCaP cells were cultured in Roswell Park Memorial Institute (RMPI) + 10% fetal calf serum (FCS) media and PC-3 cells were cultured in F12K + 10% FCS media. Media were supplemented with penicillin (100 I.U/mL) and streptomycin (100 µg/mL) to minimize bacterial contamination. The media were prepared by MSK Media preparation core. XTT reagent (2,3-bis-(2-methoxy-4-nitro-5-sulfophenyl)-2H-tetrazolium-5-carboxanilide) used for the study was purchased from ATCC (30–1011 K). Serial dilutions of cells in culture media were plated in triplicate from 100–100,000 in a 96-well plate. Cells were incubated at 37 °C in a CO_2_ incubator for 72 h. An activated solution of XTT reagent was made by adding 0.1 mL of activation reagent (sterile solution of N-methyl dibenzopyrazine methyl sulfate) to 5 mL of XTT reagent. 50 mL of activated XTT reagent was added per well. The plate was incubated for 2 h and absorbance from wells was measured at 475 nM. Background was measured at 660 nM. Background subtraction was made, and absorbance was plotted against cell number. The optimum cell number was determined based on the linear range of the XTT standard curve.

#### 3.2.2. IC_50_ Determination

Serial dilutions of compounds to be tested were made in culture media from 1 nM–1 mM concentration. The determined cell number from the standard graph was plated in a 96-well plate in triplicate per each dilution of the compounds. The cells were allowed to attach overnight by incubation at 37 °C in a CO_2_ incubator. The media from the well were removed and media with serial dilutions of the compounds were added to the cells. The cells were incubated with the compound for 48 h at 37 °C in CO_2_ incubator. Activated XTT reagent was added to the wells and incubated for 2 h. Absorbance from the wells was measured at 475 nM and background at 660 nM. Background subtraction was made, and all the data were plotted in prism and IC_50_ values were determined.

## 4. Conclusions

Two prodrugs comprising carbohydrate mimetics of mono-THF-containing acetogenins conjugated via a self-immolative linker to a ligand for PSMA, were prepared. These conjugates were found to be approximately 3 and 12 times more active against LNCaP, a PSMA receptor-positive prostate tumor cell line, compared to PC-3, a PSMA receptor-negative prostate tumor cell line. This result contrasted with the activity of the nonconjugated cytotoxic agents, which showed similar or higher activity against PC-3 compared to LNCaP. These data suggest that targeting PSMA may be viable strategy for modulating the tumor selectivity of this family of cytotoxic agents and new directions for carbohydrate-based therapeutics. Studies on improving prodrug selectivity and potency using modified DUPA linkers and drug entities, as well as assays against a wider panel of human tumor and normal cells are directions for future work. Mechanistic and drug development investigations, including cytotoxicity assays in the presence of competing PSMA ligands, and determination of drug uptake, stability and cellular distribution will also be performed.

## Figures and Tables

**Table 1:** Cytotoxicity (IC_50_, mM) of **2**–**5**.

Test Compound	LNCaP	PC3	Selectivity LNCaP/PC-3
**2**	0.48	0.06	0.125
**3**	0.81	2.1	2.6
**4**	3.4	4.5	1.3
**5**	4.8	58	12
Doxorubicin	0.71	2.3	3.2

## Data Availability

The data presented in this study are available in on request from the authors.

## Supplementary Materials

The following supporting information can be downloaded at: https://www.mdpi.com/article/10.3390/molecules28072884/s1, NMR spectra for **2**–**6**, **8**–**10**, **13**, **15**, cytotoxicity data for **2**–**5**.
