# Supplementary material for: Targeting Carbohydrate Mimetics of Tetrahydrofuran-Containing Acetogenins to Prostate Cancer
Source: Molecules. 2023 Mar 23;28(7):2884. doi: 10.3390/molecules28072884 (PMC10095889; doi:10.3390/molecules28072884)
Supplement: Supplementary file 1 [file molecules-28-02884-s001.zip › molecules-2283793-supplementary.pdf]

Targeting carbohydrate mimetics of the tetrahydrofuran containing acetogenins to prostate cancer

Patricia Gonzalez Periche, Jacky Lin, Naga V. S. D. K. Bhupathiraju, Teja Kalidindi, Delissa S. Johnson, Nagavarakishore Pillarsetty and David R. Mootoo*

Department of Chemistry, Hunter College, 695 Park Avenue, New York, NY 10065 and The Graduate Center, CUNY, 365 Fifth Avenue, New York, NY 10016.

SUPPORTING INFORMATION

1. NMR Spectra for **2-6**, **8-10**, **13**, **15**
2. Cytotoxicity Data for **2-5**

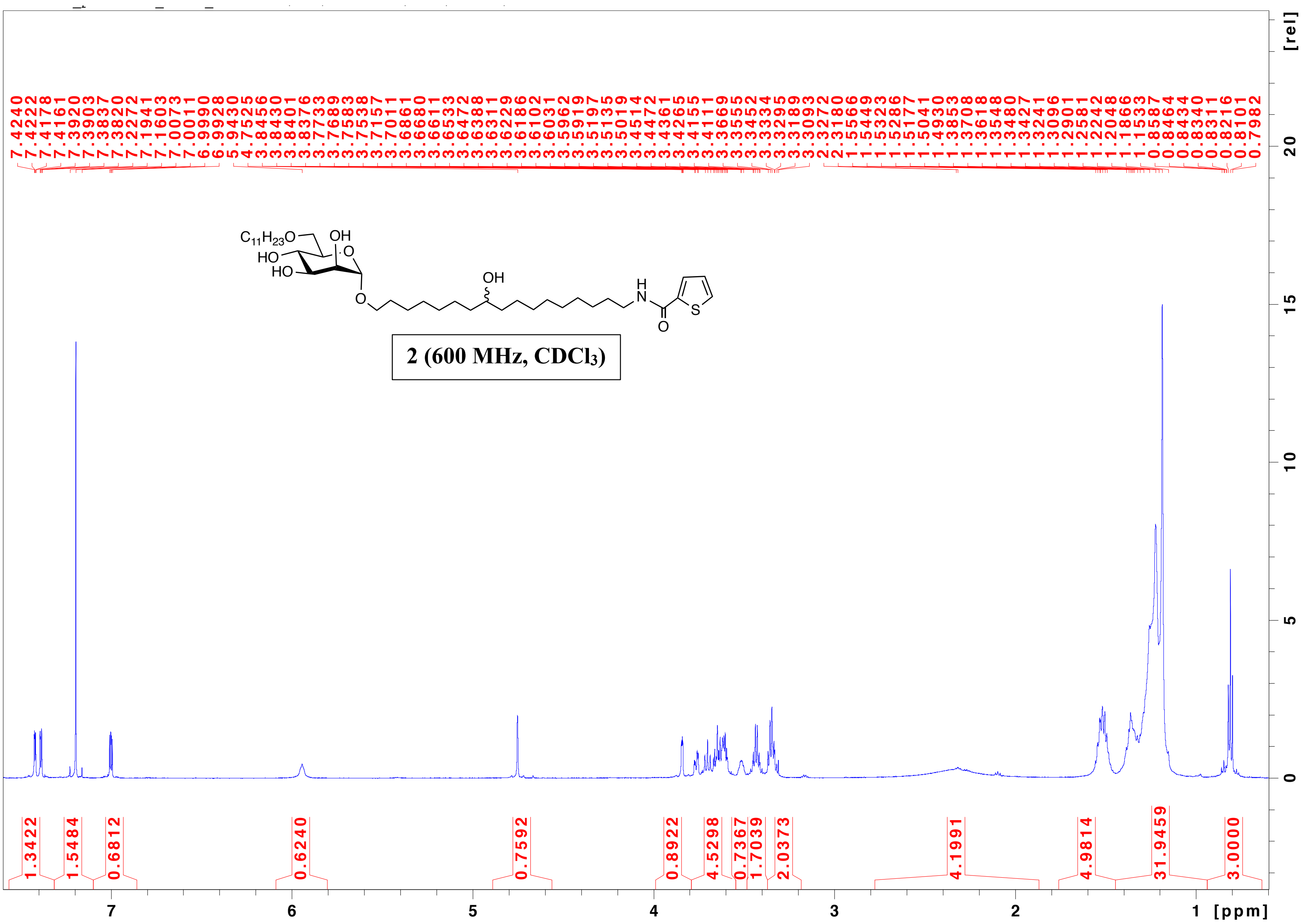

2 (600 MHz, CDCl3)
[rel]
[ppm]
1.3422
1.5484
0.6812
0.6240
0.7592
0.8922
4.5298
0.7367
1.7039
2.0373
4.1991
4.9814
31.9459
3.0000

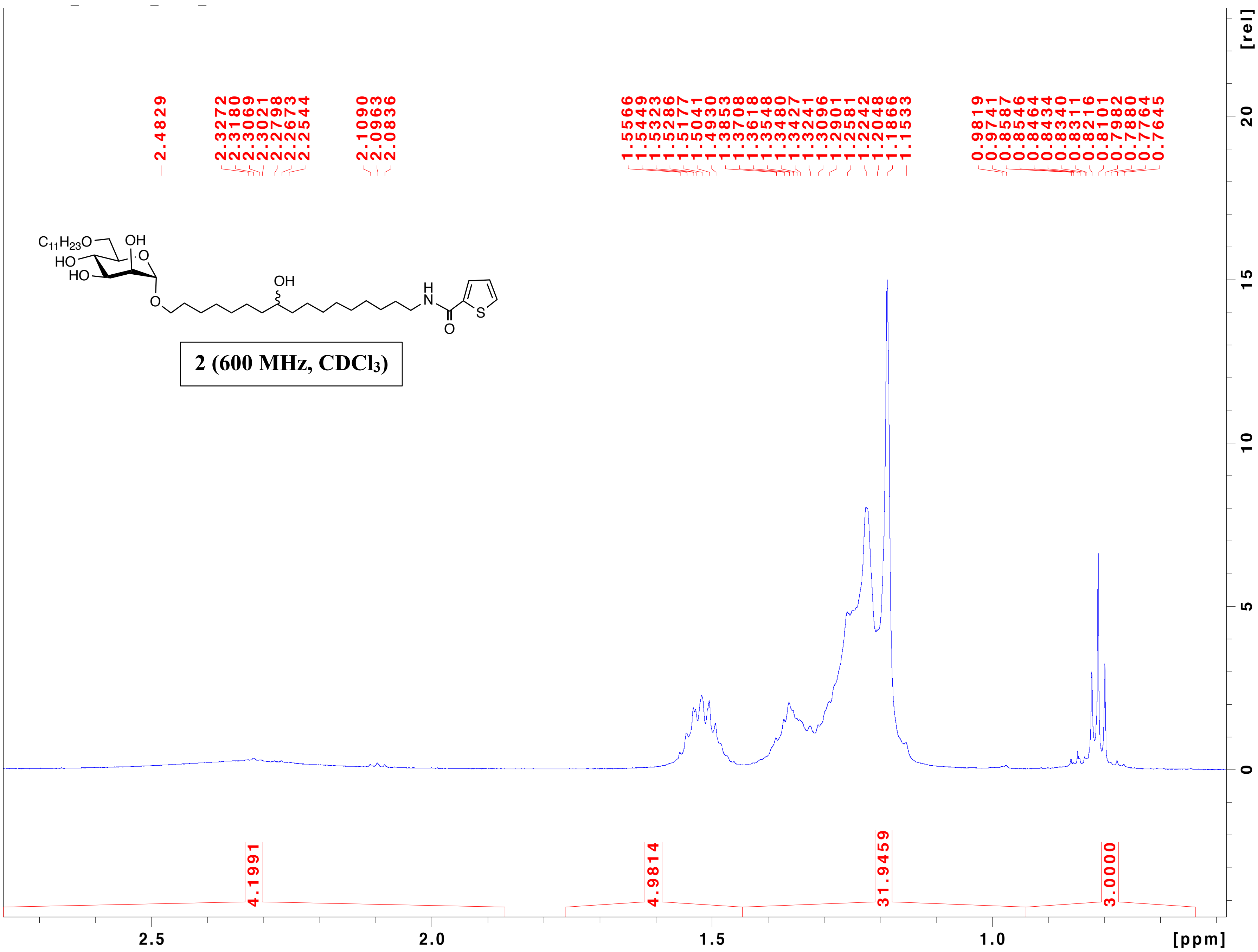

C11H23O
OH
O
HO
HO
OH
O
H
N
S
O
2 (600 MHz, CDCl3)
[rel]
[ppm]
4.1991
4.9814
31.9459
3.0000

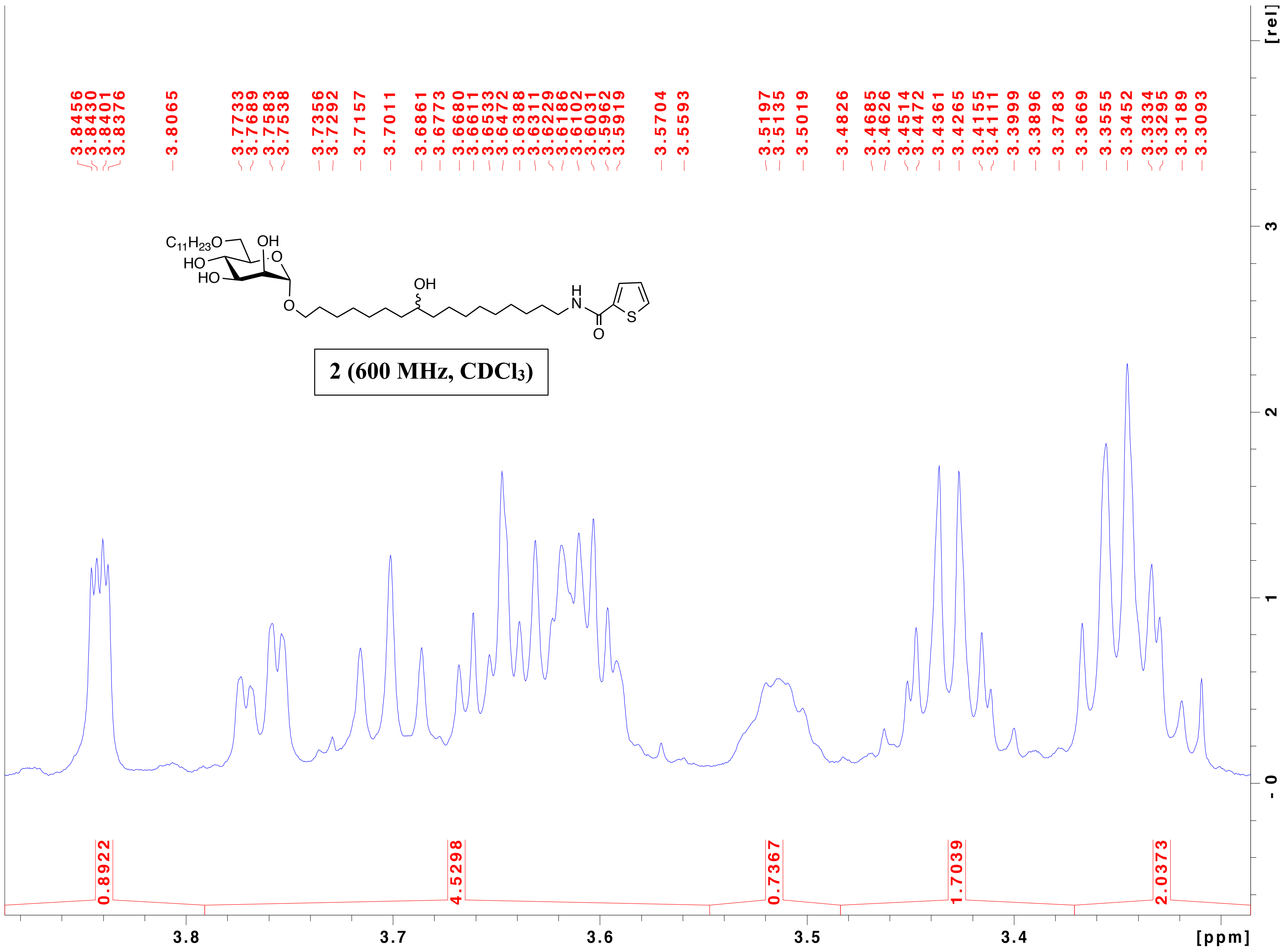

2 (600 MHz, CDCl3)

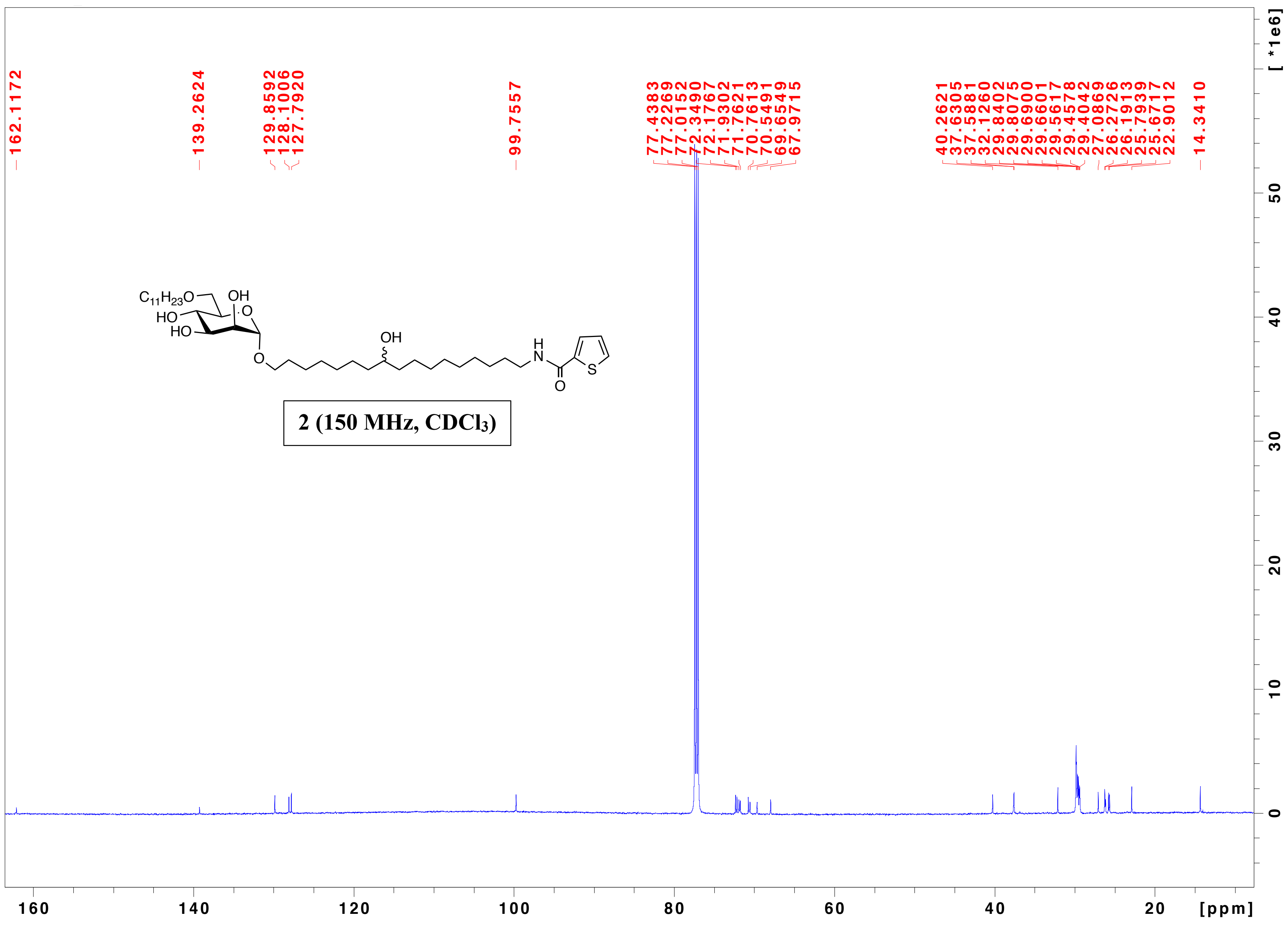
162.1172
139.2624
129.8592
128.1006
127.7920
99.7557
77.4383
77.2269
77.0152
72.3490
72.1767
71.9302
71.7621
70.7613
70.5491
69.6549
67.9715
40.2621
37.6305
37.5881
32.1260
29.8402
29.8075
29.6900
29.6601
29.5617
29.4578
29.4042
27.0869
26.2726
26.1913
25.7939
25.6717
22.9012
14.3410
[*1e6]
$C_{11}H_{23}O$
OH
O
HO
HO
O
OH
H
N
S
O
2 (150 MHz, $CDCl_3$)
160
140
120
100
80
60
40
20
[ppm]
50
40
30
20
10
0

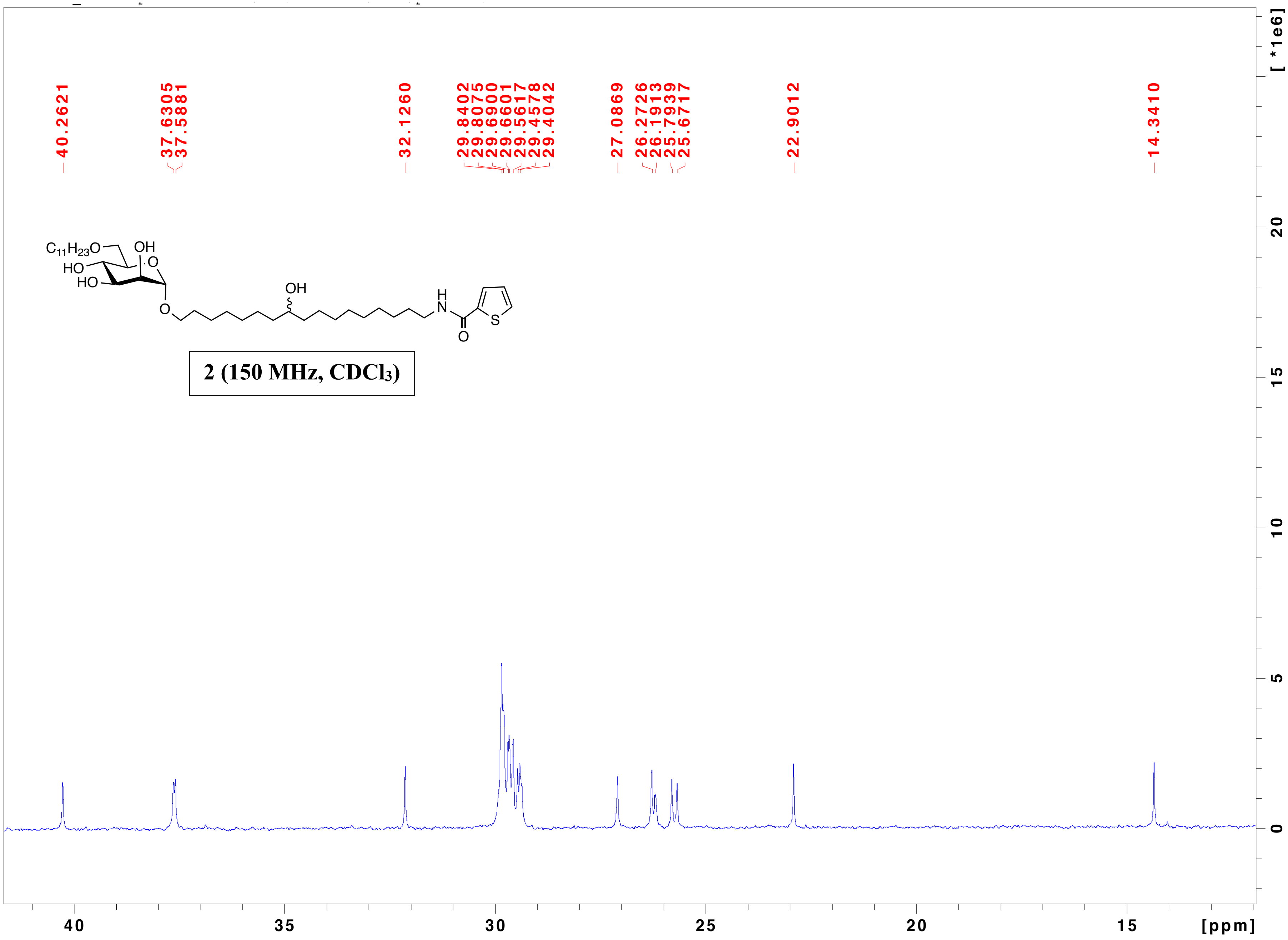

40.2621
37.6305
37.5881
32.1260
29.8402
29.8075
29.6900
29.6601
29.5617
29.4578
29.4042
27.0869
26.2726
26.1913
25.7939
25.6717
22.9012
14.3410
2 (150 MHz, CDCl3)
[*1e6]
[ppm]

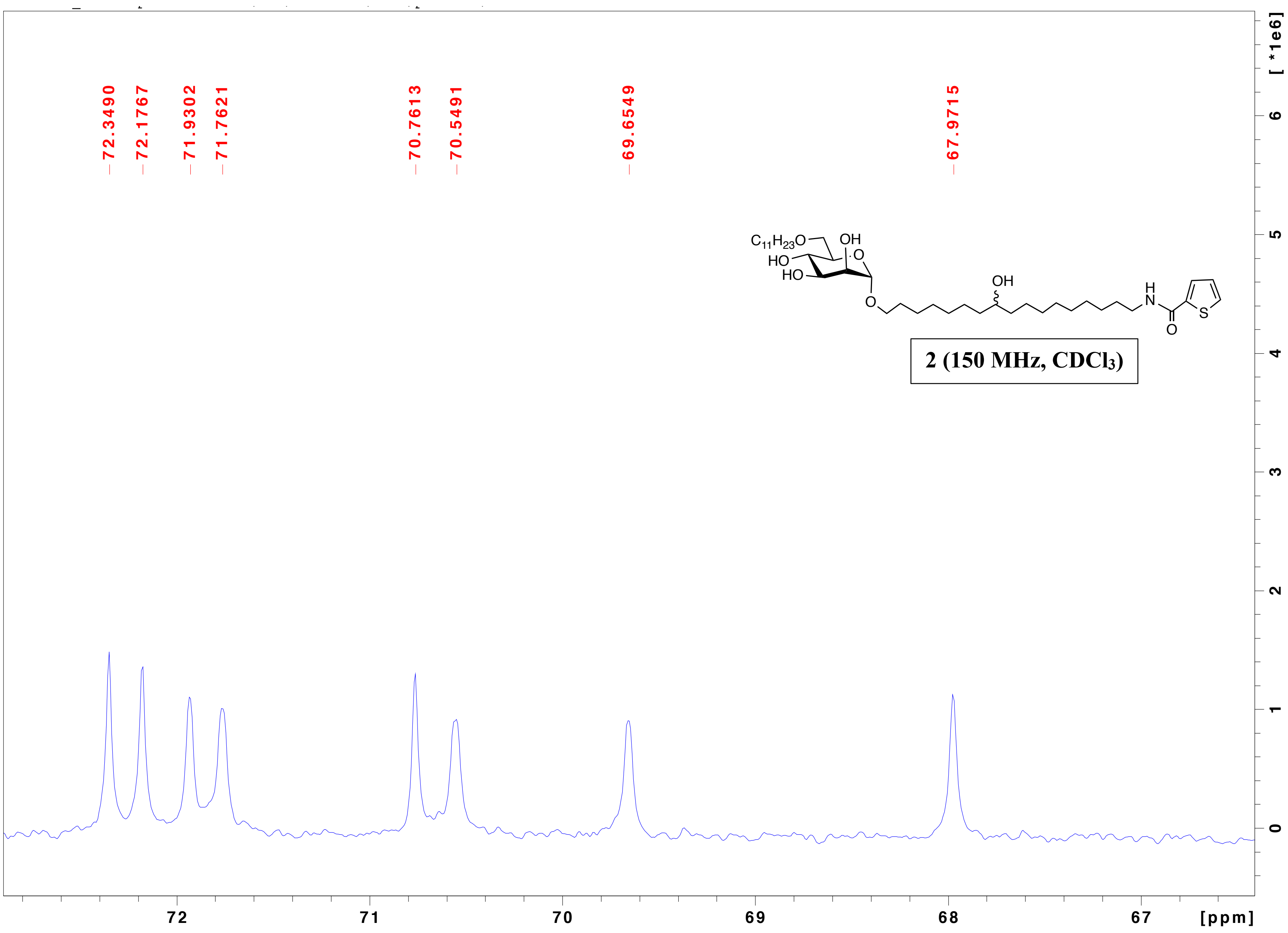

2 (150 MHz, $CDCl_3$)

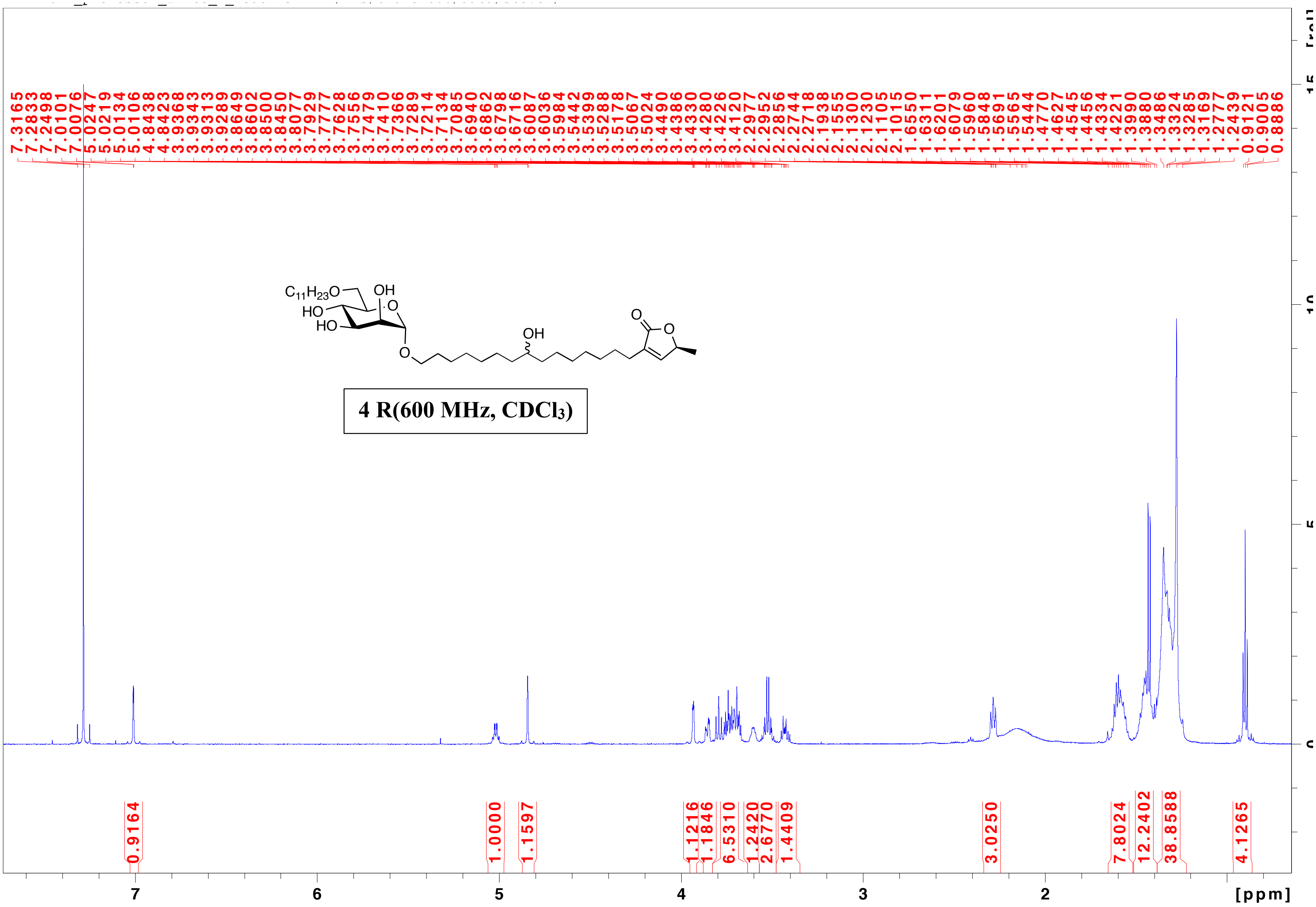

4 R(600 MHz, CDCl3)

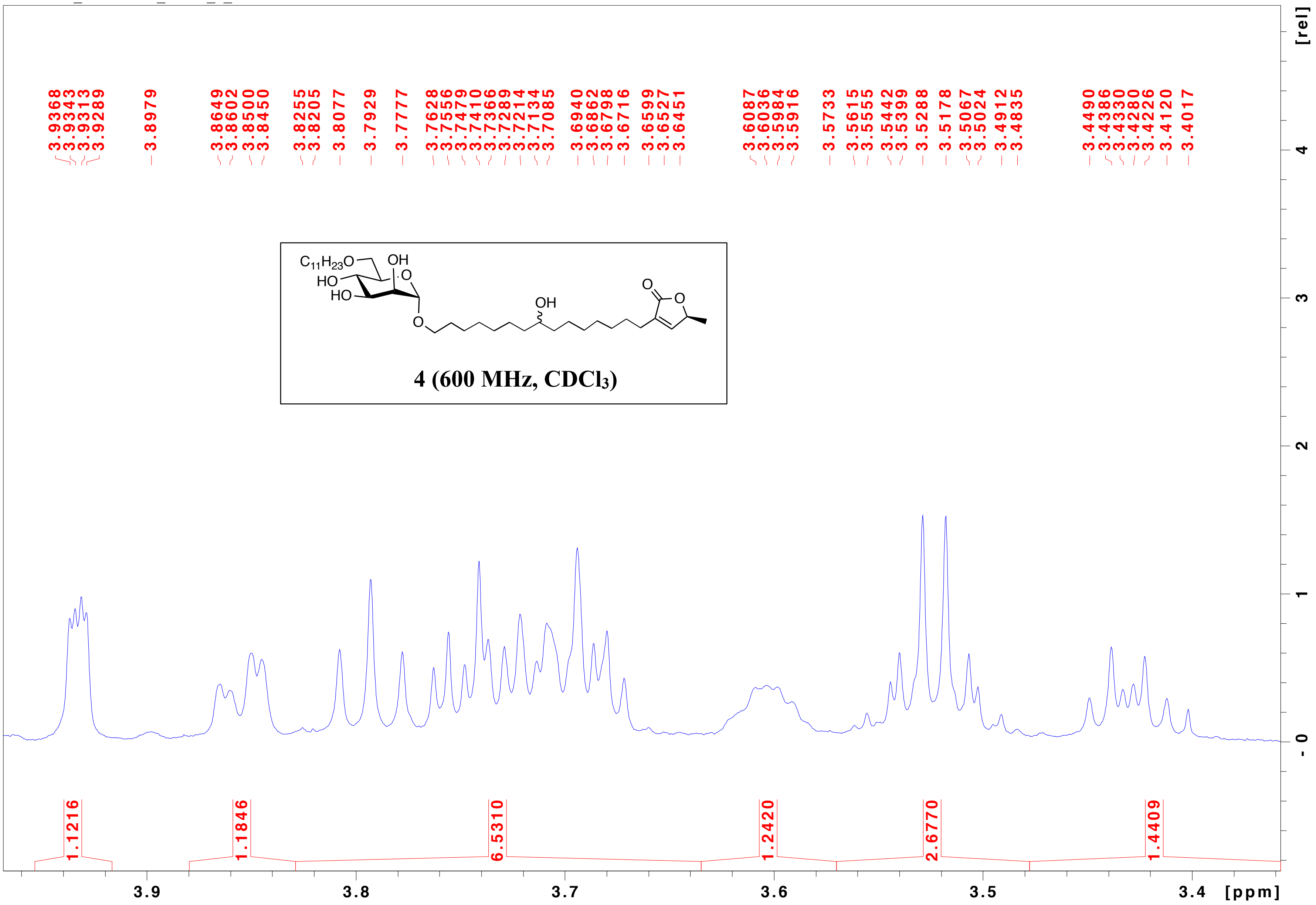
3.9368
3.9343
3.9313
3.9289
3.8979
3.8649
3.8602
3.8500
3.8450
3.8255
3.8205
3.8077
3.7929
3.7777
3.7628
3.7556
3.7479
3.7410
3.7366
3.7289
3.7214
3.7134
3.7085
3.6940
3.6862
3.6798
3.6716
3.6599
3.6527
3.6451
3.6087
3.6036
3.5984
3.5916
3.5733
3.5615
3.5555
3.5442
3.5399
3.5288
3.5178
3.5067
3.5024
3.4912
3.4835
3.4490
3.4386
3.4330
3.4280
3.4226
3.4120
3.4017
$C_{11}H_{23}O$
OH
O
HO
HO
O
OH
O
O
4 (600 MHz, $CDCl_3$)
1.1216
1.1846
6.5310
1.2420
2.6770
1.4409
3.9
3.8
3.7
3.6
3.5
3.4
[ppm]
[rel]
4
3
2
1
0

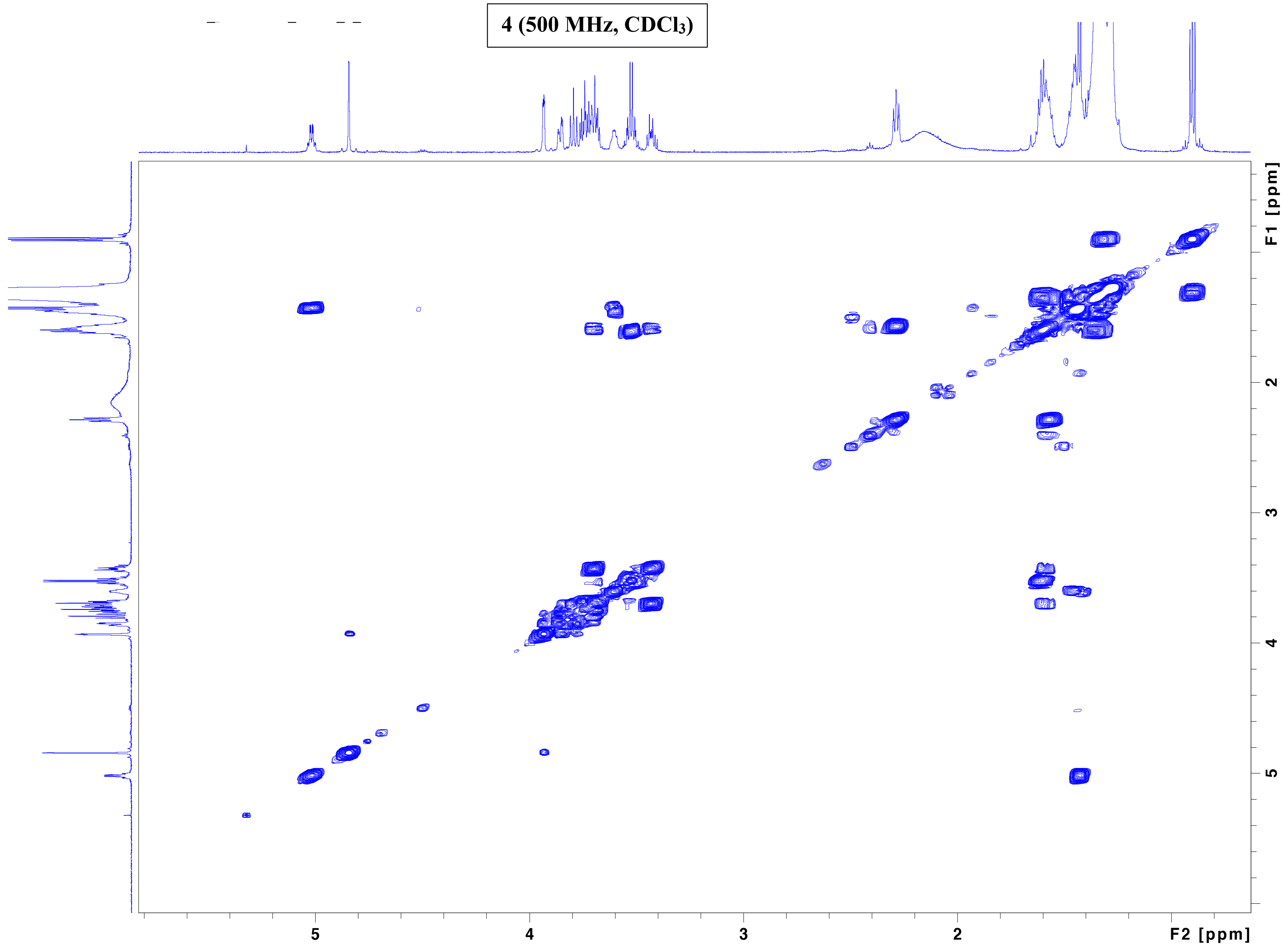
4 (500 MHz, CDCl3)
F1 [ppm]
F2 [ppm]
5
4
3
2

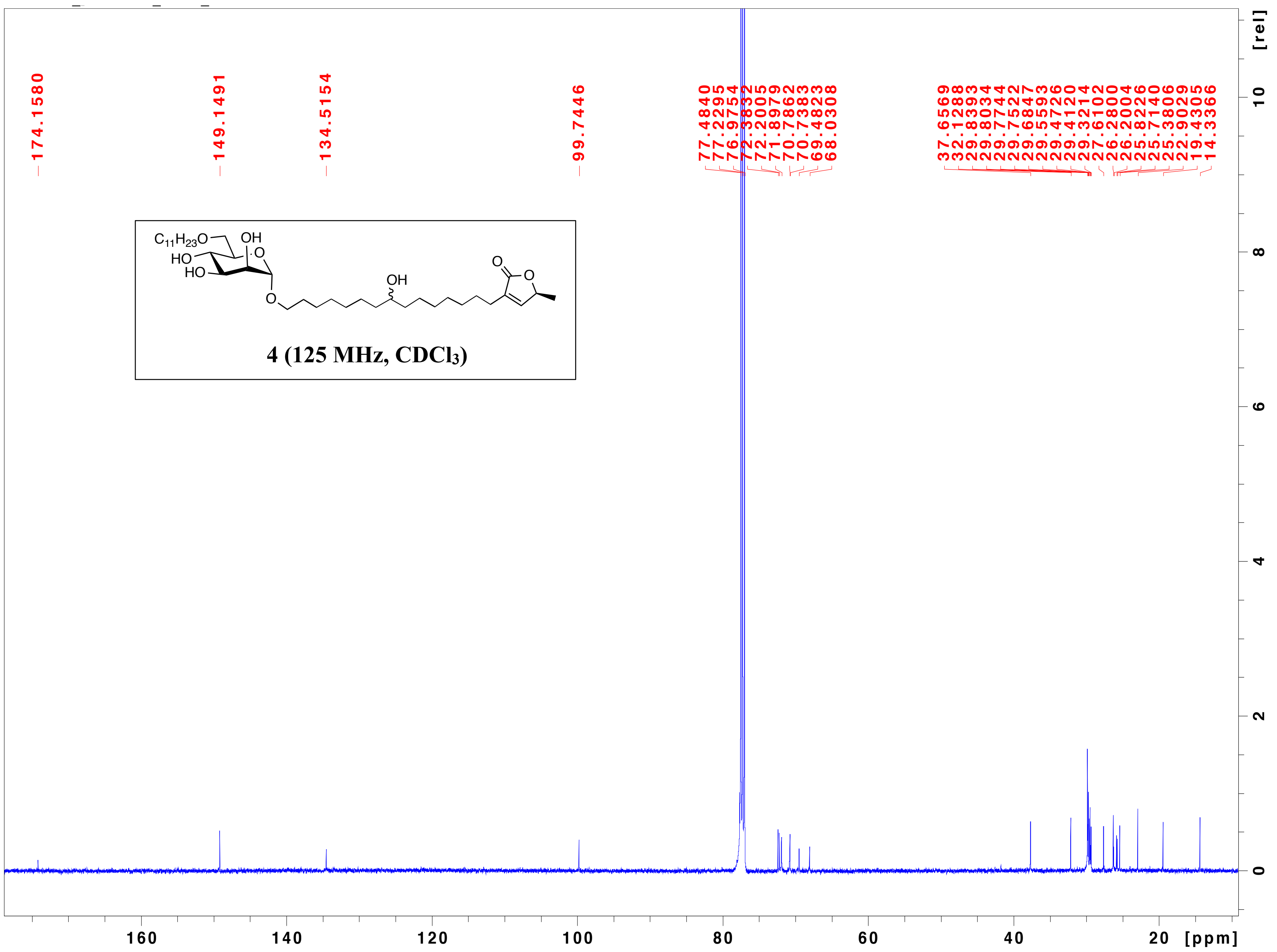

174.1580
149.1491
134.5154
99.7446
77.4840
77.2295
76.9754
72.3832
72.2005
71.8979
70.7862
70.7383
69.4823
68.0308
37.6569
32.1288
29.8393
29.8034
29.7742
29.7522
29.6847
29.5593
29.4720
29.4120
29.3214
27.6102
26.2800
26.2004
25.8226
25.7140
25.3806
22.9029
19.4305
14.3366
$C_{11}H_{23}O$
HO
HO
OH
O
O
OH
O
O
4 (125 MHz, $CDCl_3$)
[rel]
10
8
6
4
2
0
160
140
120
100
80
60
40
20
[ppm]

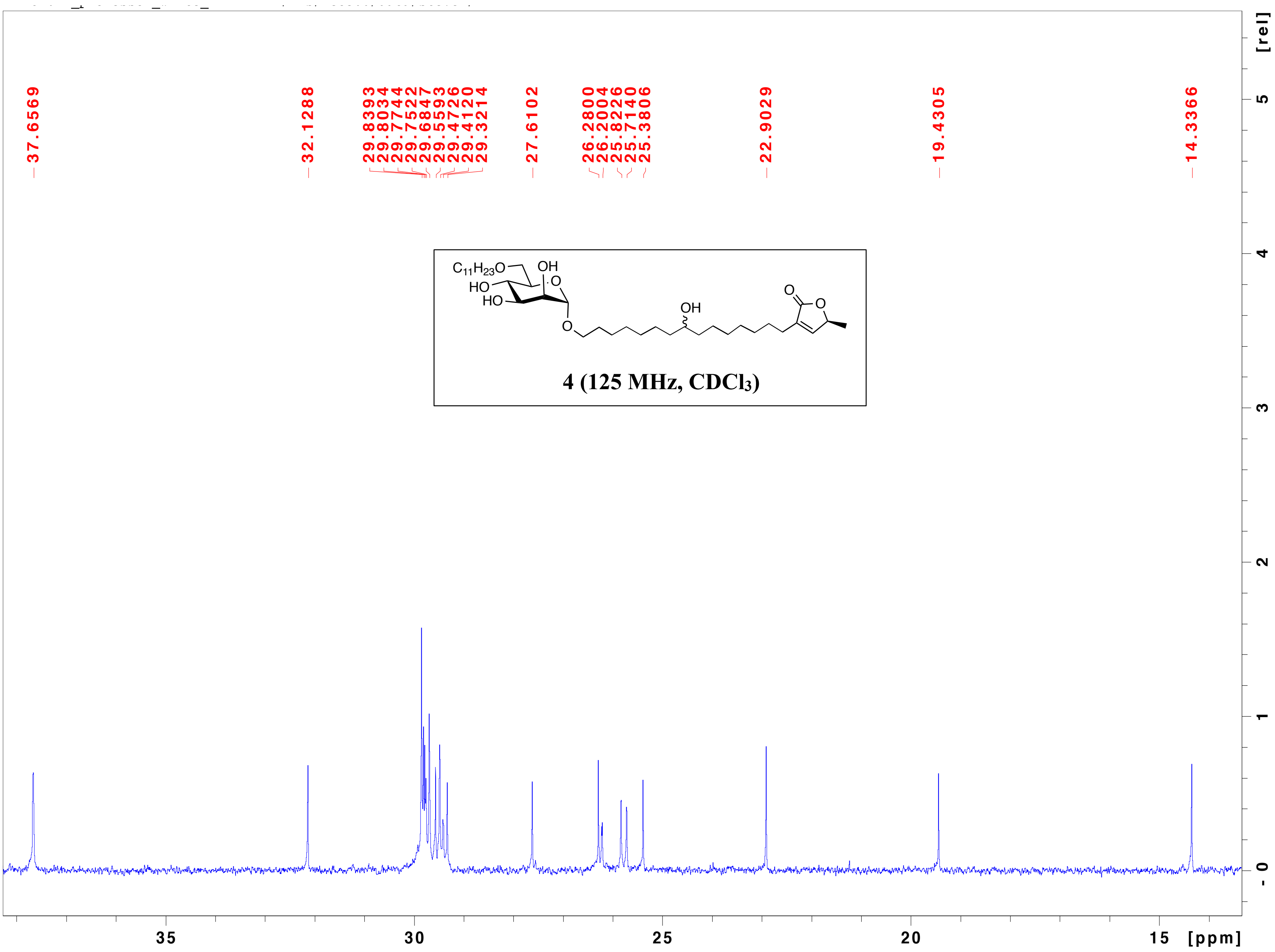
37.6569
32.1288
29.8393
29.8034
29.7744
29.7522
29.6847
29.5593
29.4726
29.4120
29.3214
27.6102
26.2800
26.2004
25.8226
25.7140
25.3806
22.9029
19.4305
14.3366
[rel]
$C_{11}H_{23}O$
OH
O
HO
HO
OH
O
O
O
4 (125 MHz, $CDCl_3$)
35
30
25
20
15
[ppm]

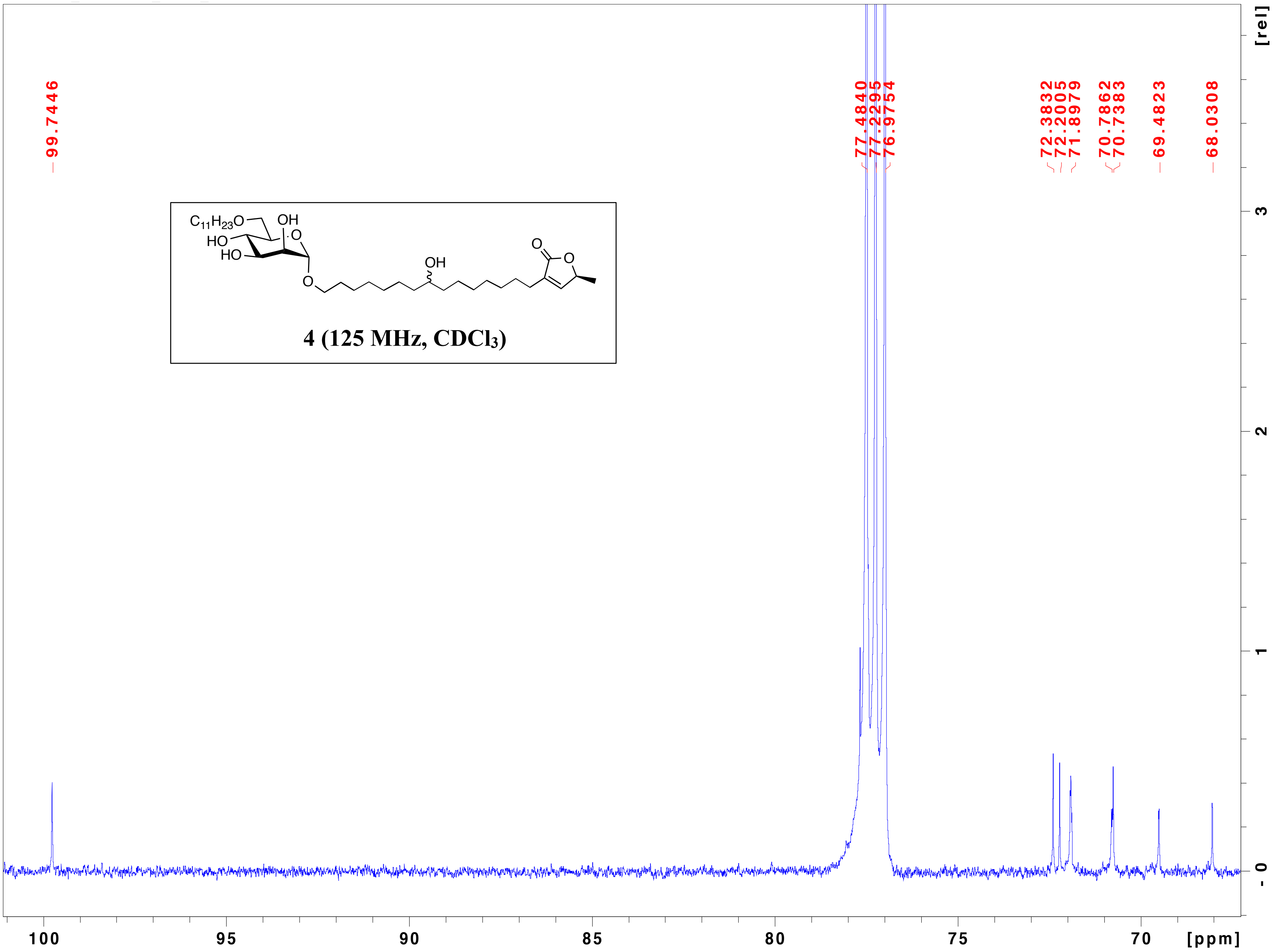
99.7446
77.4840
77.2295
76.9754
72.3832
72.2005
71.8979
70.7862
70.7383
69.4823
68.0308
4 (125 MHz, CDCl3)
[rel]
[ppm]
100
95
90
85
80
75
70
0
1
2
3

4 (125 MHz, CDCl3)

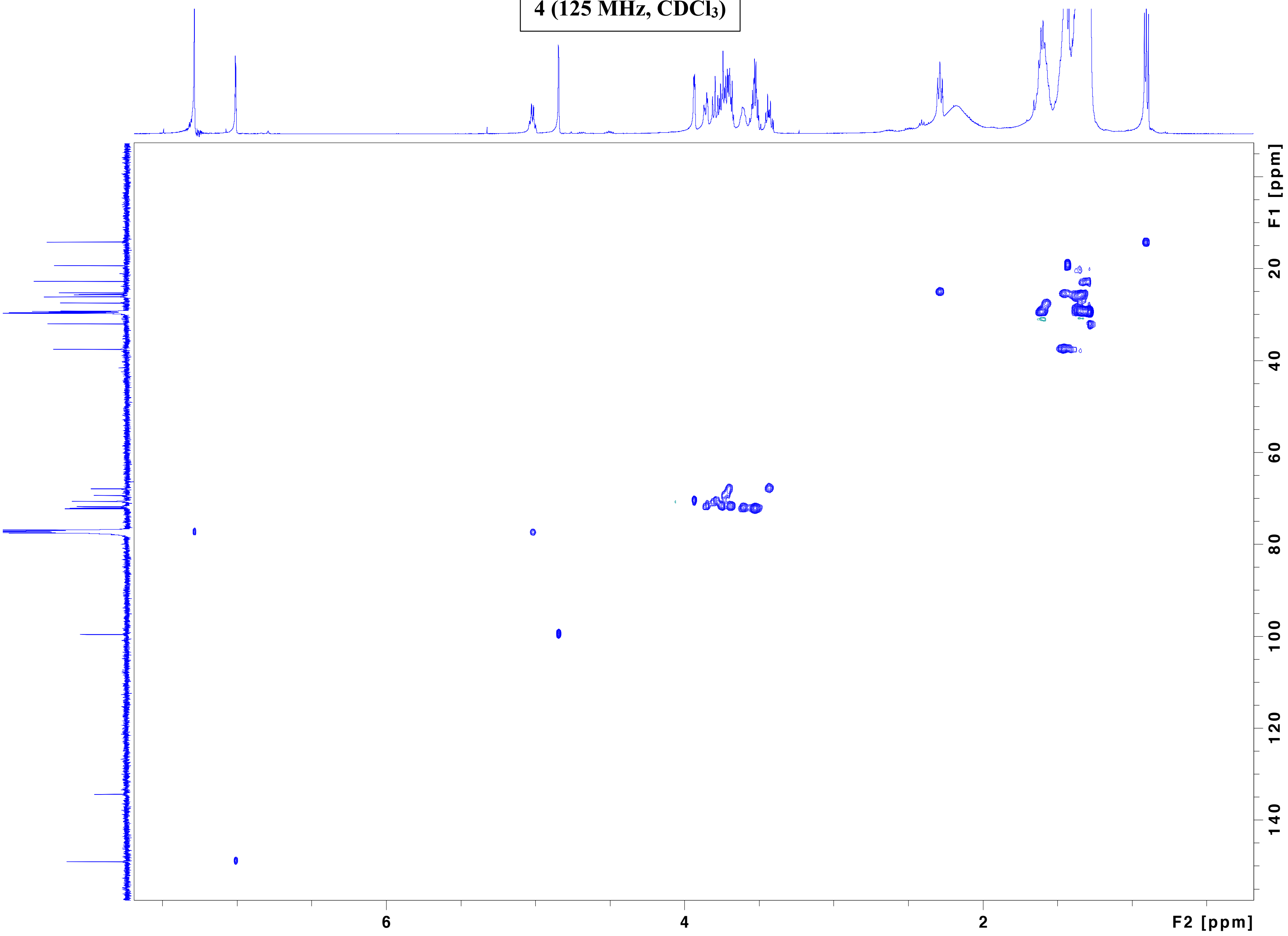

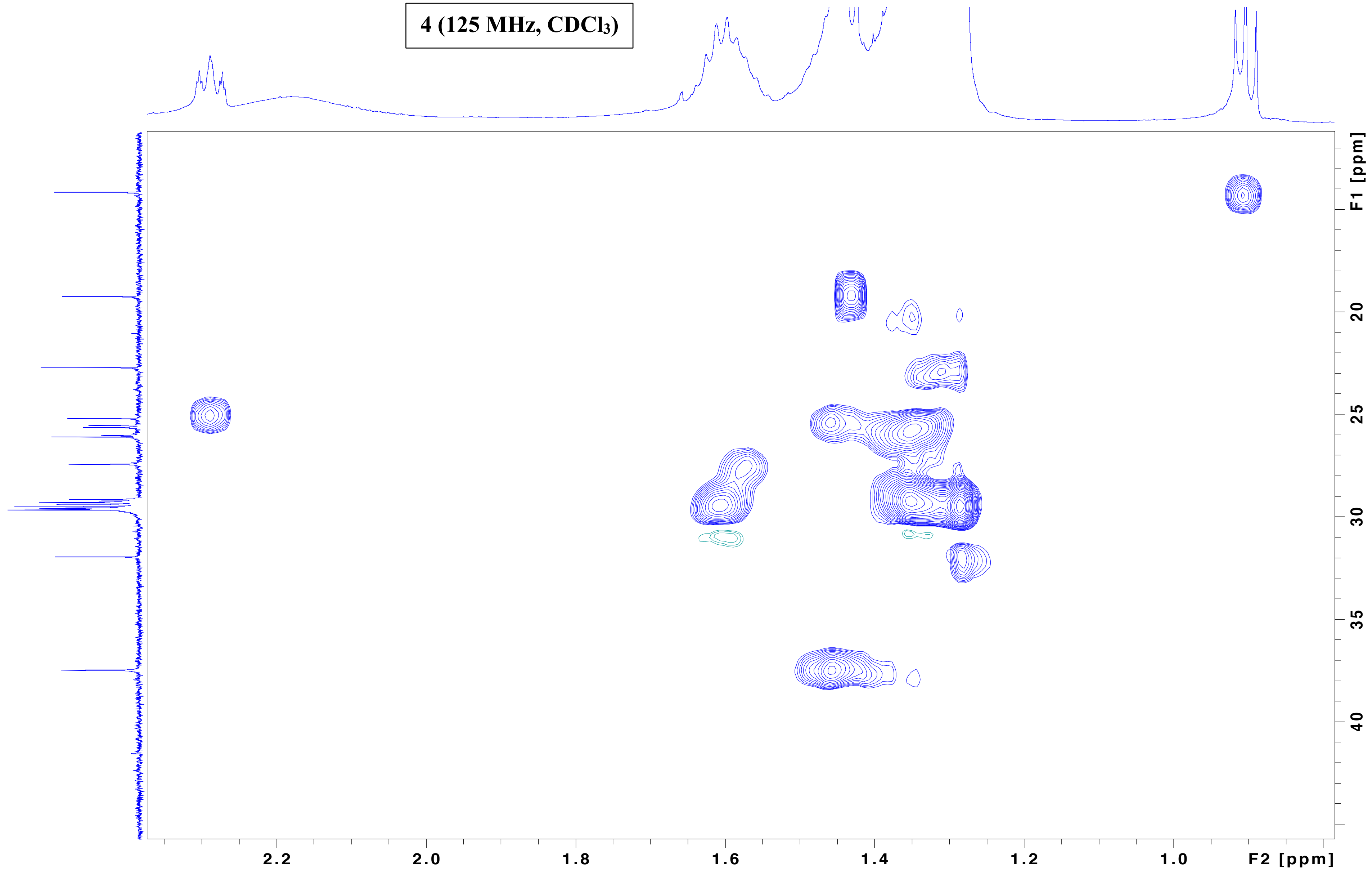
4 (125 MHz, CDCl3)
F1 [ppm]
20
25
30
35
40
2.2
2.0
1.8
1.6
1.4
1.2
1.0
F2 [ppm]

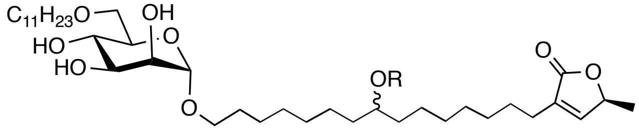
C11H23O
OH
O
HO
HO
O
OR
O
O

4 (125 MHz, CDCl₃)

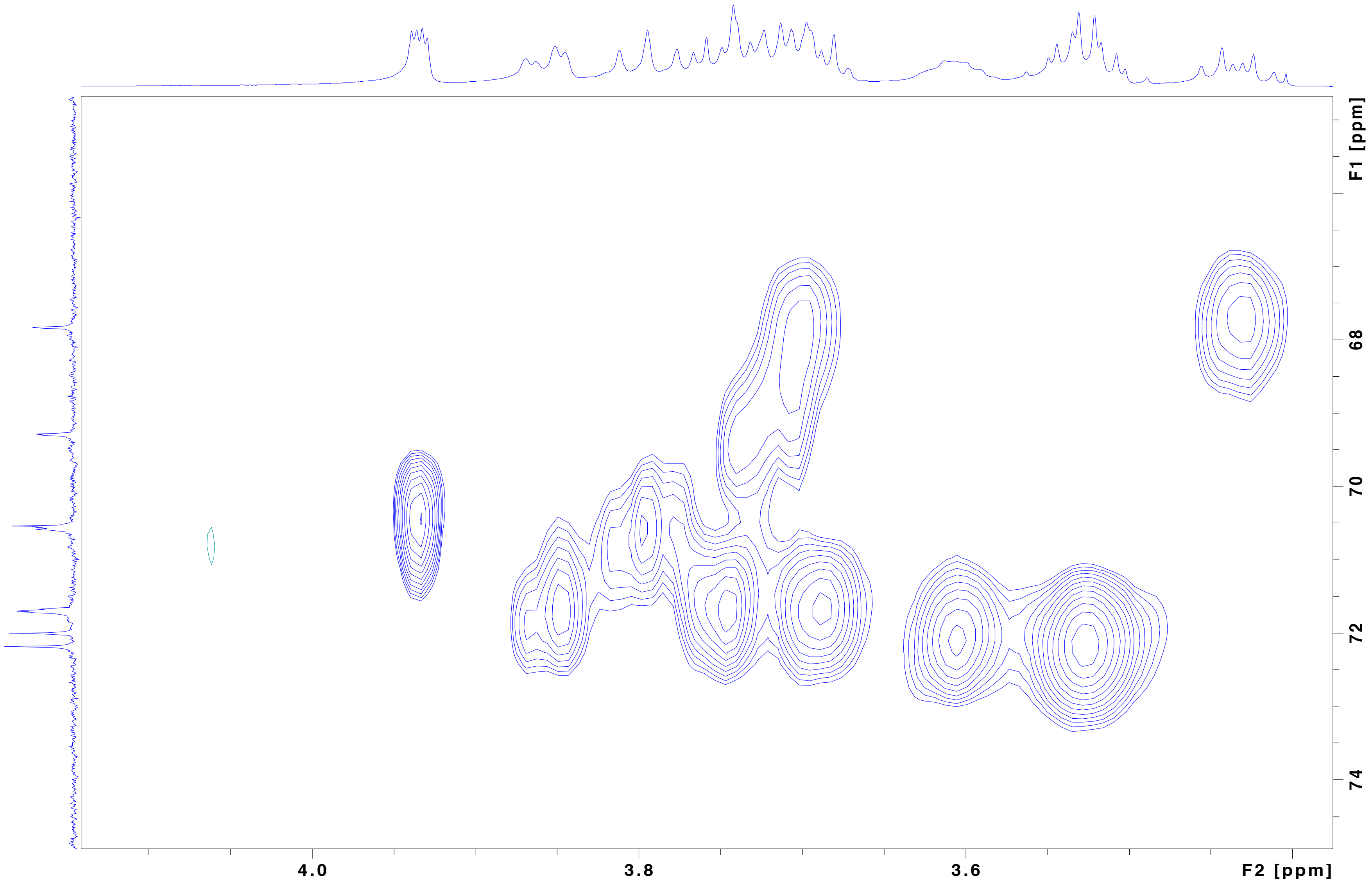

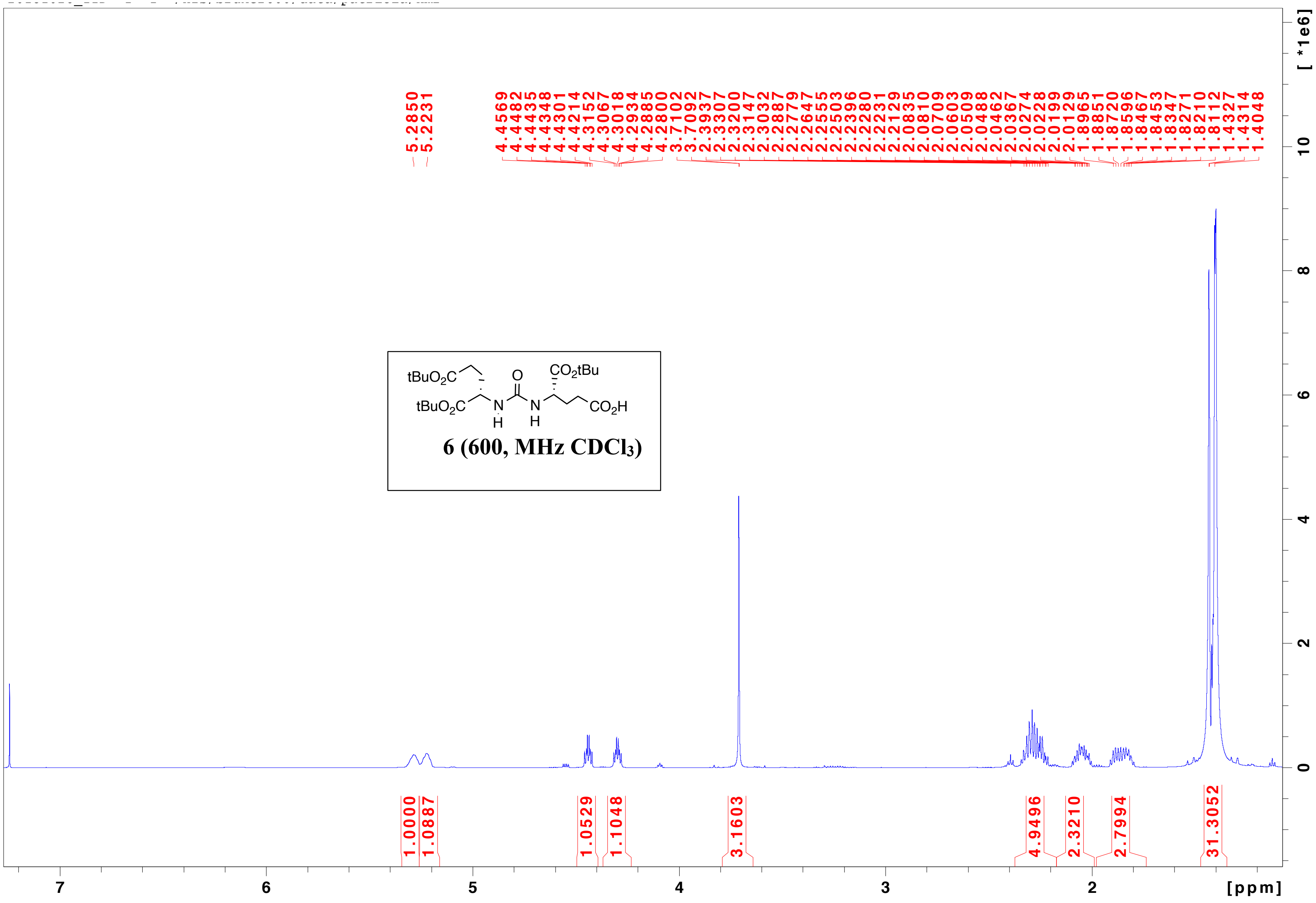
5.2850
5.2231
4.4569
4.4482
4.4435
4.4348
4.4301
4.4214
4.3152
4.3067
4.3018
4.2934
4.2885
4.2800
3.7102
3.7092
2.3937
2.3337
2.3200
2.3147
2.3032
2.2887
2.2779
2.2647
2.2555
2.2503
2.2396
2.2280
2.2231
2.2129
2.0835
2.0810
2.0709
2.0603
2.0509
2.0488
2.0462
2.0367
2.0274
2.0228
2.0199
2.0129
1.8965
1.8851
1.8720
1.8596
1.8467
1.8453
1.8347
1.8271
1.8210
1.8112
1.4327
1.4314
1.4048
tBuO2C
O
CO2tBu
tBuO2C
N
H
N
H
CO2H
6 (600, MHz CDCl3)
1.0000
1.0887
1.0529
1.1048
3.1603
4.9496
2.3210
2.7994
31.3052
7
6
5
4
3
2
[ppm]
[*1e6]
10
8
6
4
2
0

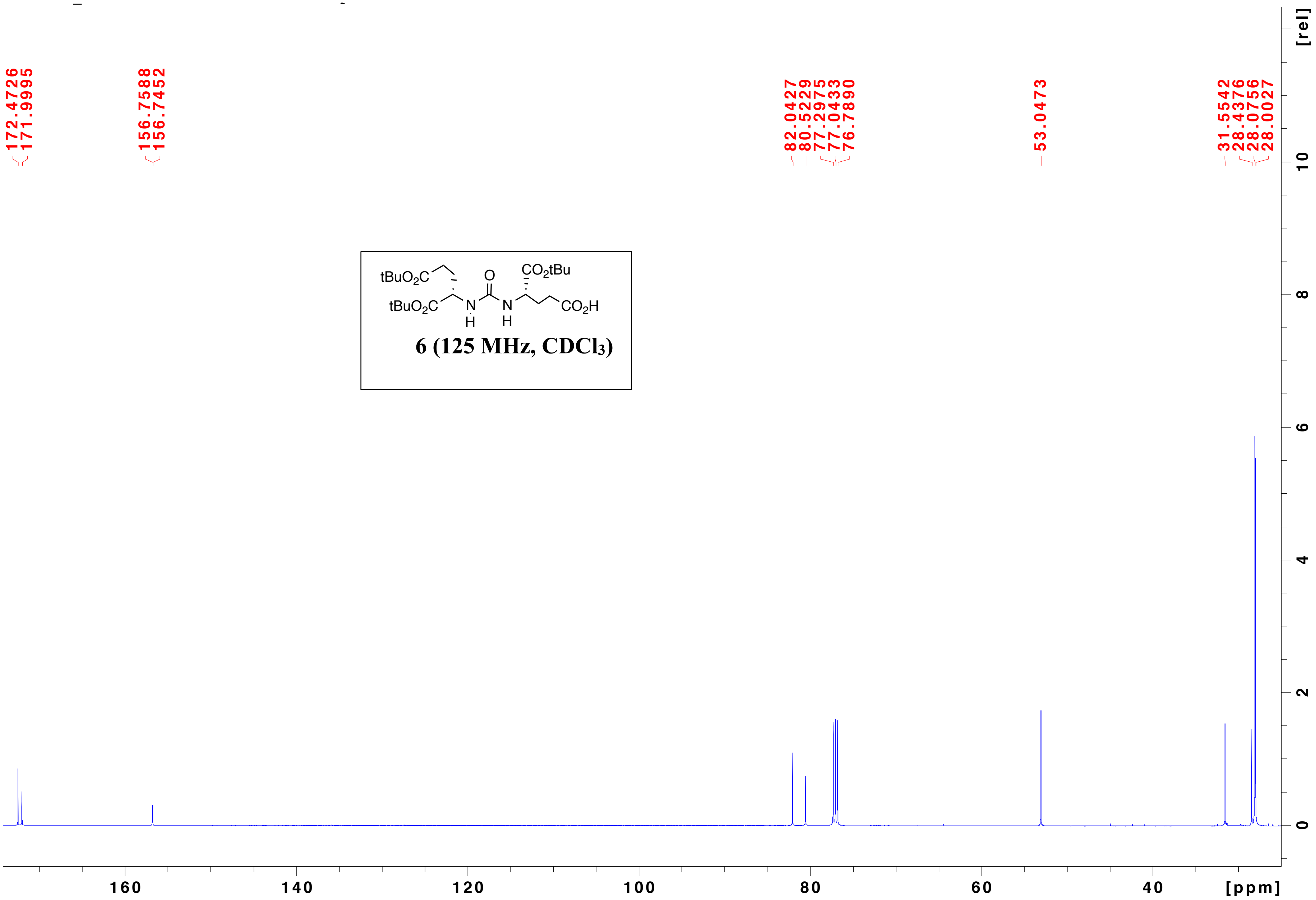
172.4726
171.9995
156.7588
156.7452
82.0427
80.5229
77.2975
77.0433
76.7890
53.0473
31.5542
28.4376
28.0756
28.0027
[rel]
6 (125 MHz, CDCl3)
[ppm]

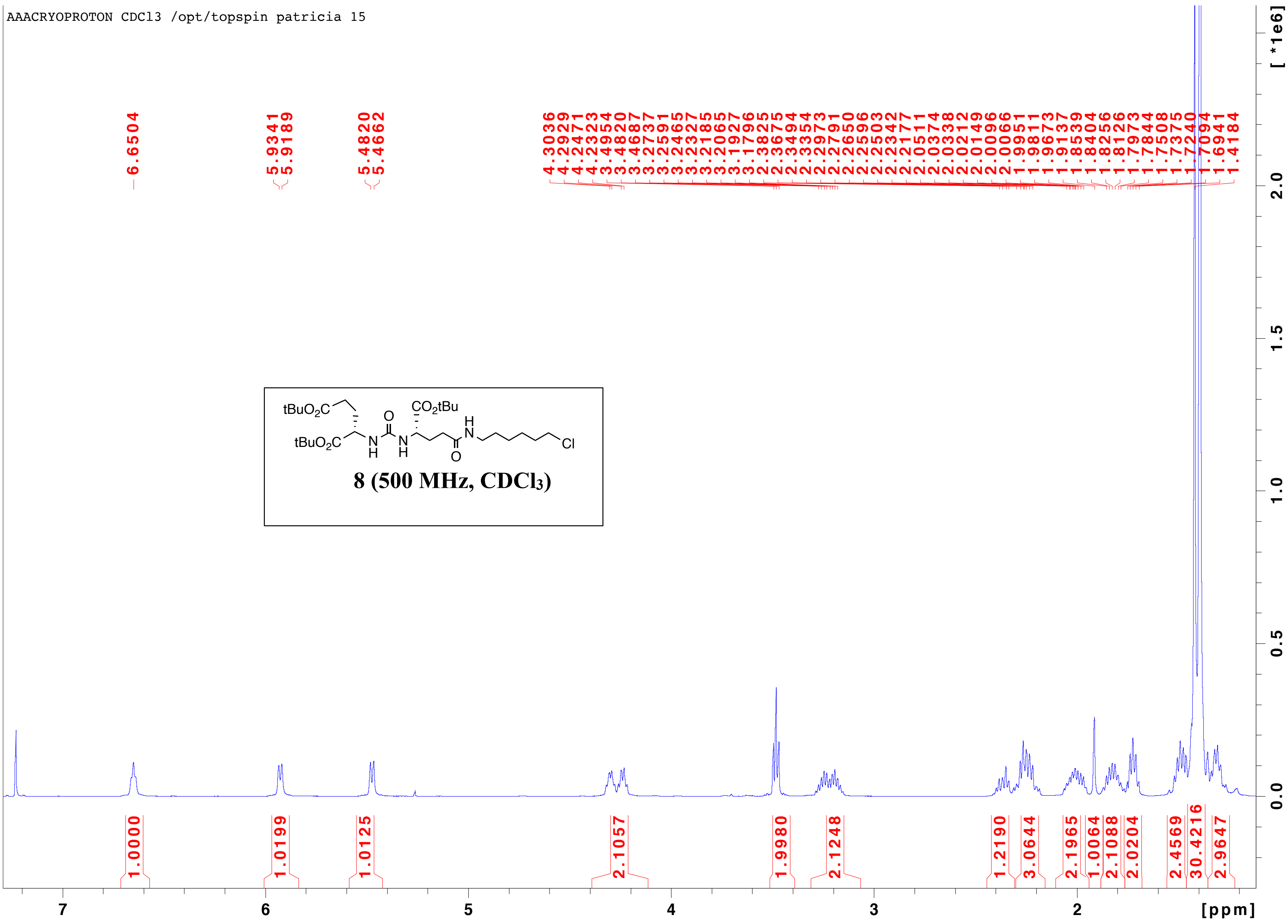
AAACRYOPROTON CDCl3 /opt/topspin patricia 15
8 (500 MHz, CDCl3)
[ppm]
[*1e6]

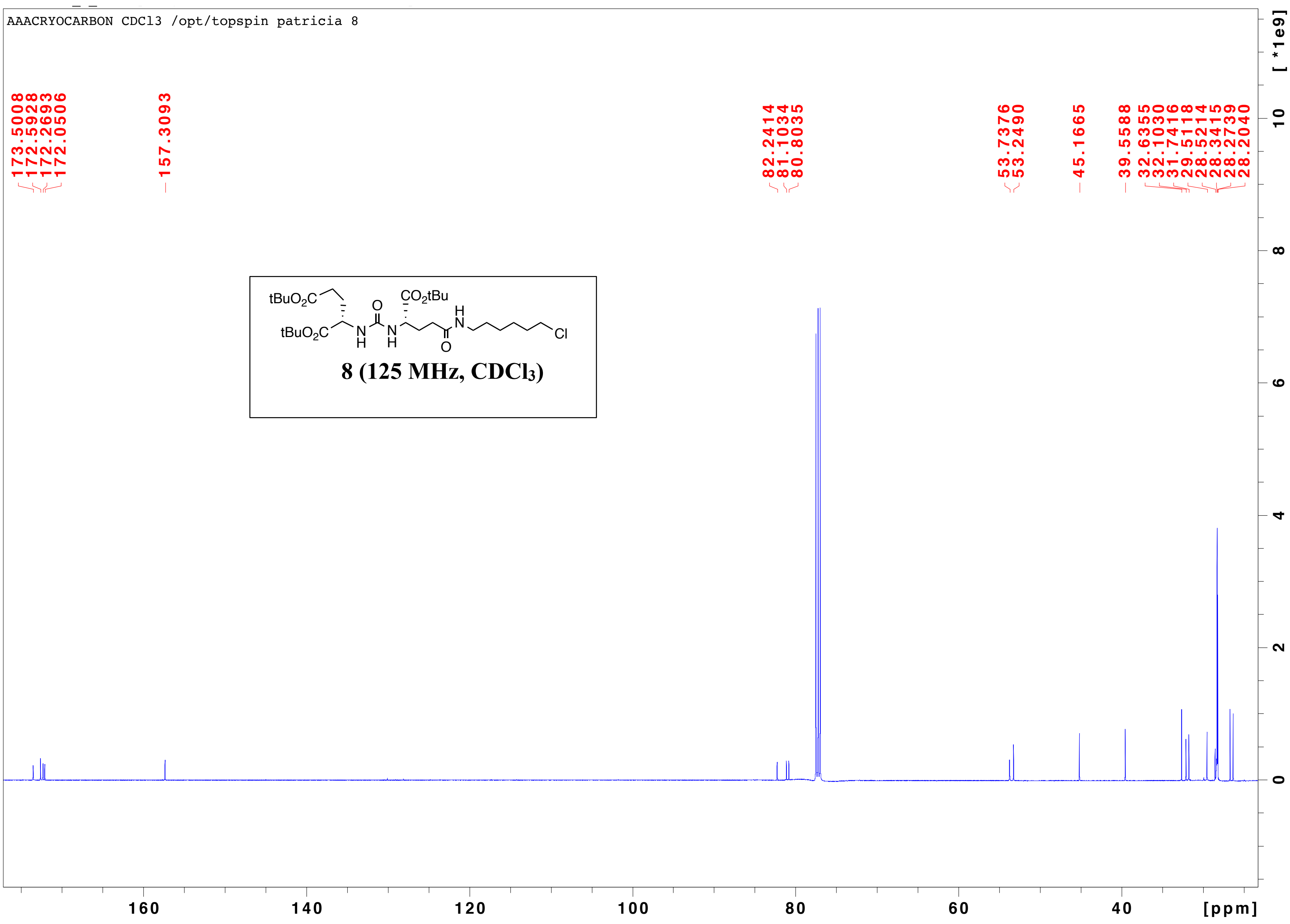
AAACRYOCARBON CDCl3 /opt/topspin patricia 8
173.5008
172.5928
172.2693
172.0506
157.3093
82.2414
81.1034
80.8035
53.7376
53.2490
45.1665
39.5588
32.6355
32.1030
31.7416
29.5118
28.5214
28.3415
28.2739
28.2040
tBuO2C
tBuO2C
CO2tBu
Cl
8 (125 MHz, CDCl3)
[*1e9]
[ppm]
160
140
120
100
80
60
40
10
8
6
4
2
0

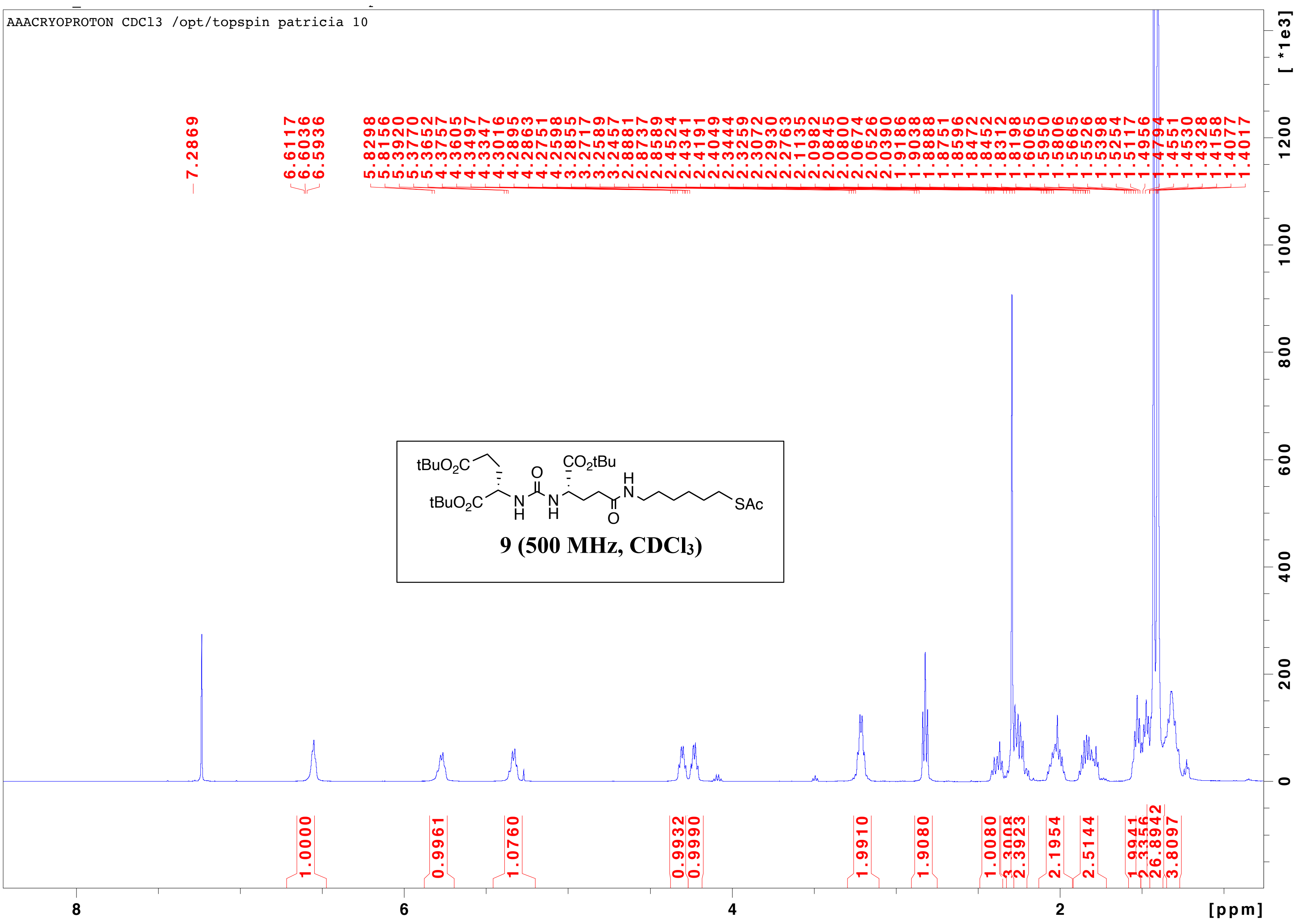
AAACRYOPROTON CDCl3 /opt/topspin patricia 10
7.2869
6.6117
6.6036
6.5936
5.8298
5.8156
5.3920
5.3770
5.3652
4.3757
4.3605
4.3497
4.3347
4.3016
4.2895
4.2863
4.2751
4.2598
3.2855
3.2717
3.2589
3.2457
2.8881
2.8737
2.8589
2.4524
2.4341
2.4191
2.4049
2.3444
2.3259
2.3072
2.2930
2.2763
2.2135
2.0982
2.0845
2.0674
2.0526
2.0390
1.9186
1.9038
1.8888
1.8751
1.8596
1.8472
1.8452
1.8312
1.8198
1.6065
1.5950
1.5806
1.5665
1.5526
1.5398
1.5254
1.5117
1.4956
1.4794
1.4551
1.4530
1.4328
1.4158
1.4077
1.4017
tBuO2C
CO2tBu
O
H
N
N
N
H
H
O
tBuO2C
SAc
9 (500 MHz, CDCl3)
1.0000
0.9961
1.0760
0.9932
0.9990
1.9910
1.9080
1.0080
2.3923
2.1954
2.5144
26.8942
3.8097
[*1e3]
1200
1000
800
600
400
200
0
8
6
4
2
[ppm]

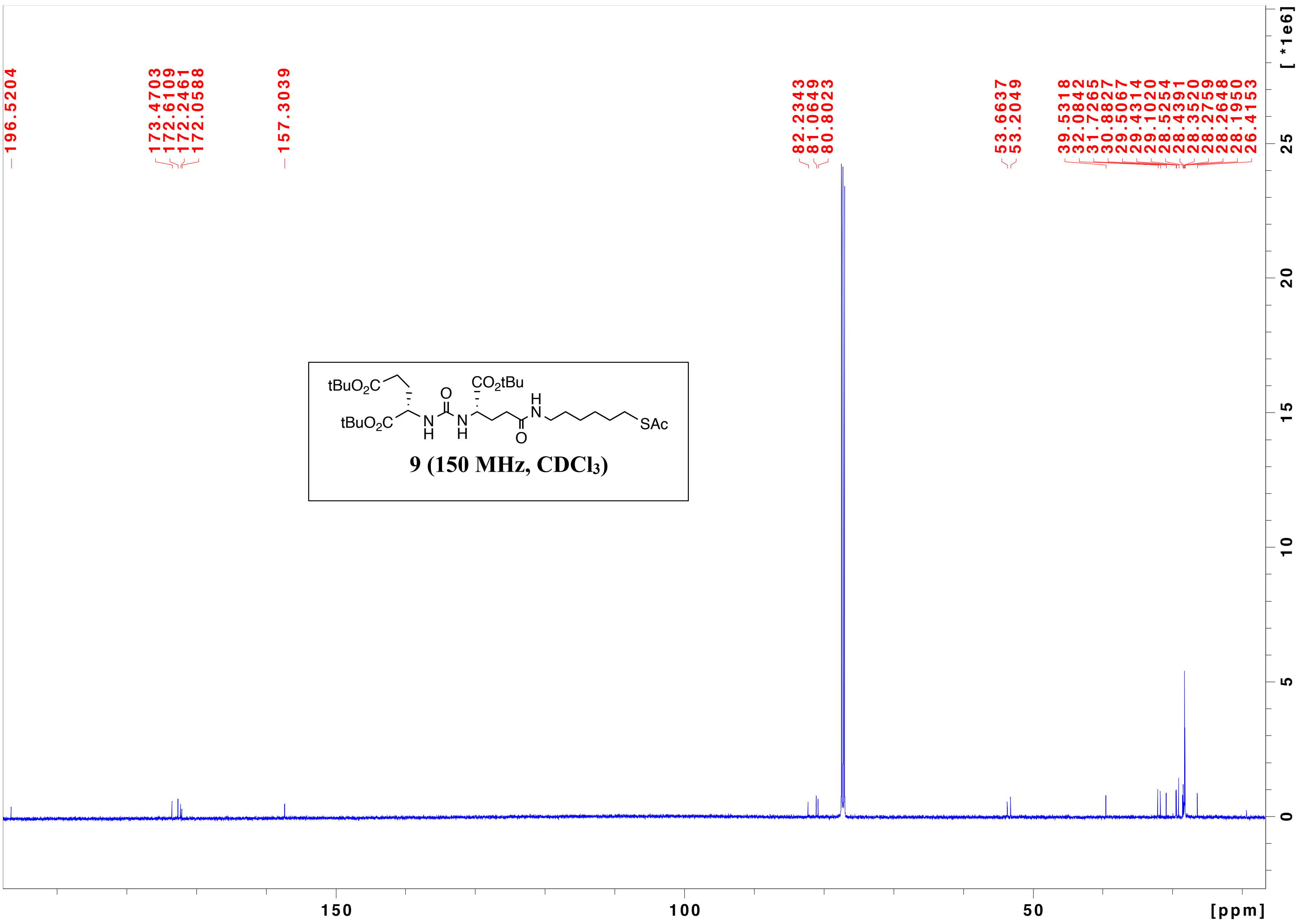
196.5204
173.4703
172.6109
172.2461
172.0588
157.3039
82.2343
81.0649
80.8023
53.6637
53.2049
39.5318
32.0842
31.7265
30.8827
29.5067
29.4314
29.1020
28.5254
28.4391
28.3520
28.2759
28.2648
28.1950
26.4153
9 (150 MHz, CDCl3)
[ppm]
[*1e6]

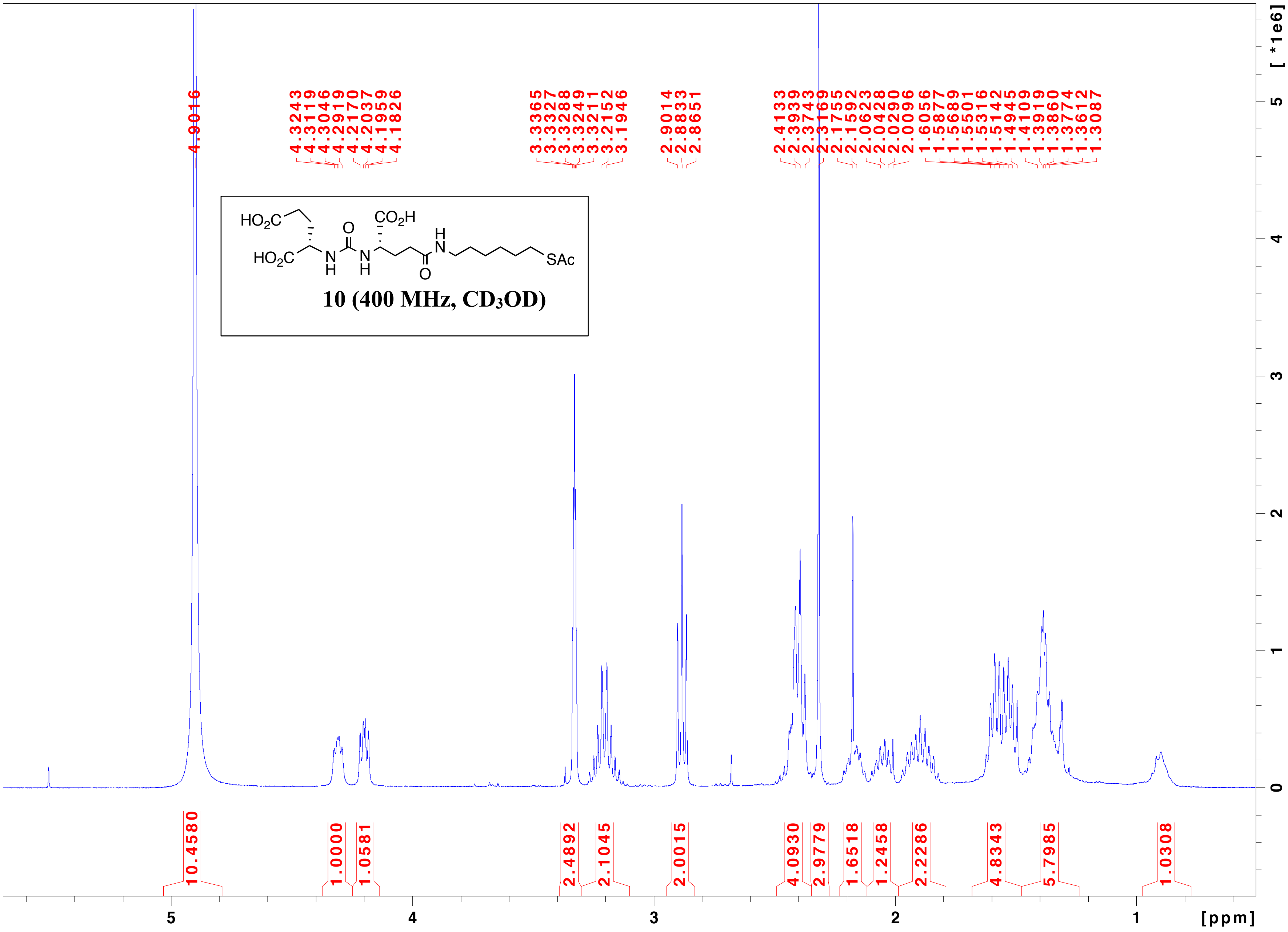

4.9016
4.3243
4.3119
4.3046
4.2919
4.2170
4.2037
4.1959
4.1826
3.3365
3.3327
3.3288
3.3249
3.3211
3.2152
3.1946
2.9014
2.8833
2.8651
2.4133
2.3939
2.3743
2.3169
2.1755
2.1592
2.0623
2.0428
2.0290
2.0096
1.6056
1.5877
1.5689
1.5501
1.5316
1.5142
1.4945
1.4109
1.3919
1.3860
1.3774
1.3612
1.3087
HO2C
CO2H
O
H
N
HO2C
N
H
N
H
O
SAc
10 (400 MHz, CD3OD)
10.4580
1.0000
1.0581
2.4892
2.1045
2.0015
4.0930
2.9779
1.6518
1.2458
2.2286
4.8343
5.7985
1.0308
5
4
3
2
1
[ppm]
[*1e6]

10 (400 MHz, CD$_3$OD)

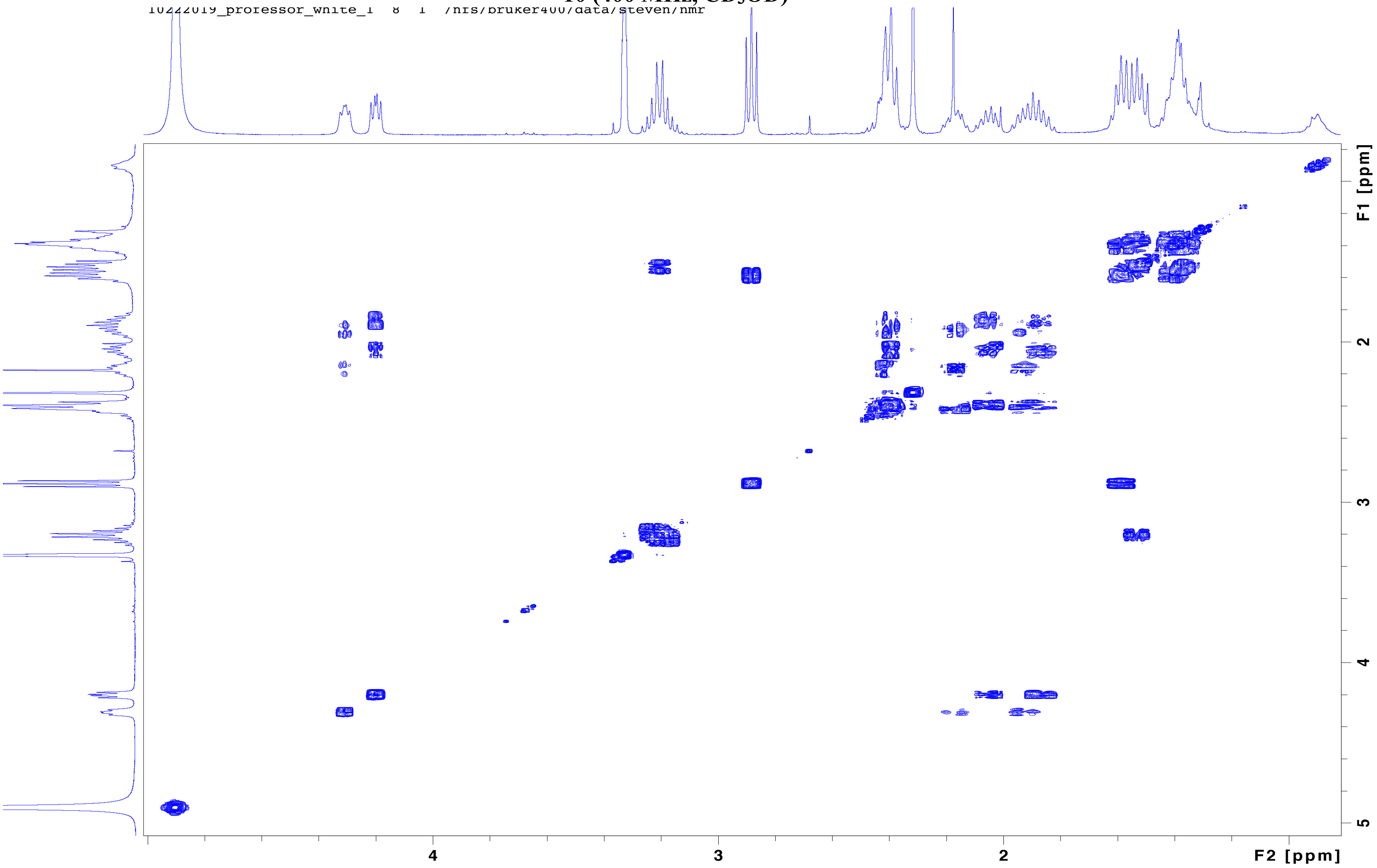

10 (400 MHz, CD_3OD)

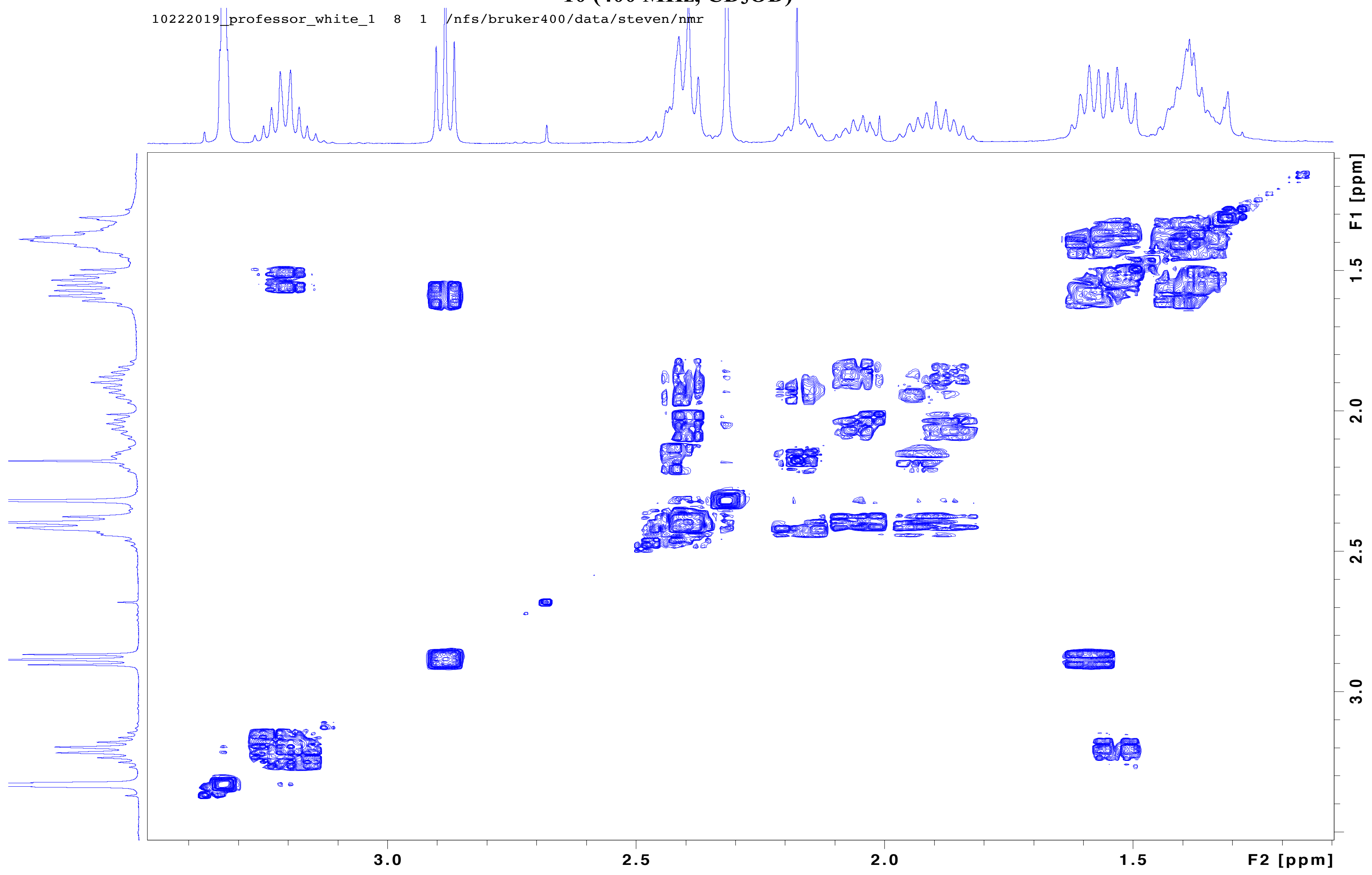

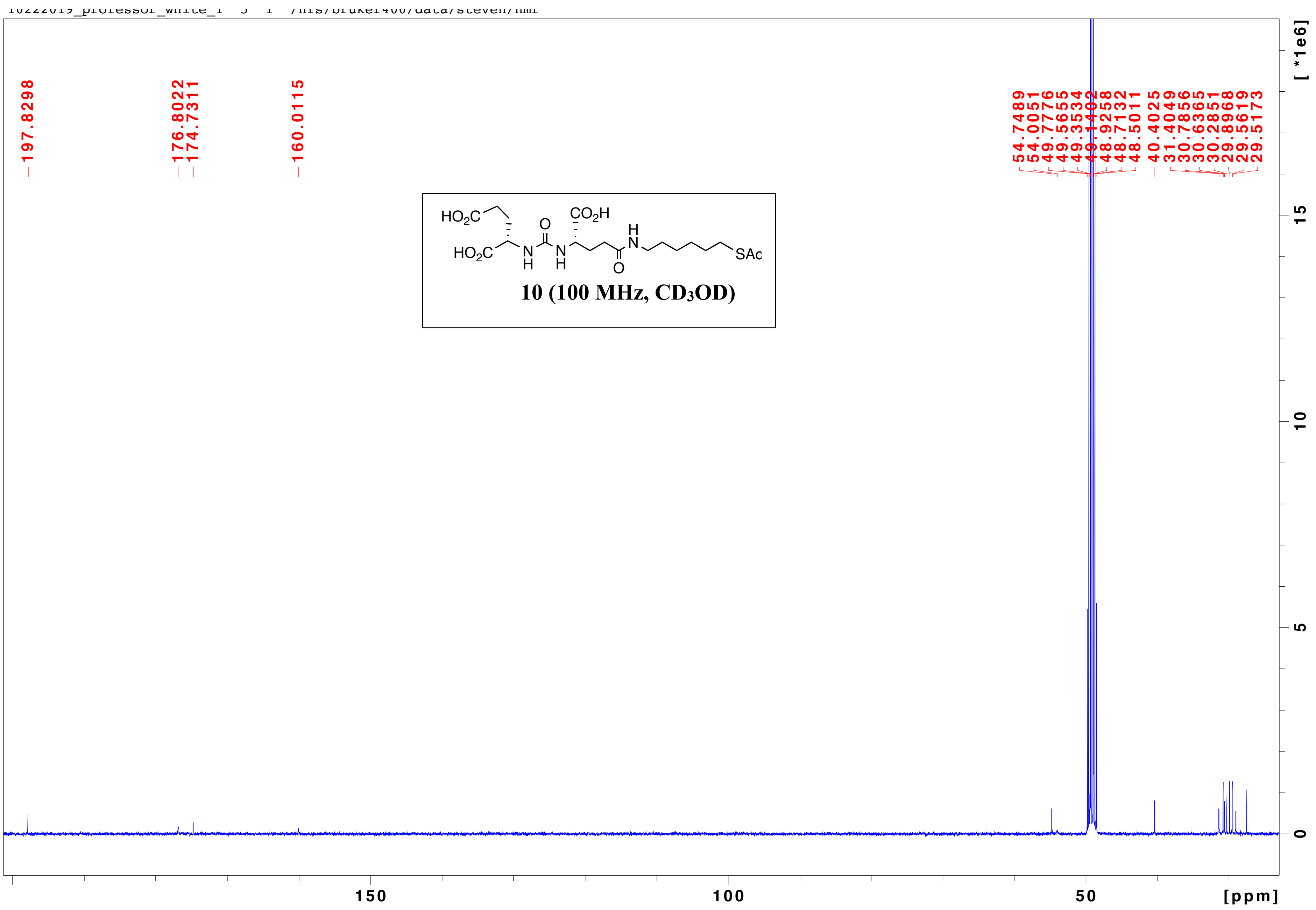

10222019_professor_white_1 5 1 /nfs/bruker400/data/steven/nmr
197.8298
176.8022
174.7311
160.0115
54.7489
54.0051
49.7776
49.5655
49.3534
49.1402
48.9258
48.7132
48.5011
40.4025
31.4049
30.7856
30.6365
30.2851
29.8968
29.5619
29.5173
HO2C
CO2H
O
H
N
HO2C
N
H
N
H
SAc
O
10 (100 MHz, CD3OD)
[*1e6]
15
10
5
0
150
100
50
[ppm]

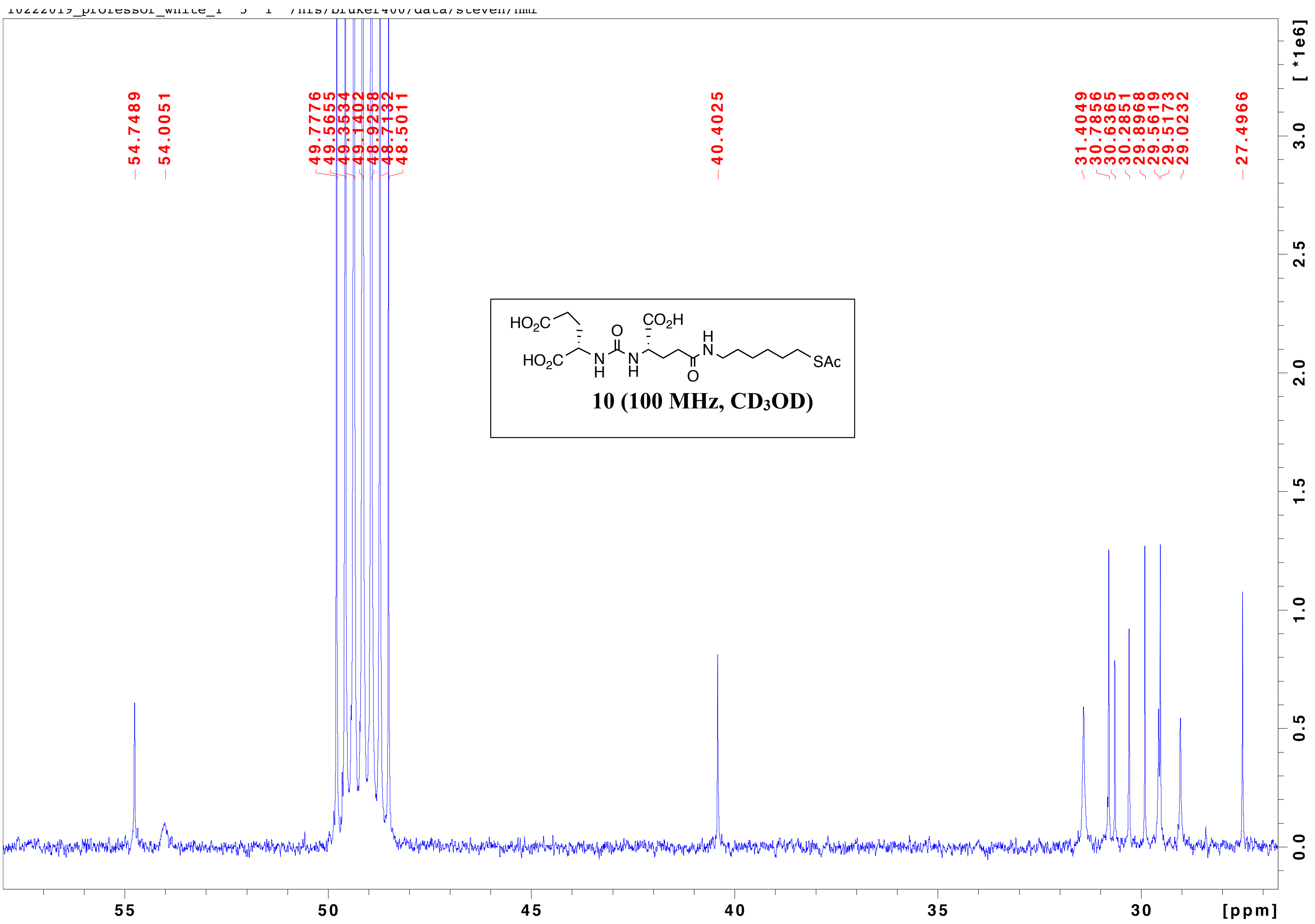
10222019_professor_white_1 5 1 /nfs/bruker400/data/steven/nmr
54.7489
54.0051
49.7776
49.5655
49.3534
49.1402
48.9258
48.7132
48.5011
40.4025
31.4049
30.7856
30.6365
30.2851
29.8968
29.5619
29.5173
29.0232
27.4966
[*1e6]
3.0
2.5
2.0
1.5
1.0
0.5
0.0
55
50
45
40
35
30
[ppm]
HO2C
CO2H
O
N
H
SAc
10 (100 MHz, CD3OD)

\

10 (400 MHz, CD$_3$OD)

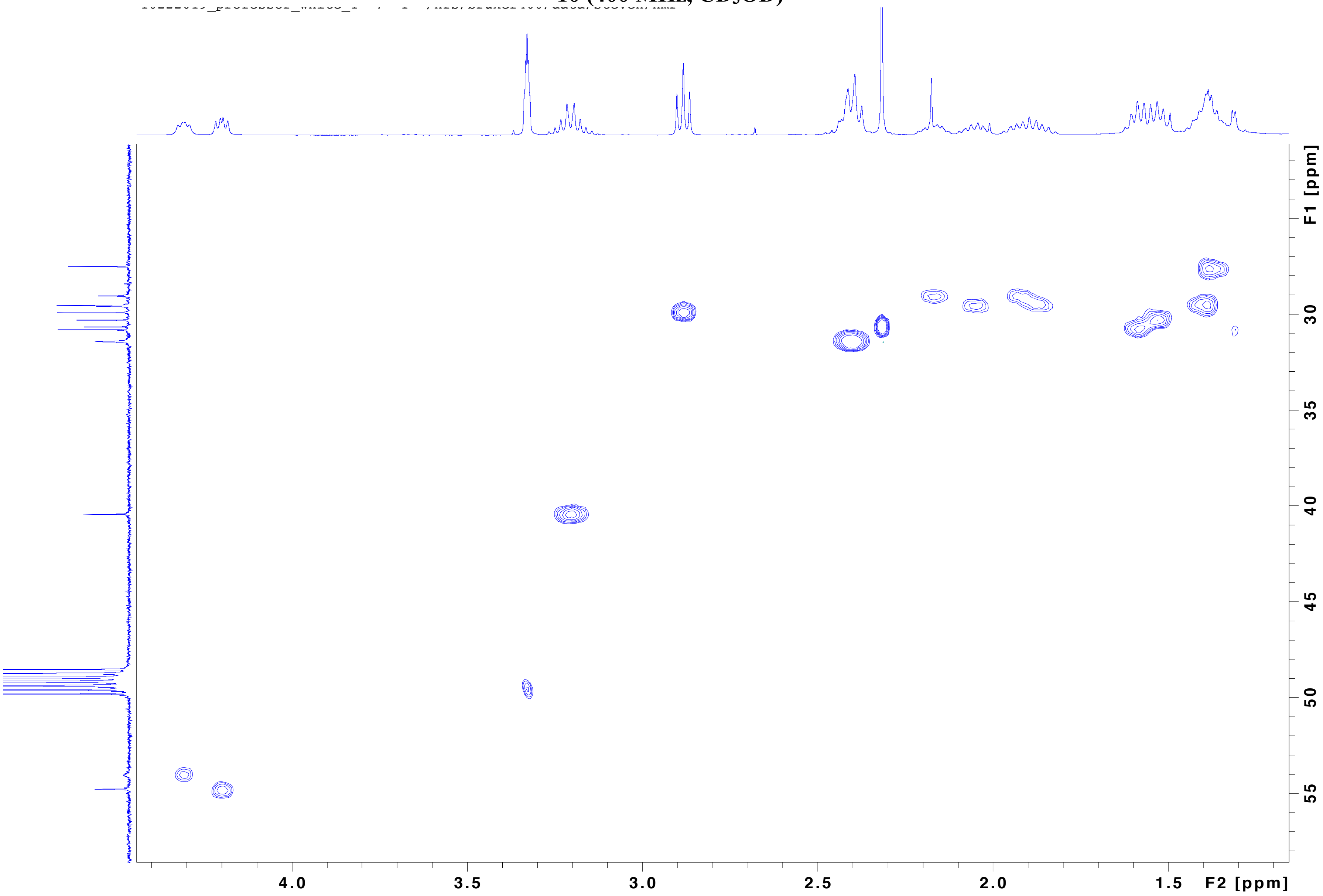

10 (400 MHz, CD$_3$OD)

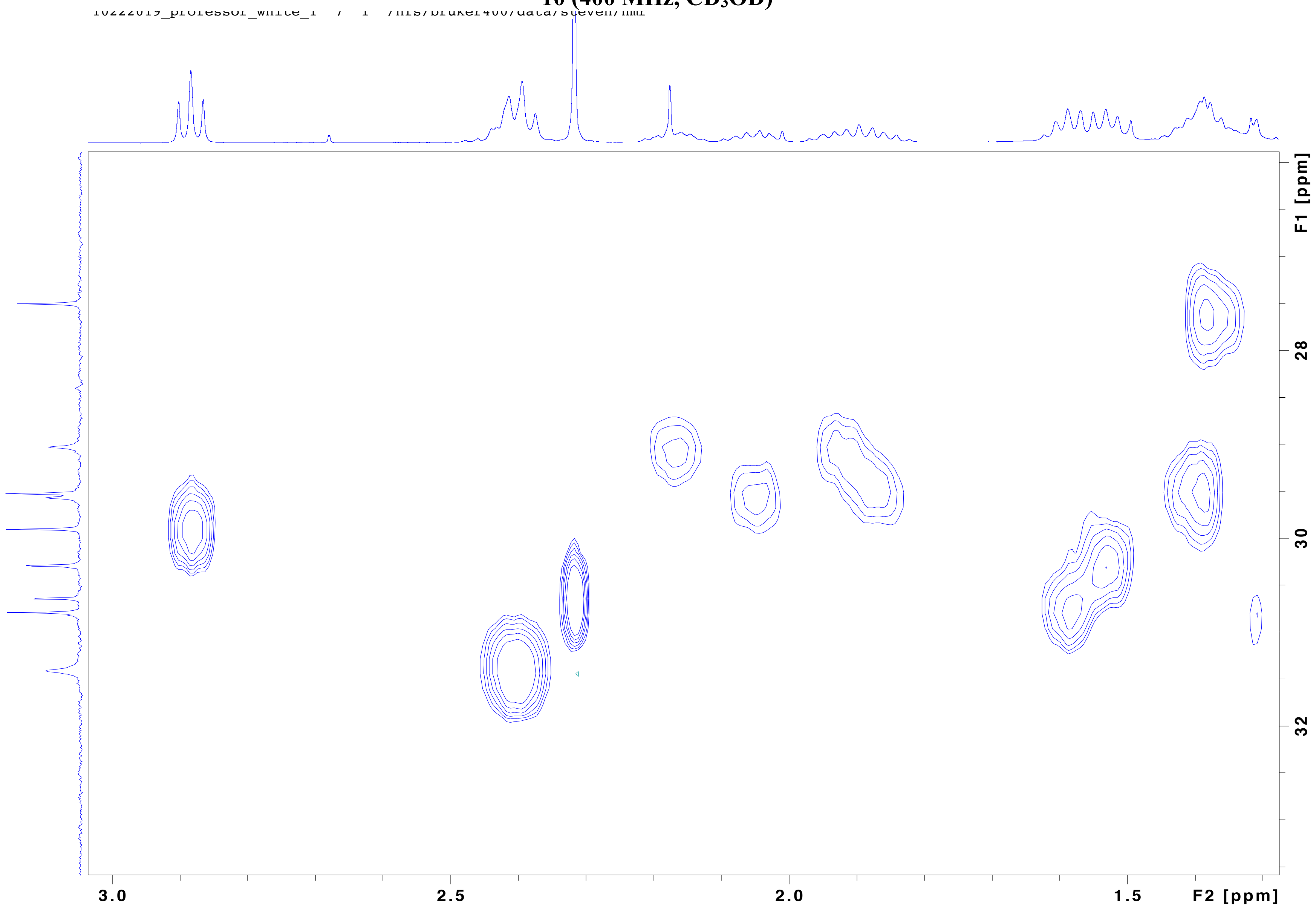

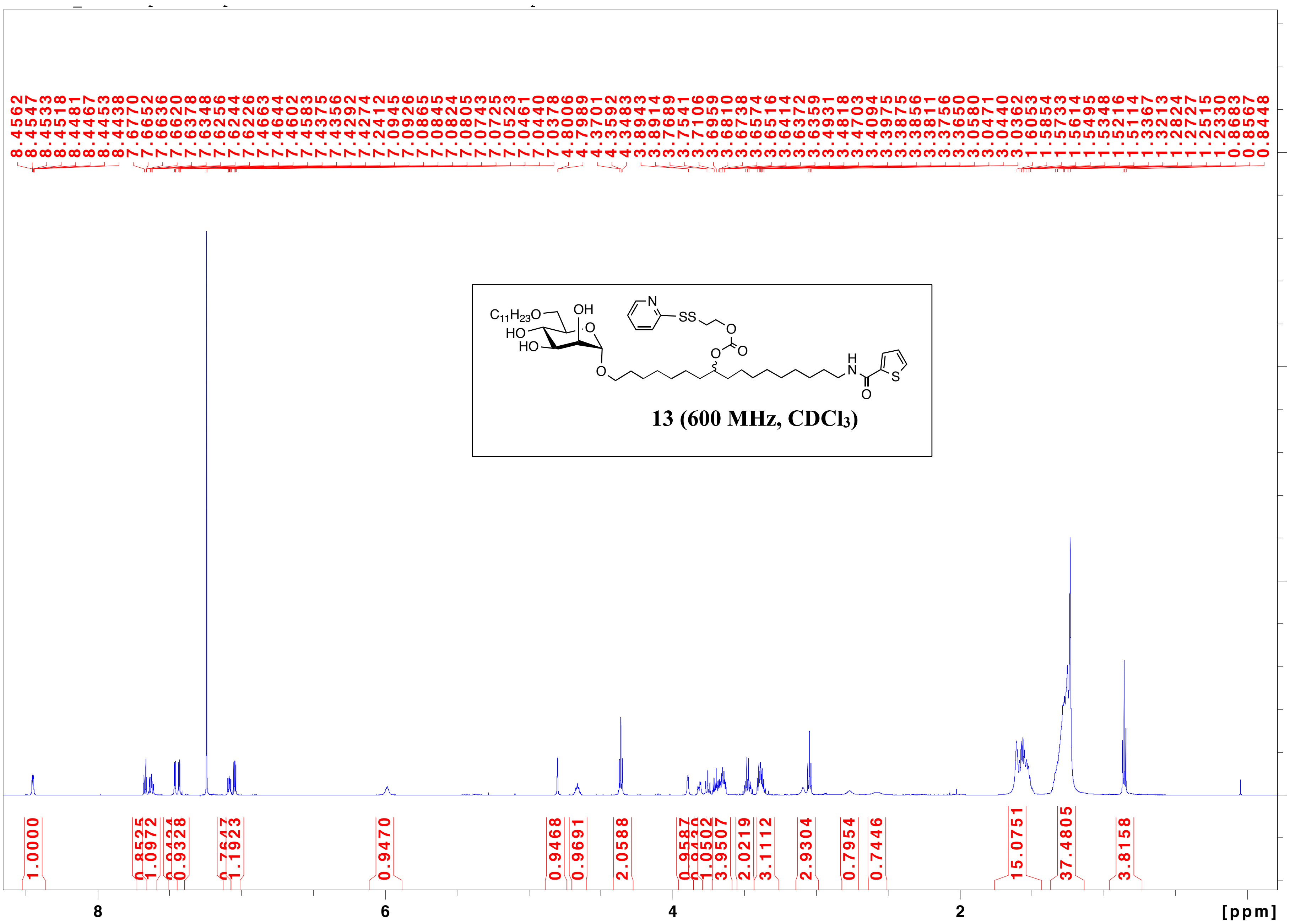
13 (600 MHz, CDCl3)
[ppm]

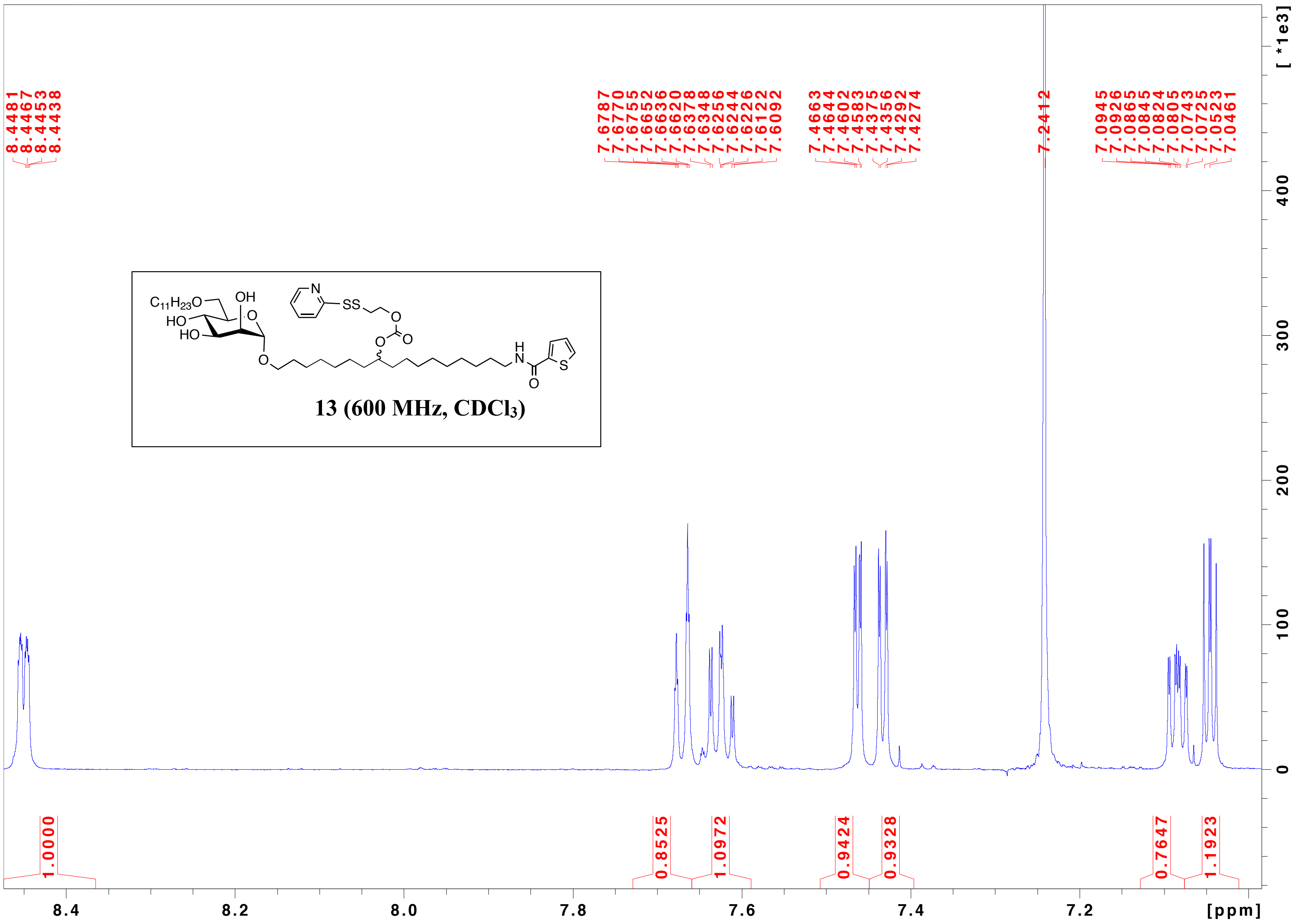
8.4481
8.4467
8.4453
8.4438
7.6787
7.6770
7.6755
7.6652
7.6636
7.6620
7.6378
7.6348
7.6256
7.6244
7.6226
7.6222
7.6122
7.6092
7.4663
7.4644
7.4602
7.4583
7.4375
7.4356
7.4292
7.4274
7.2412
7.0945
7.0926
7.0865
7.0845
7.0824
7.0805
7.0743
7.0725
7.0523
7.0461
$C_{11}H_{23}O$
OH
O
HO
HO
N
SS
O
O
O
O
H
N
S
O
13 (600 MHz, CDCl3)
1.0000
0.8525
1.0972
0.9424
0.9328
0.7647
1.1923
[*1e3]
400
300
200
100
0
8.4
8.2
8.0
7.8
7.6
7.4
7.2
[ppm]

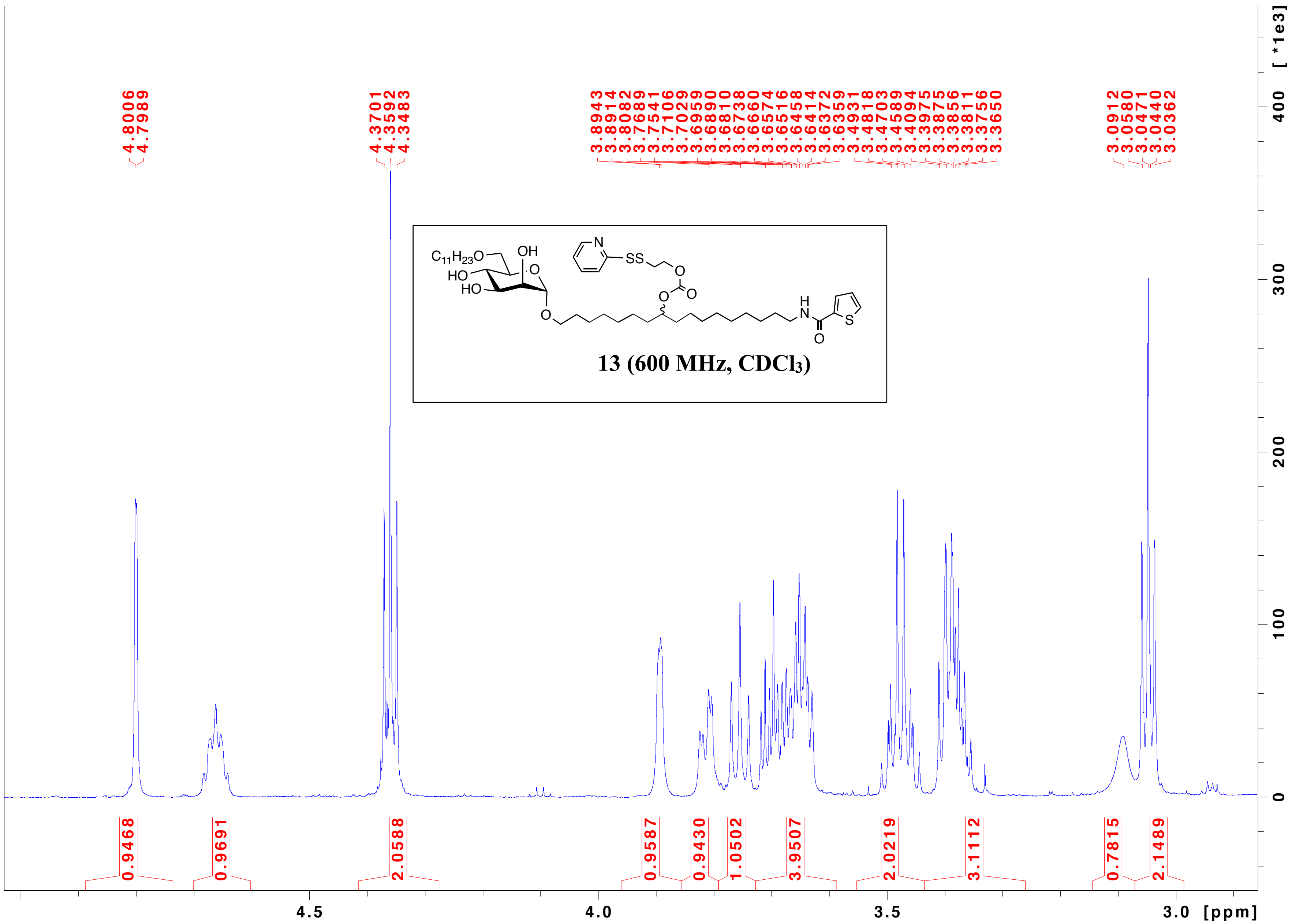
13 (600 MHz, CDCl3)
4.5
4.0
3.5
3.0 [ppm]
[*1e3]

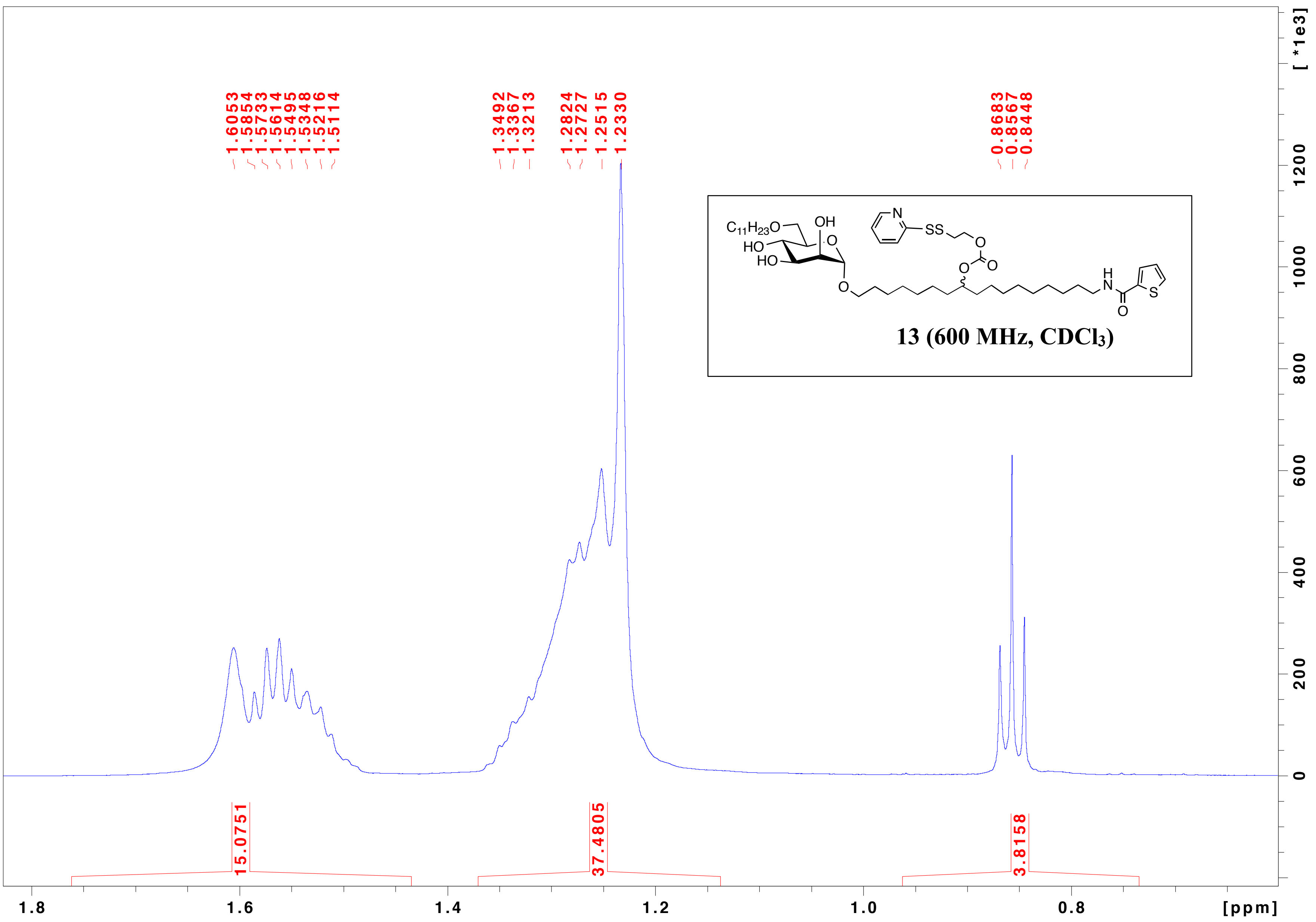
1.6053
1.5854
1.5733
1.5614
1.5495
1.5348
1.5216
1.5114
1.3492
1.3367
1.3213
1.2824
1.2727
1.2515
1.2330
0.8683
0.8567
0.8448
15.0751
37.4805
3.8158
[*1e3]
1200
1000
800
600
400
200
0
1.8
1.6
1.4
1.2
1.0
0.8
[ppm]
$C_{11}H_{23}O$
OH
O
HO
HO
N
SS
O
O
O
O
H
N
S
O
13 (600 MHz, $CDCl_3$)

13 (600 MHz, CDCl$_3$)

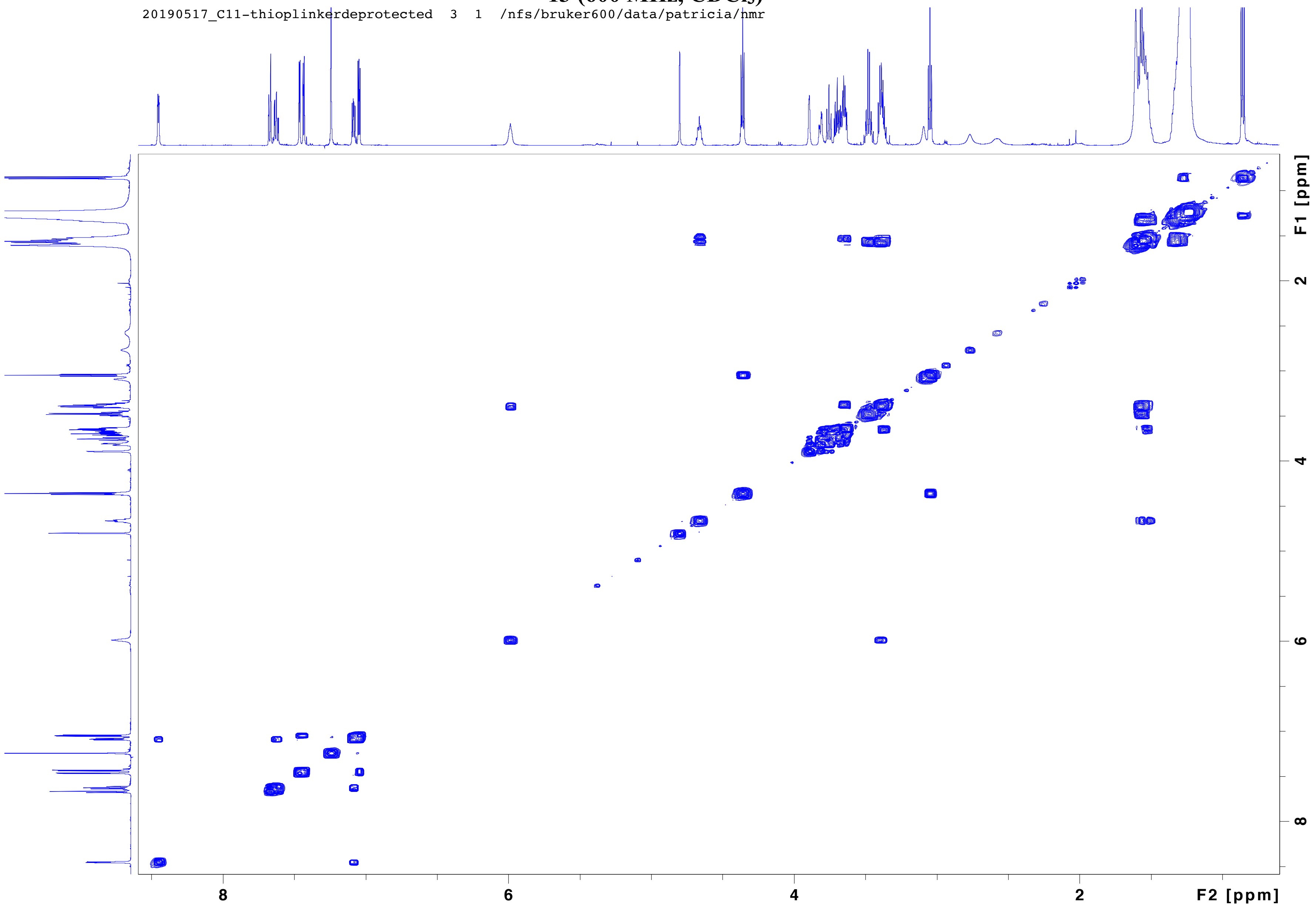

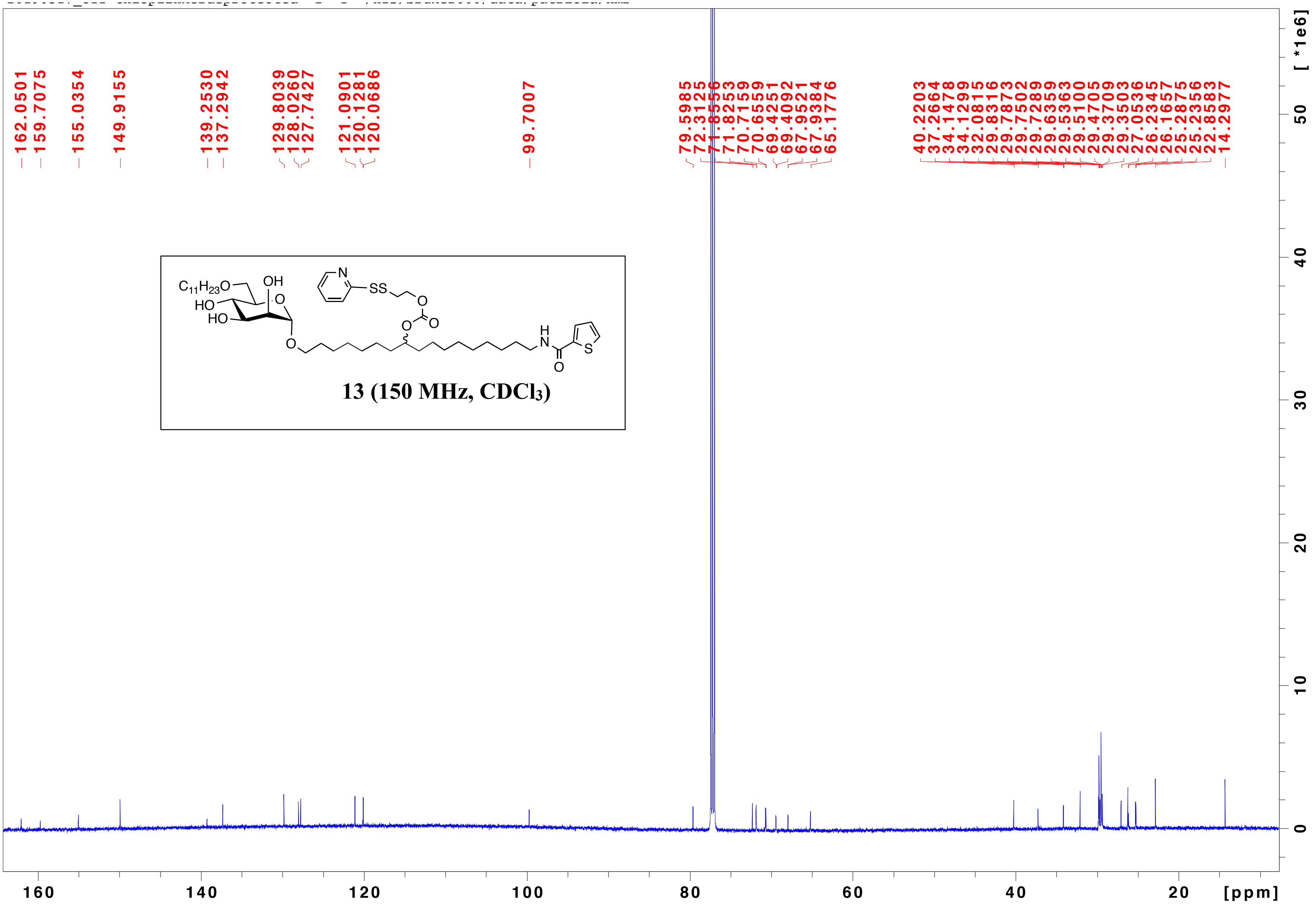
162.0501
159.7075
155.0354
149.9155
139.2530
137.2942
129.8039
128.0260
127.7427
121.0901
120.1281
120.0686
99.7007
79.5985
72.3125
71.8556
71.8253
70.7159
70.6559
69.4251
69.4092
67.9521
67.9384
65.1776
40.2203
37.2664
34.1478
34.1299
32.0815
29.8316
29.7873
29.7502
29.7289
29.6359
29.5363
29.5100
29.4705
29.3709
29.3503
27.0536
26.2345
26.1657
25.2875
25.2356
22.8583
14.2977
[*1e6]
50
40
30
20
10
0
160
140
120
100
80
60
40
20
[ppm]
$C_{11}H_{23}O$
OH
HO
HO
O
N
SS
O
O
O
O
H
N
O
S
13 (150 MHz, $CDCl_3$)

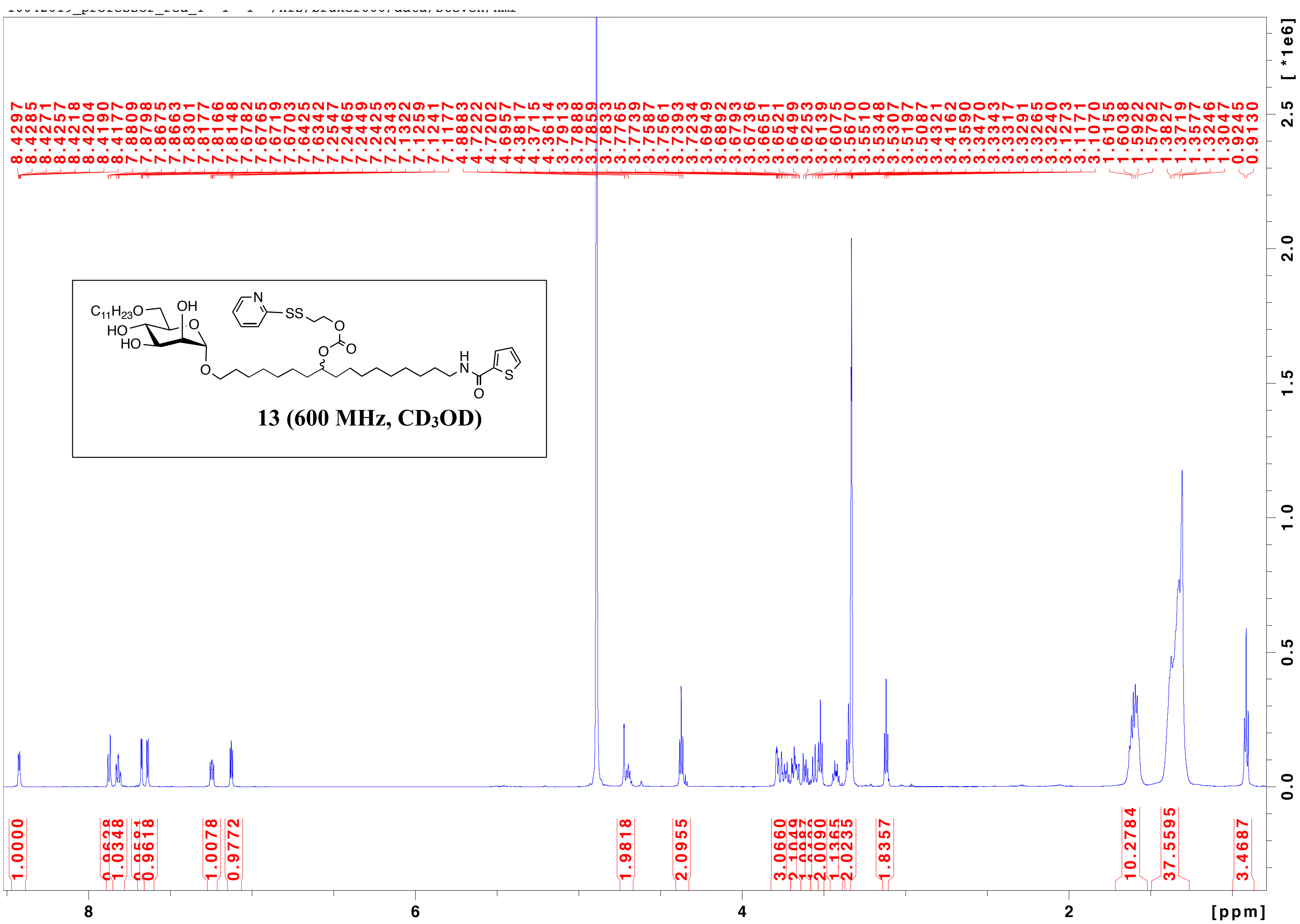

13 (600 MHz, CD3OD)
[*1e6]
[ppm]
1.0000
1.0348
0.9618
1.0078
0.9772
1.9818
2.0955
3.0660
2.0090
1.1365
2.0235
1.8357
10.2784
37.5595
3.4687

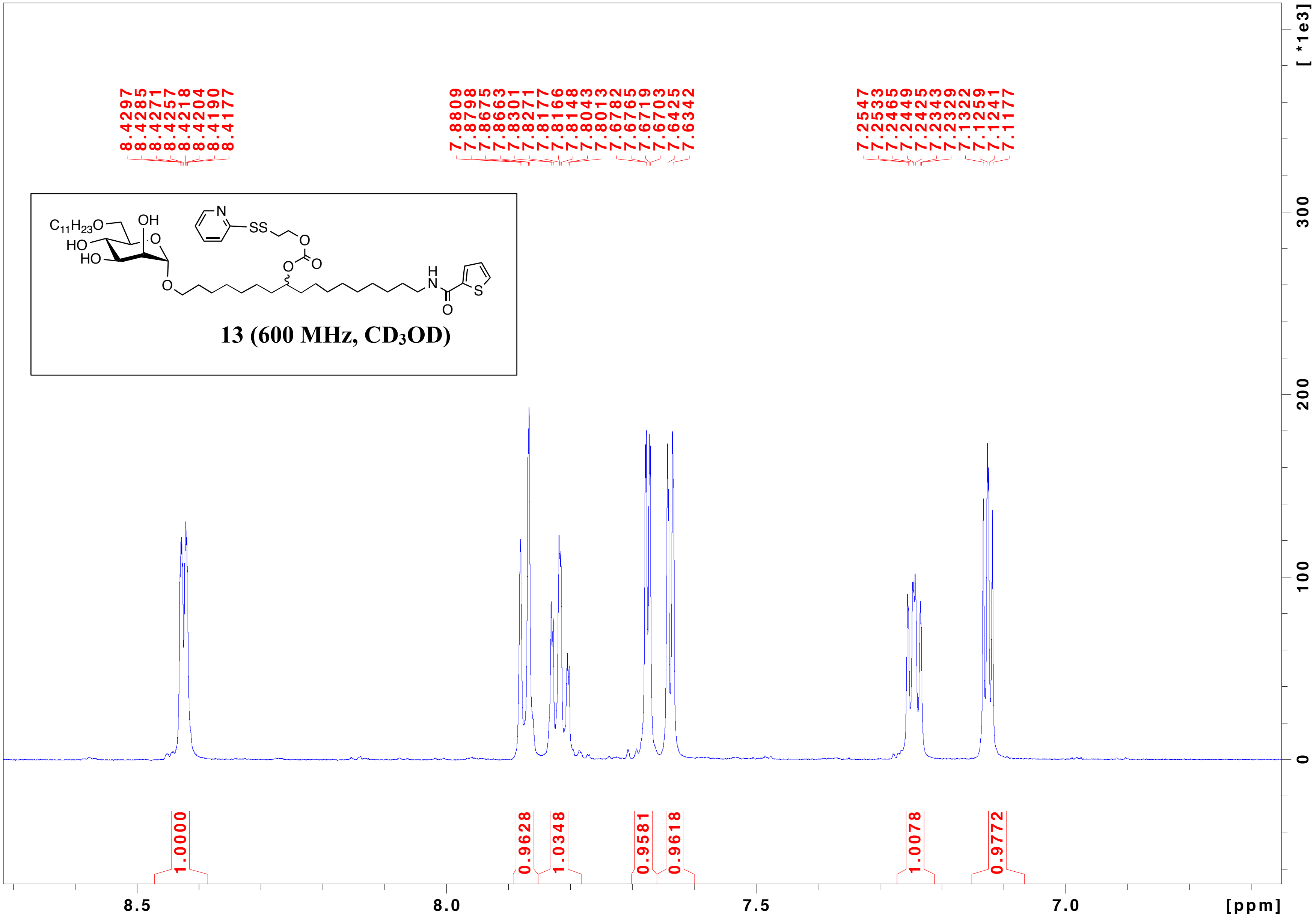
13 (600 MHz, CD3OD)
8.4297
8.4285
8.4271
8.4257
8.4218
8.4204
8.4190
8.4177
7.8809
7.8798
7.8675
7.8663
7.8301
7.8271
7.8177
7.8166
7.8148
7.8043
7.8013
7.6782
7.6765
7.6719
7.6703
7.6425
7.6342
7.2547
7.2535
7.2465
7.2449
7.2425
7.2343
7.2329
7.1322
7.1259
7.1241
7.1177
1.0000
0.9628
1.0348
0.9581
0.9618
1.0078
0.9772
8.5
8.0
7.5
7.0
[ppm]
[*1e3]
300
200
100
0

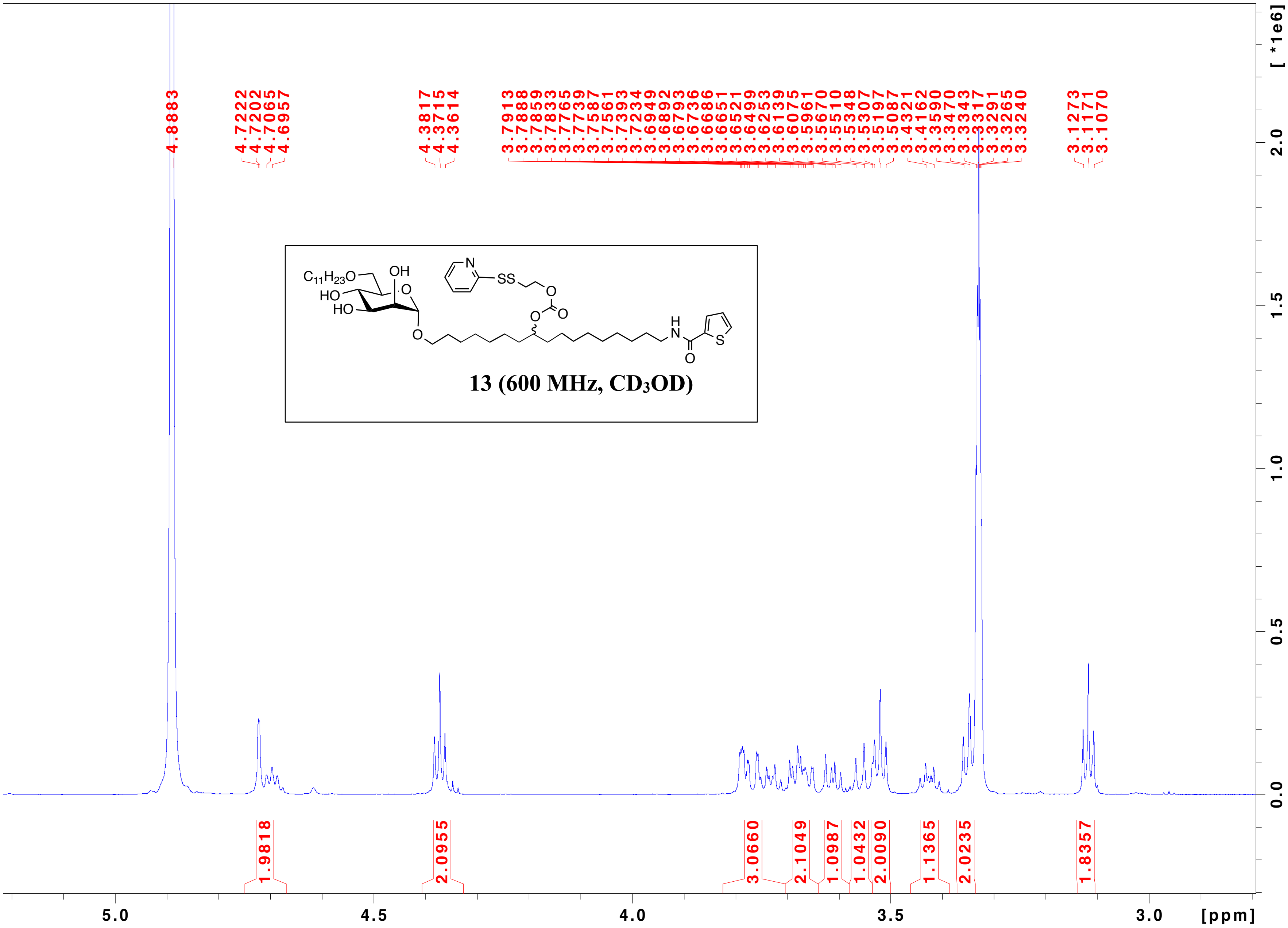
13 (600 MHz, CD3OD)
[ppm]
[*1e6]

13 (600 MHz, CD_3OD)

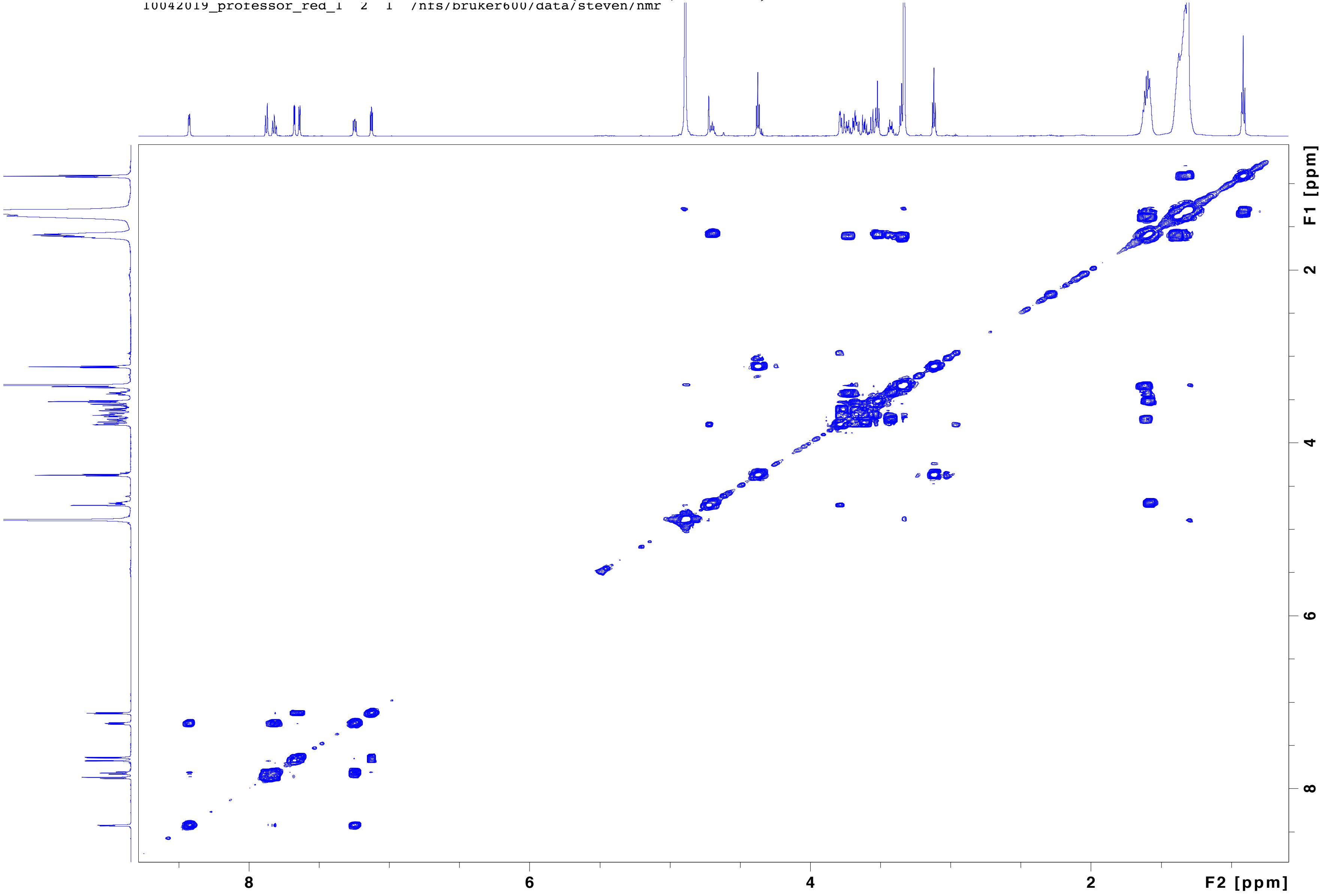

13 (600 MHz, CD_3OD)

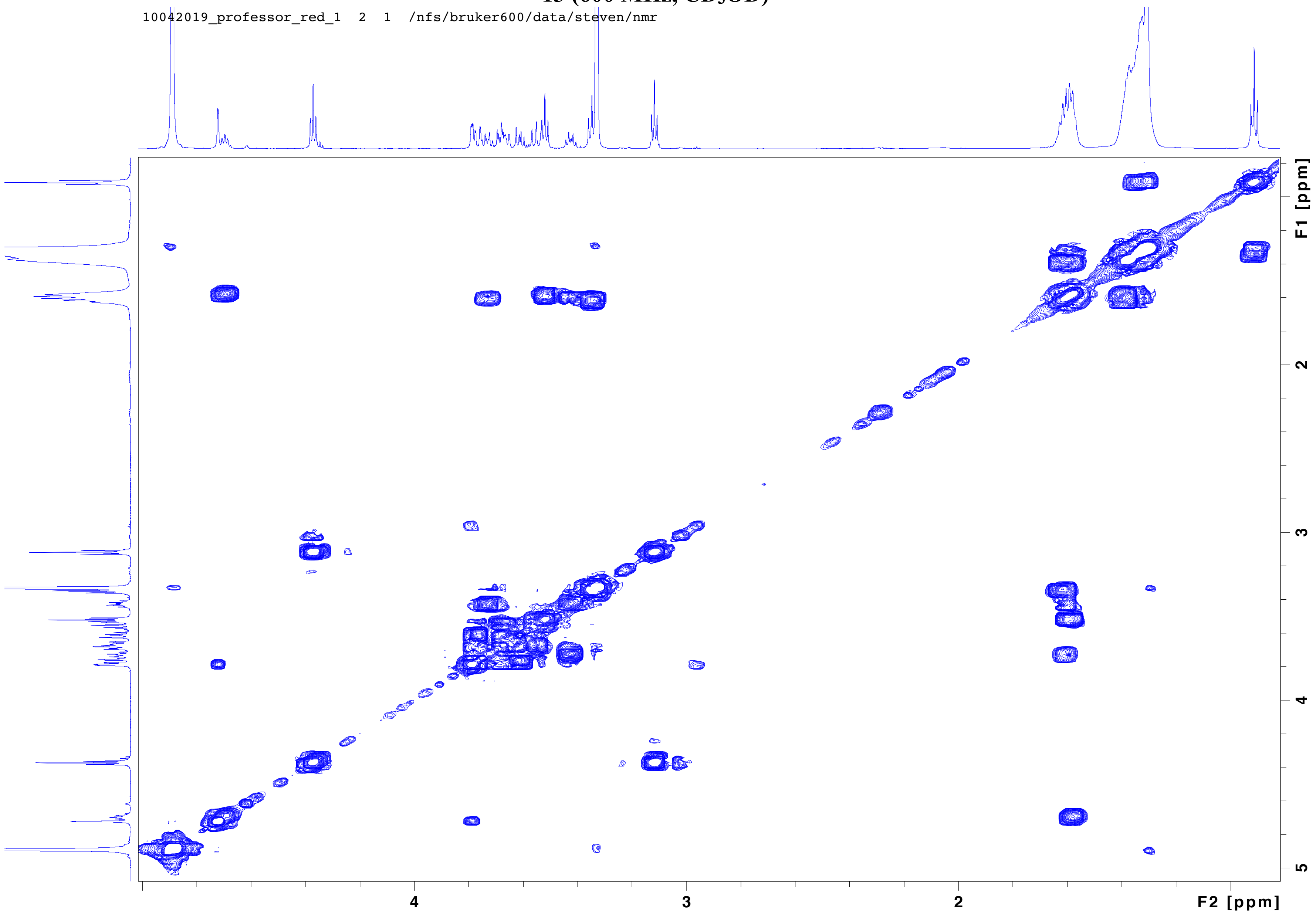

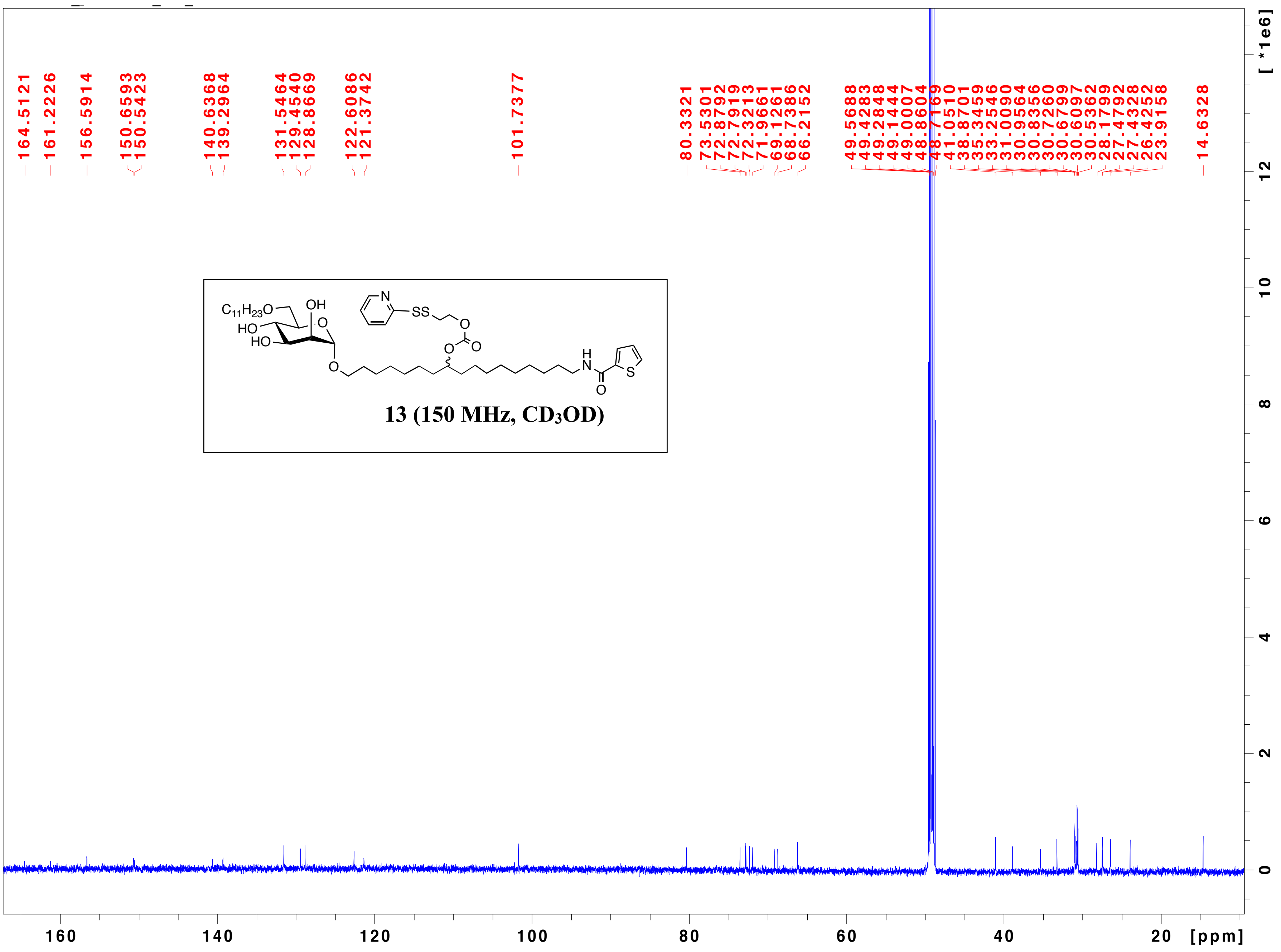
164.5121
161.2226
156.5914
150.6593
150.5423
140.6368
139.2964
131.5464
129.4540
128.8669
122.6086
121.3742
101.7377
80.3321
73.5301
72.8792
72.7919
72.3213
71.9661
69.1261
68.7386
66.2152
49.5688
49.4283
49.2848
49.1444
49.0007
48.8604
48.7169
41.0510
38.8701
35.3459
33.2546
31.0090
30.9564
30.8356
30.7260
30.6797
30.6097
30.5362
28.1799
27.4792
27.4328
26.4252
23.9158
14.6328
[*1e6]
$C_{11}H_{23}O$
OH
HO
HO
O
N
SS
O
O
O
O
H
N
S
O
13 (150 MHz, CD_3OD)
[ppm]

13 (600 MHz, CD_3OD)

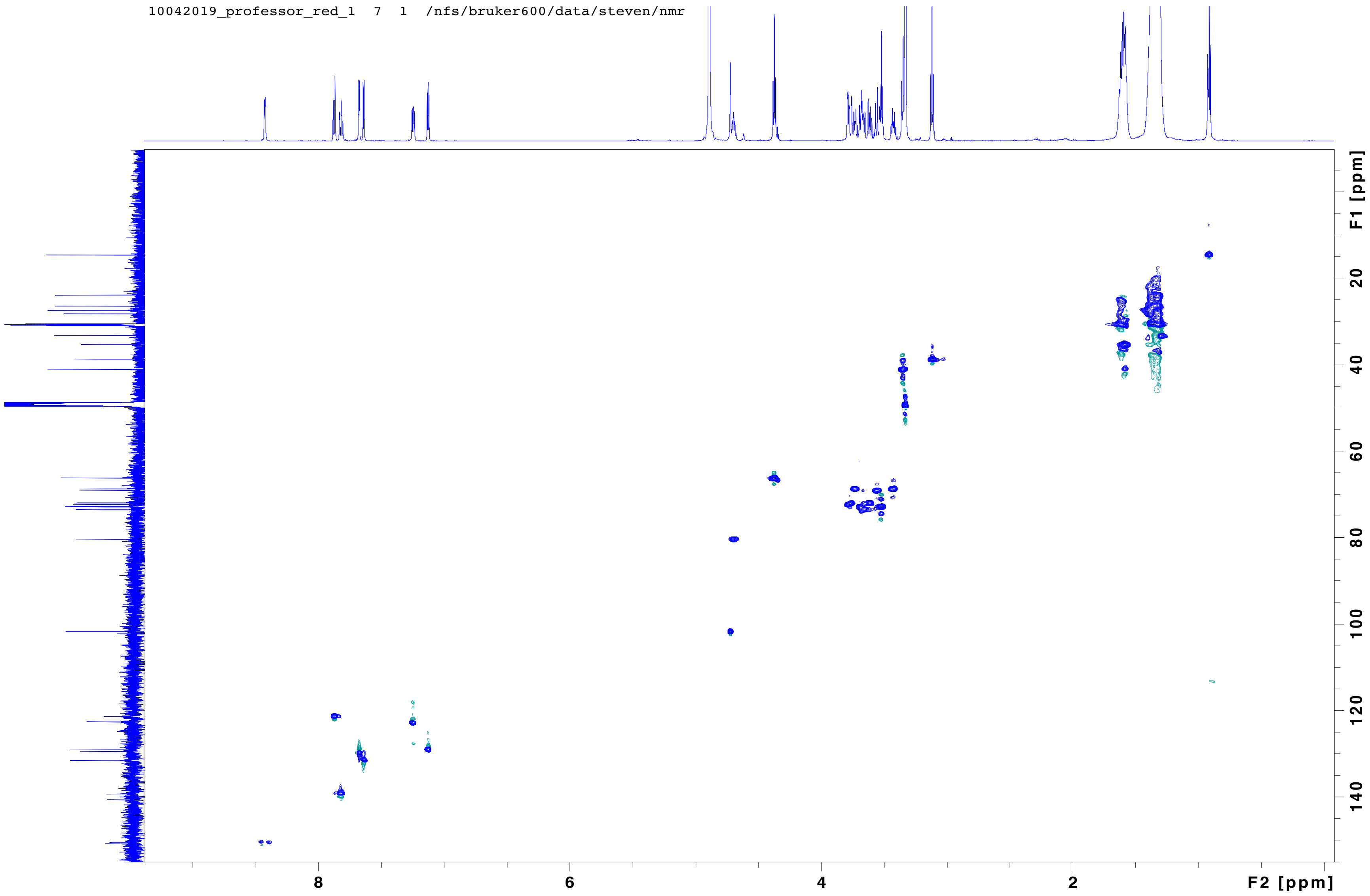

13 (600 MHz, CD$_3$OD)

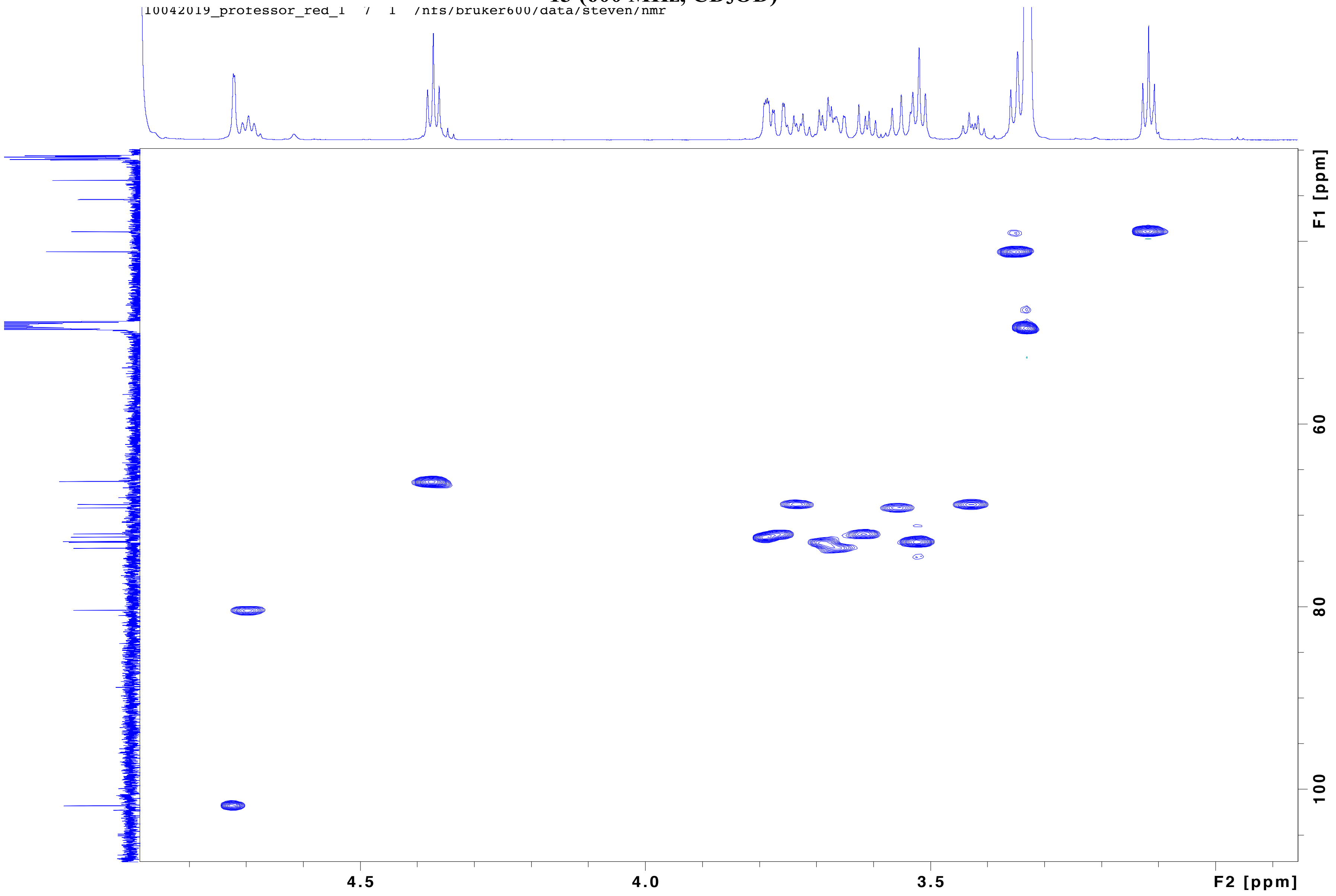

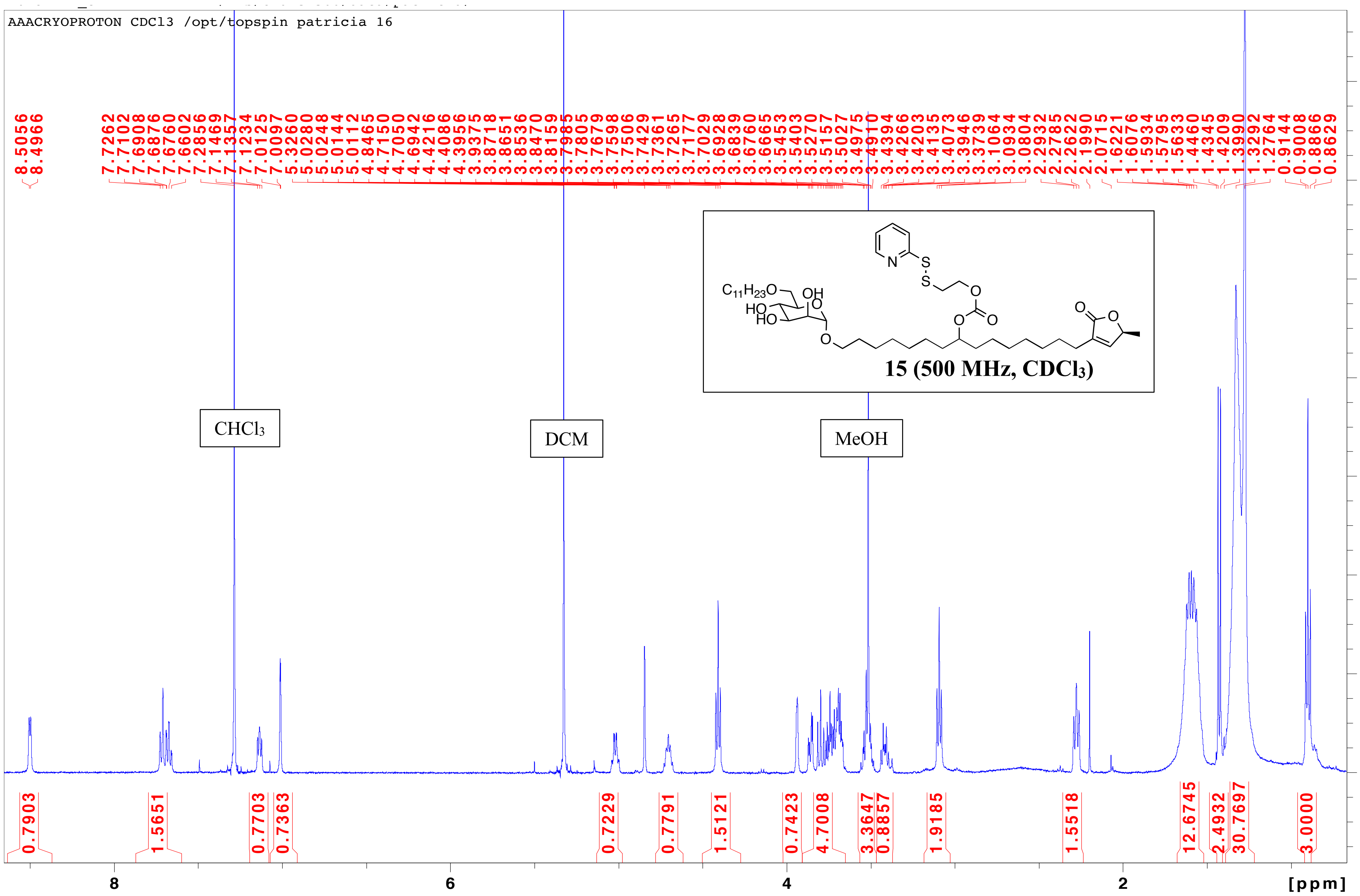
AAACRYOPROTON CDCl3 /opt/topspin patricia 16
CHCl3
DCM
MeOH
15 (500 MHz, CDCl3)
[ppm]

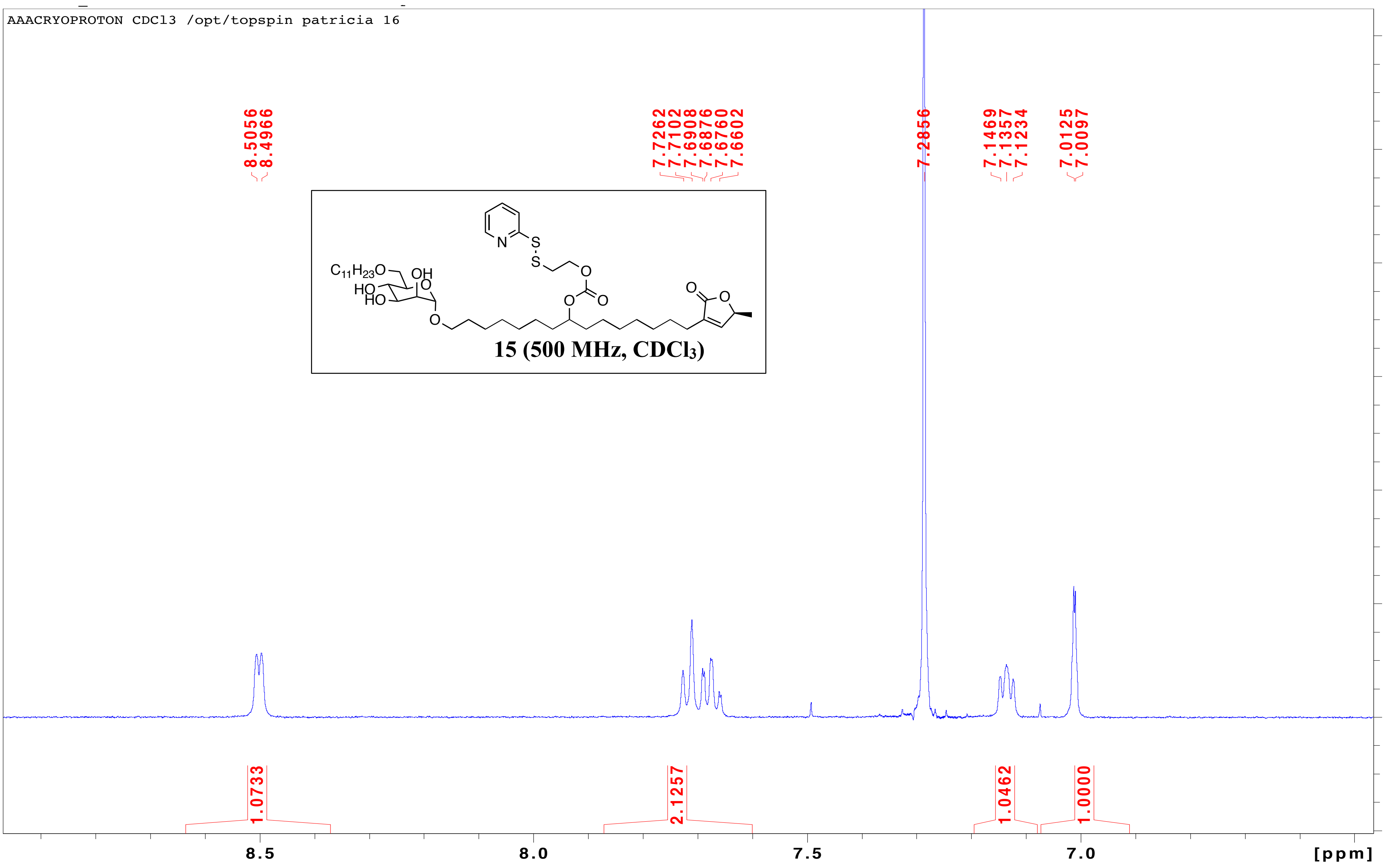
AAACRYOPROTON CDCl3 /opt/topspin patricia 16
8.5056
8.4966
7.7262
7.7102
7.6908
7.6876
7.6760
7.6602
7.2856
7.1469
7.1357
7.1234
7.0125
7.0097
C11H23O
OH
HO
HO
O
O
N
S
S
O
O
O
O
O
O
15 (500 MHz, CDCl3)
1.0733
2.1257
1.0462
1.0000
8.5
8.0
7.5
7.0
[ppm]

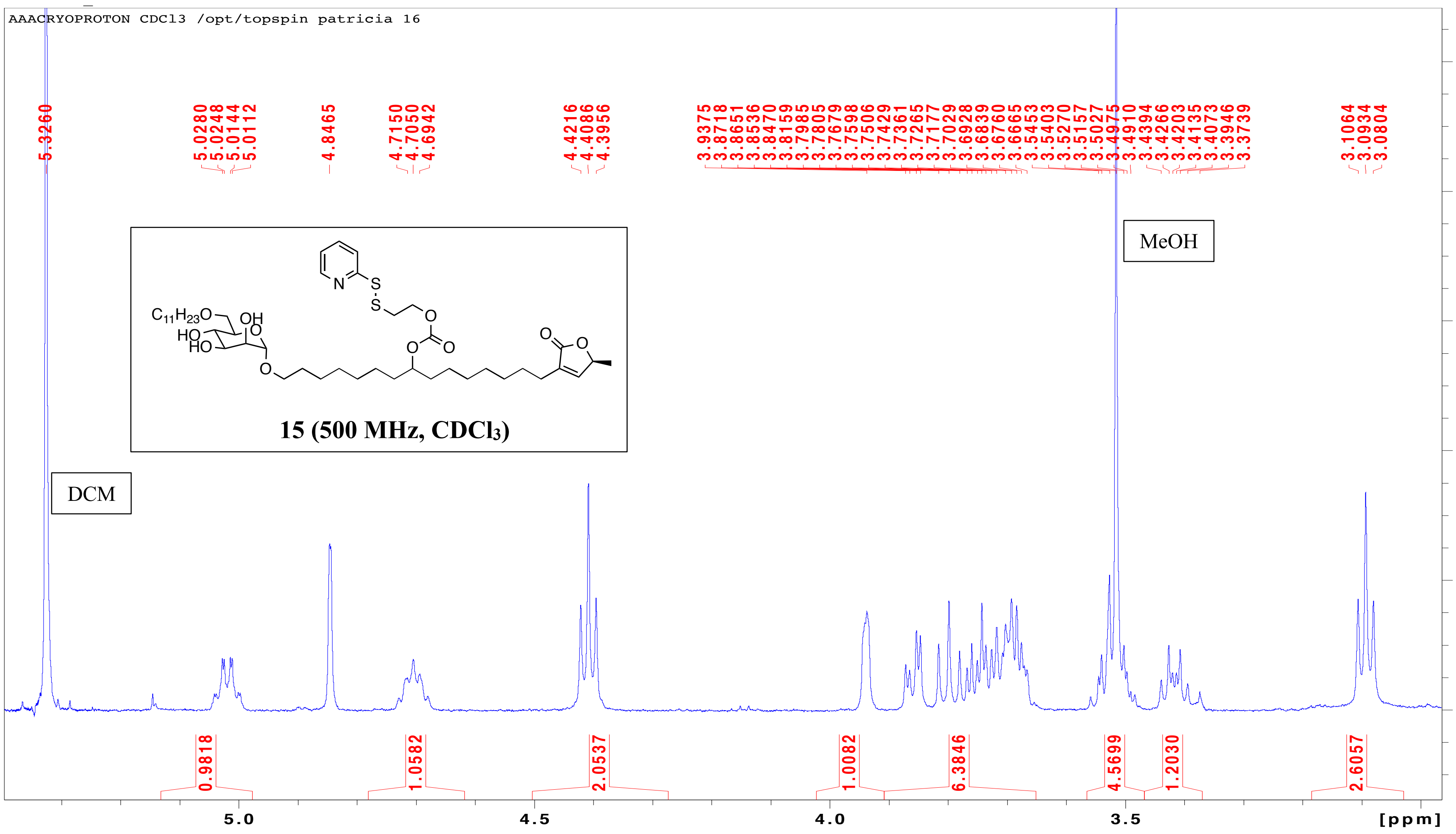
AAACRYOPROTON CDCl3 /opt/topspin patricia 16
5.3260
5.0280
5.0248
5.0144
5.0112
4.8465
4.7150
4.7050
4.6942
4.4216
4.4086
4.3956
3.9375
3.8718
3.8651
3.8536
3.8470
3.8159
3.7985
3.7805
3.7679
3.7598
3.7506
3.7429
3.7361
3.7265
3.7177
3.7029
3.6928
3.6839
3.6760
3.6665
3.5453
3.5403
3.5270
3.5157
3.5027
3.4975
3.4910
3.4394
3.4266
3.4203
3.4135
3.4073
3.3946
3.3739
3.1064
3.0934
3.0804
MeOH
DCM
15 (500 MHz, CDCl3)
0.9818
1.0582
2.0537
1.0082
6.3846
4.5699
1.2030
2.6057
5.0
4.5
4.0
3.5
[ppm]

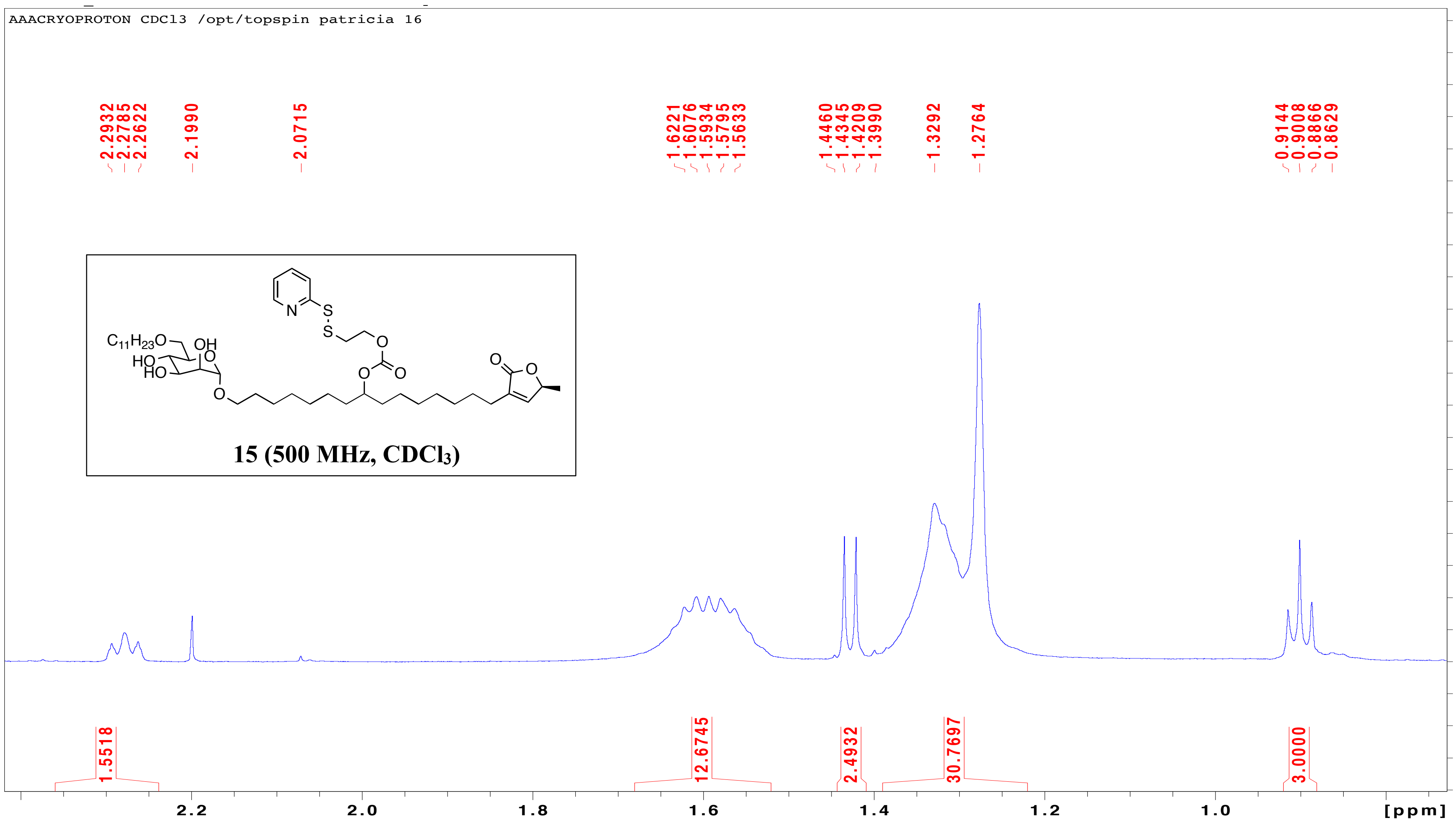
AAACRYOPROTON CDCl3 /opt/topspin patricia 16
2.2932
2.2785
2.2622
2.1990
2.0715
1.6221
1.6076
1.5934
1.5795
1.5633
1.4460
1.4345
1.4209
1.3990
1.3292
1.2764
0.9144
0.9008
0.8866
0.8629
C11H23O
OH
HO
HO
O
O
N
S
S
O
O
O
O
O
O
15 (500 MHz, CDCl3)
1.5518
12.6745
2.4932
30.7697
3.0000
2.2
2.0
1.8
1.6
1.4
1.2
1.0
[ppm]

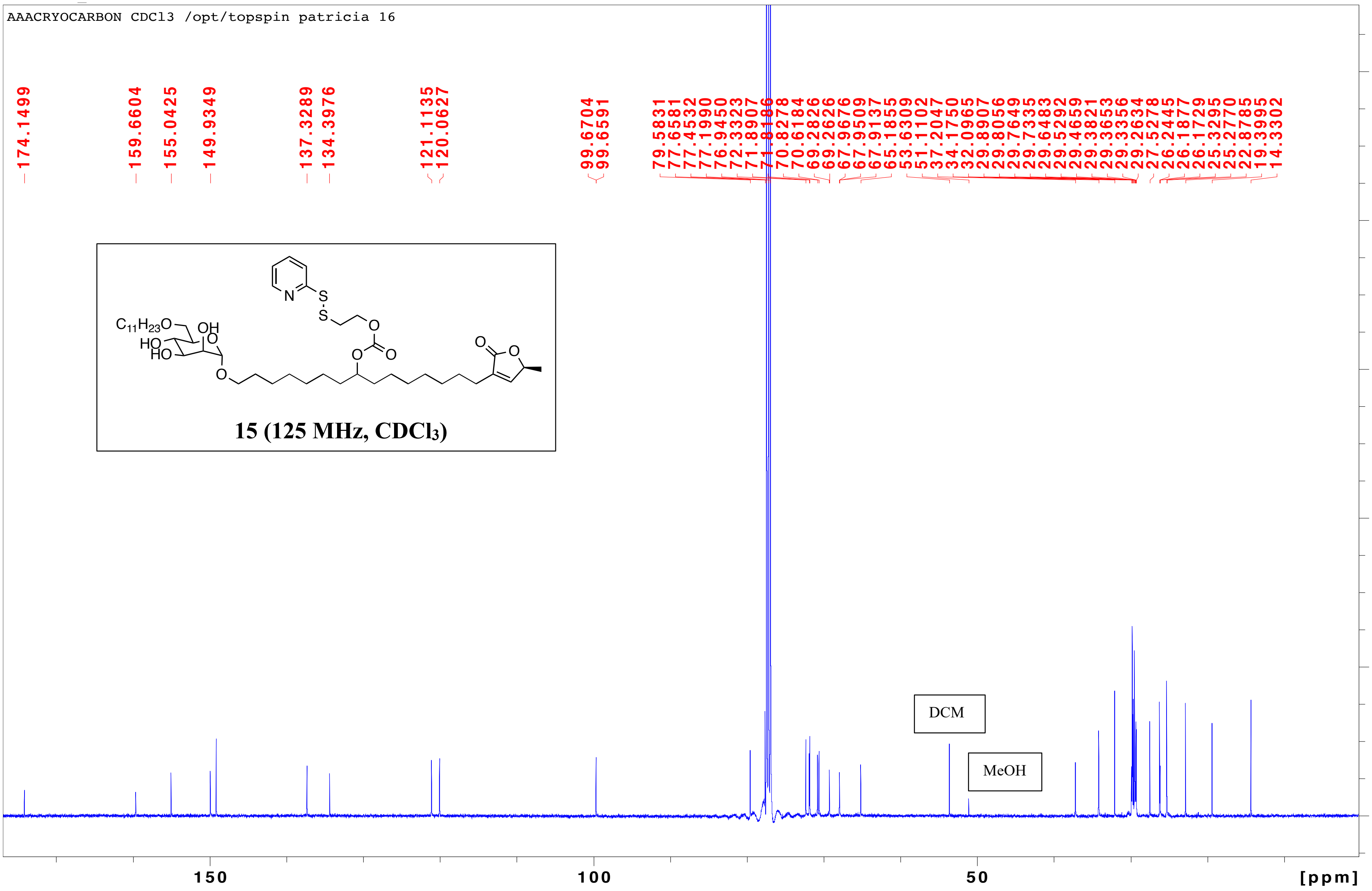
AAACRYOCARBON CDCl3 /opt/topspin patricia 16
174.1499
159.6604
155.0425
149.9349
137.3289
134.3976
121.1135
120.0627
99.6704
99.6591
79.5831
77.6581
77.4532
77.1990
76.9450
72.3323
71.8907
71.8186
70.8278
70.6184
69.2826
69.2626
67.9676
67.9509
67.9137
65.1855
53.6309
51.1102
37.2047
34.1750
32.0965
29.8907
29.8056
29.7649
29.7335
29.6483
29.5292
29.4659
29.3821
29.3653
29.3356
29.2634
27.5278
26.2445
26.1877
26.1729
25.3295
25.2770
22.8785
19.3995
14.3302
15 (125 MHz, CDCl3)
DCM
MeOH
150
100
50
[ppm]

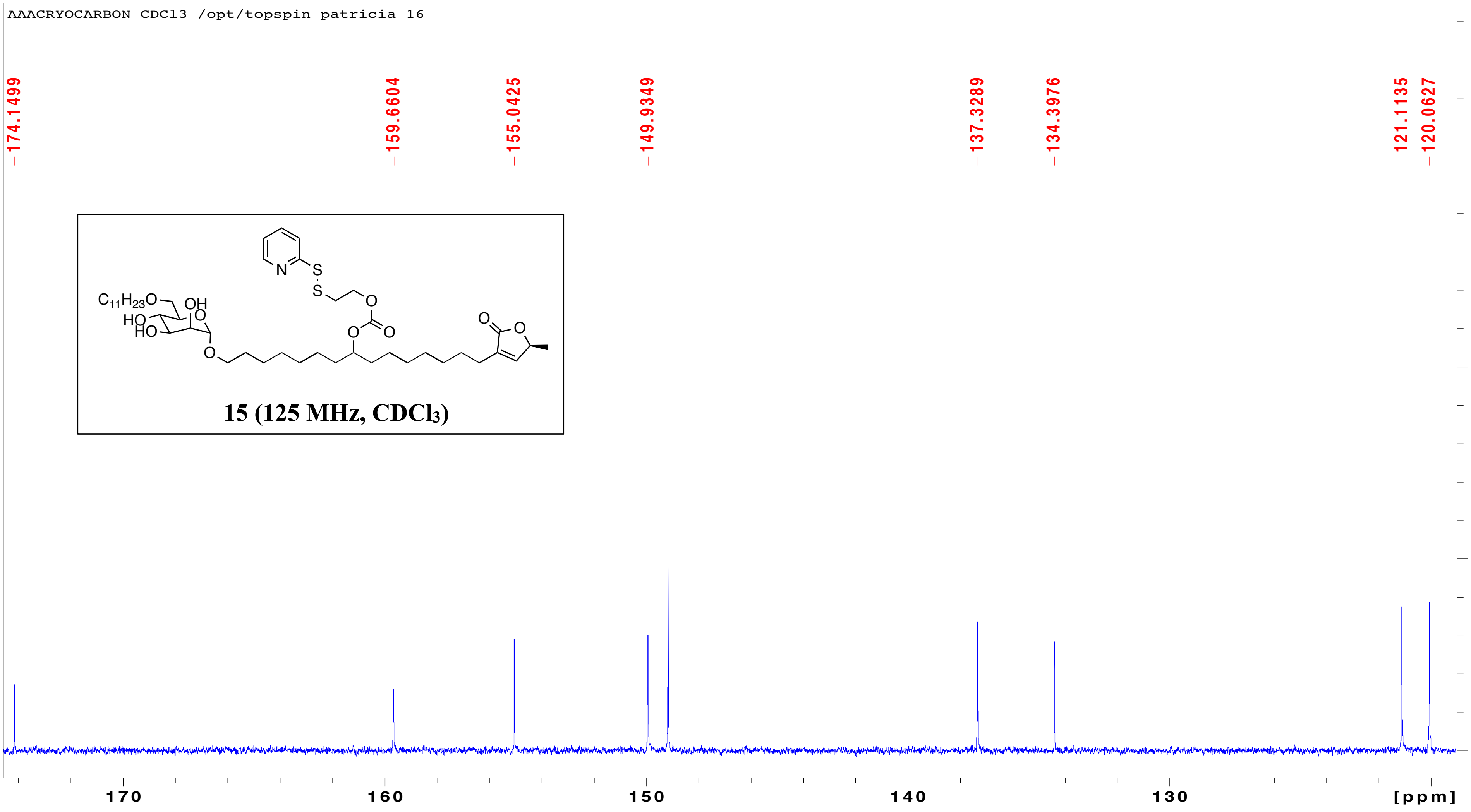
AAACRYOCARBON CDCl3 /opt/topspin patricia 16
174.1499
159.6604
155.0425
149.9349
137.3289
134.3976
121.1135
120.0627
15 (125 MHz, CDCl3)
170
160
150
140
130
[ppm]

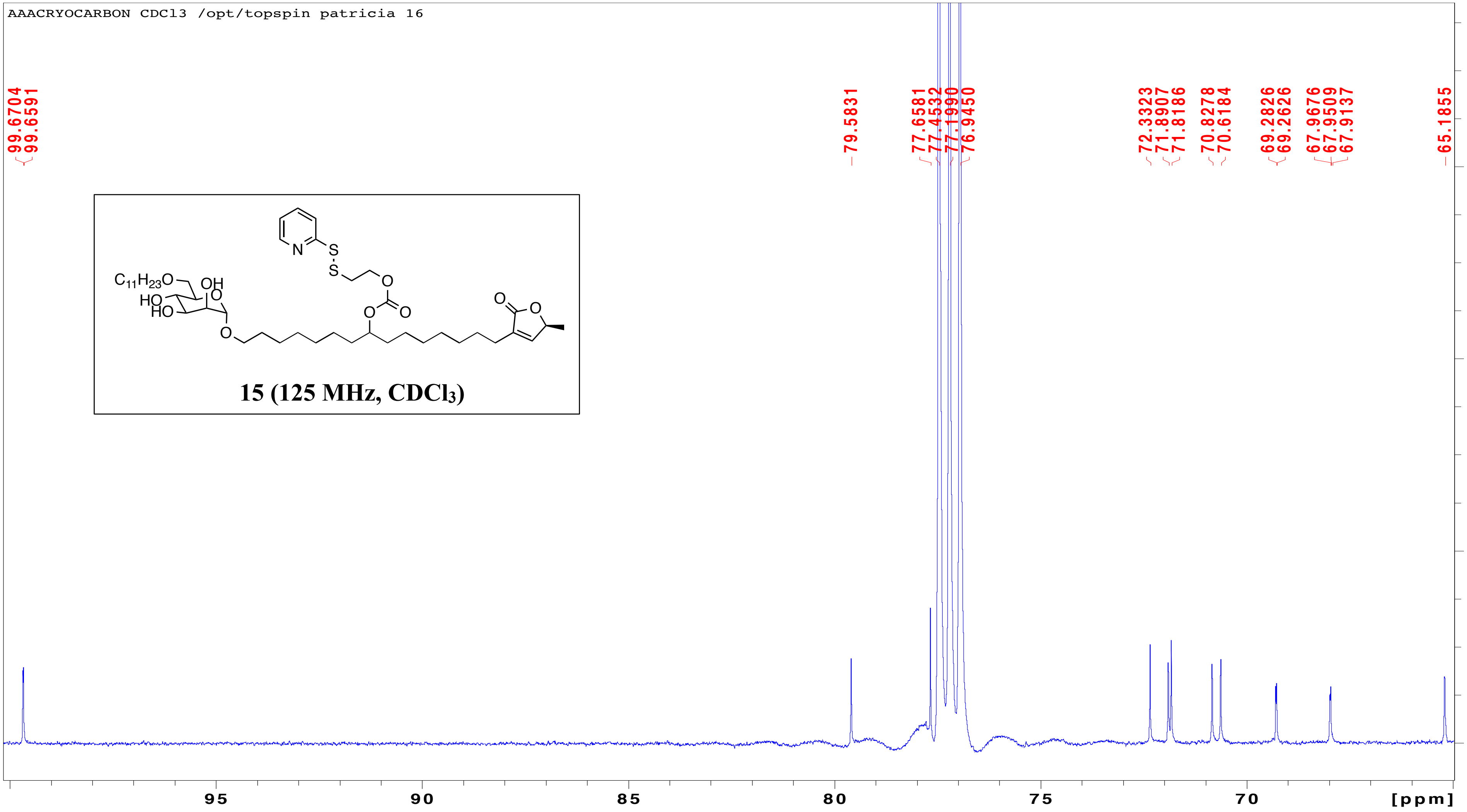
AAACRYOCARBON CDCl3 /opt/topspin patricia 16
99.6704
99.6591
79.5831
77.6581
77.4532
77.1990
76.9450
72.3323
71.8907
71.8186
70.8278
70.6184
69.2826
69.2626
67.9676
67.9509
67.9137
65.1855
15 (125 MHz, CDCl3)
95
90
85
80
75
70
[ppm]

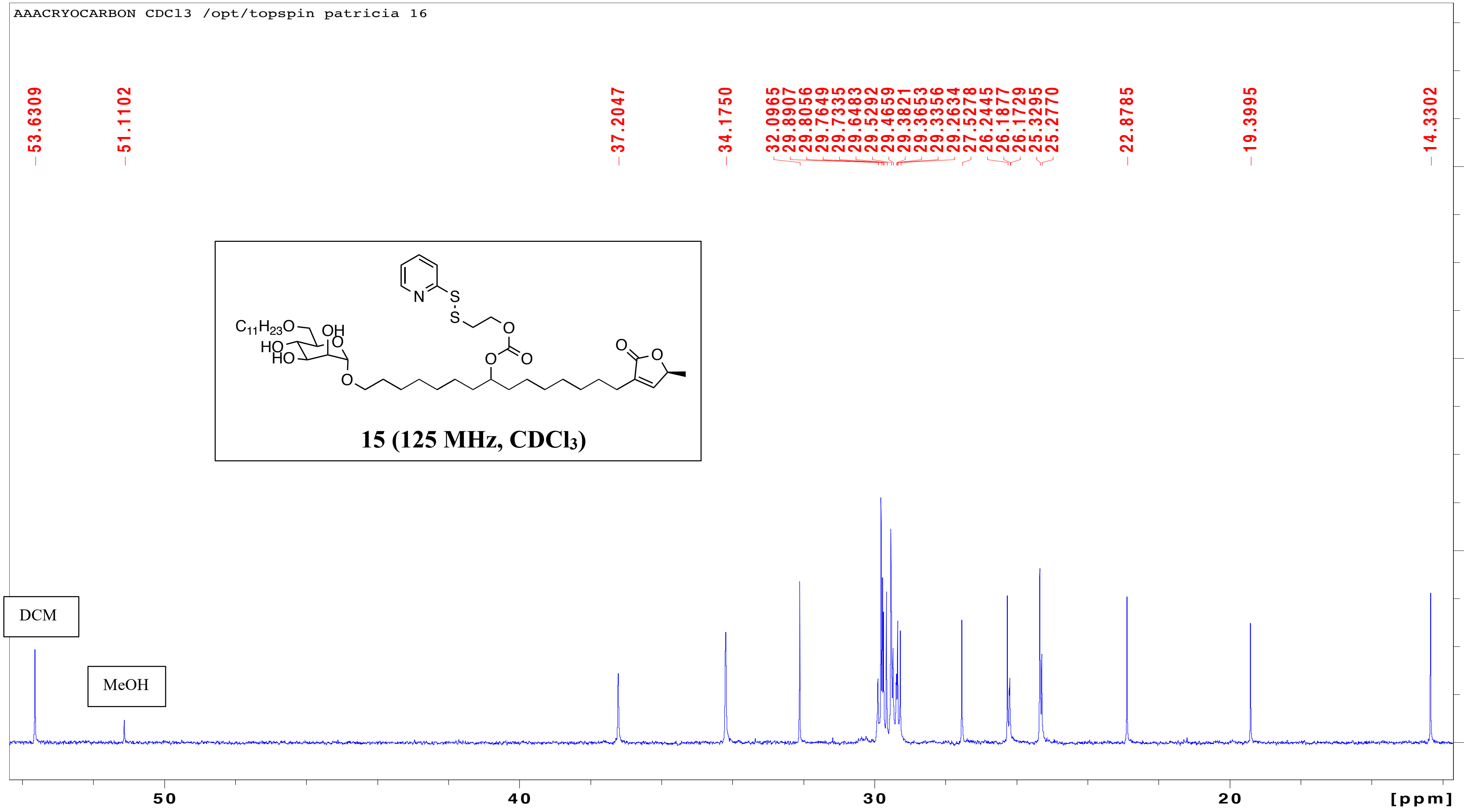
AAACRYOCARBON CDCl3 /opt/topspin patricia 16
53.6309
51.1102
37.2047
34.1750
32.0965
29.8907
29.8056
29.7649
29.7335
29.6483
29.5292
29.4659
29.3821
29.3653
29.3356
29.2634
27.5278
26.2445
26.1877
26.1729
25.3295
25.2770
22.8785
19.3995
14.3302
15 (125 MHz, CDCl3)
DCM
MeOH
50
40
30
20
[ppm]

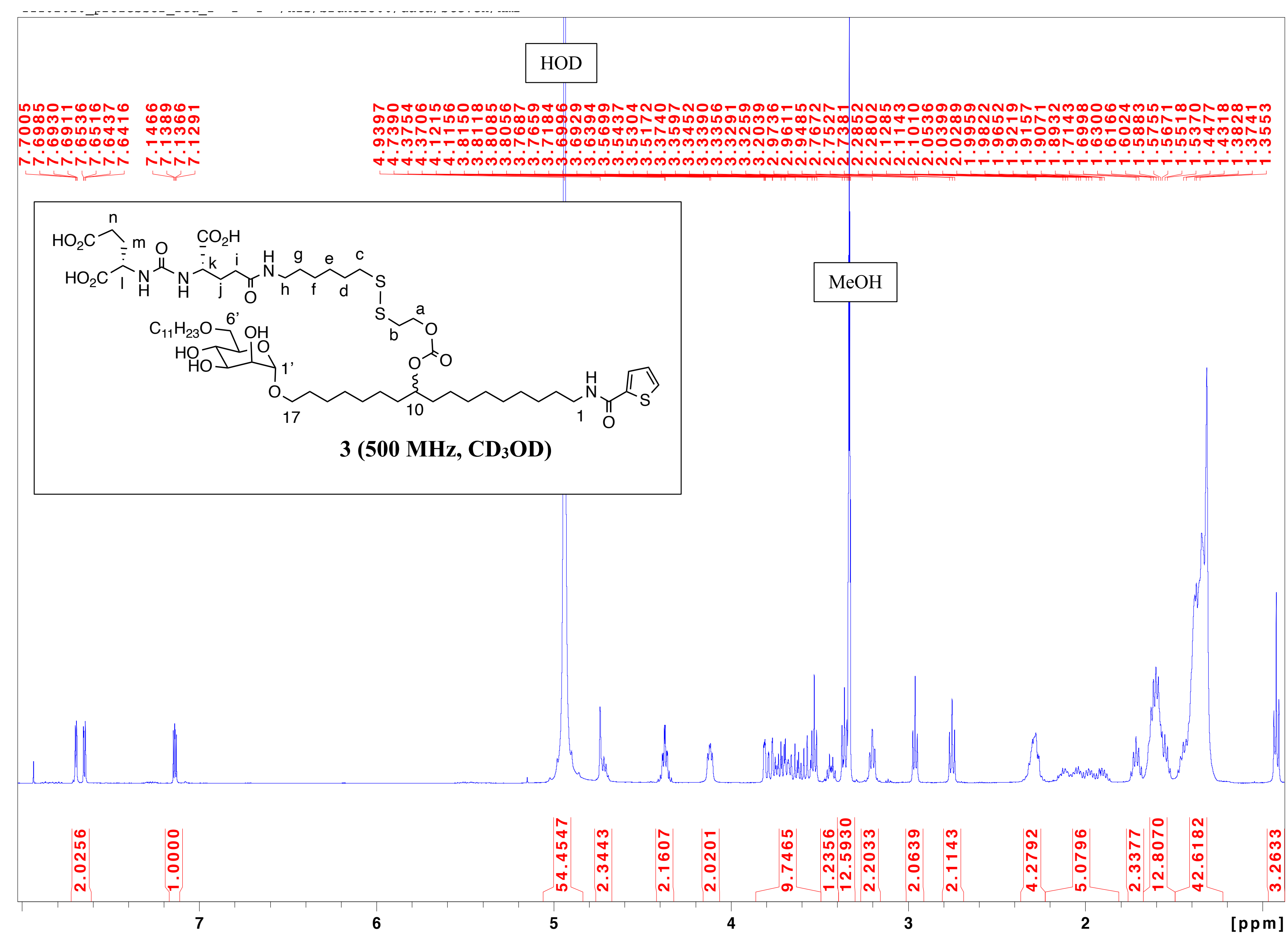
HOD
MeOH
3 (500 MHz, CD3OD)
[ppm]

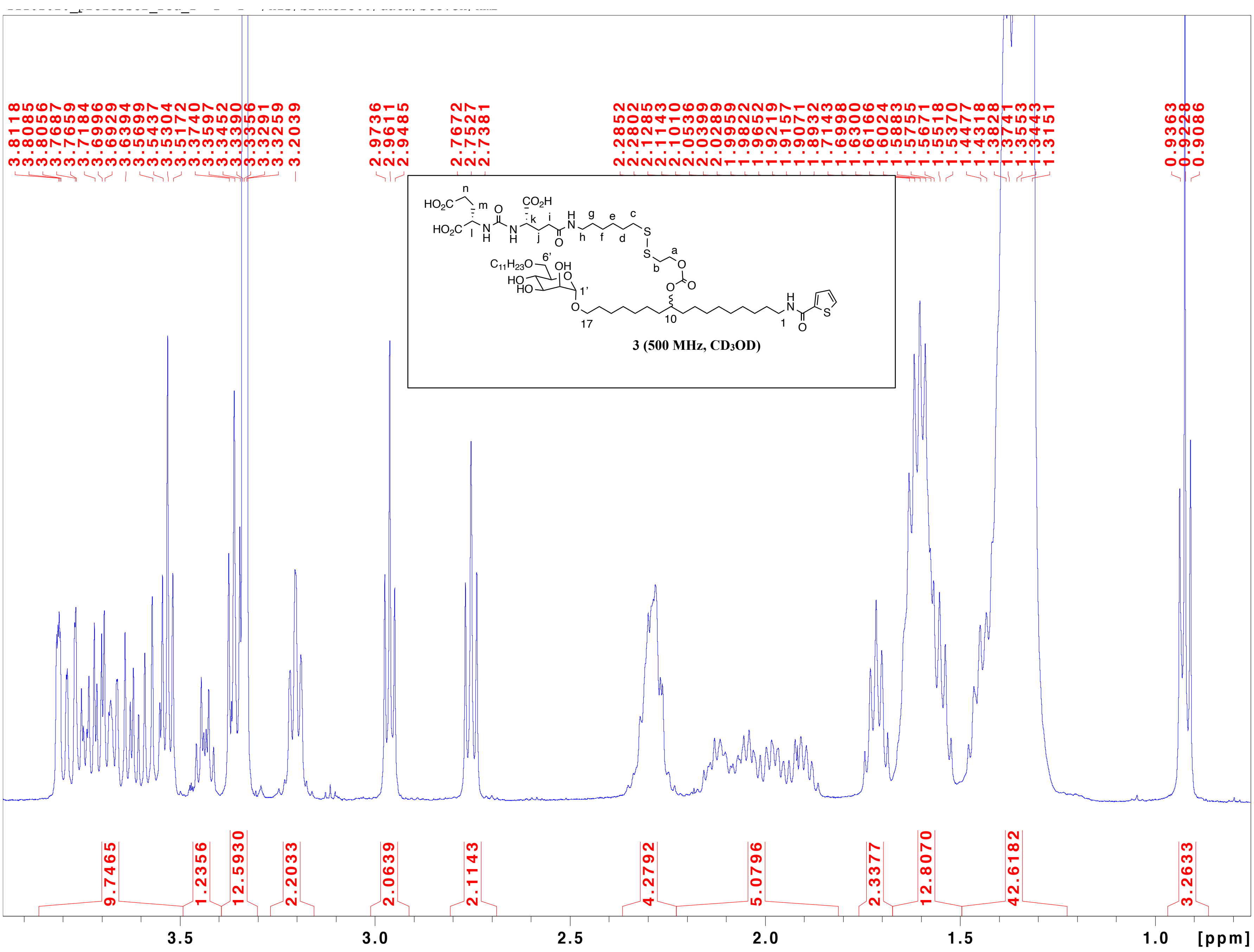
3 (500 MHz, CD3OD)
[ppm]

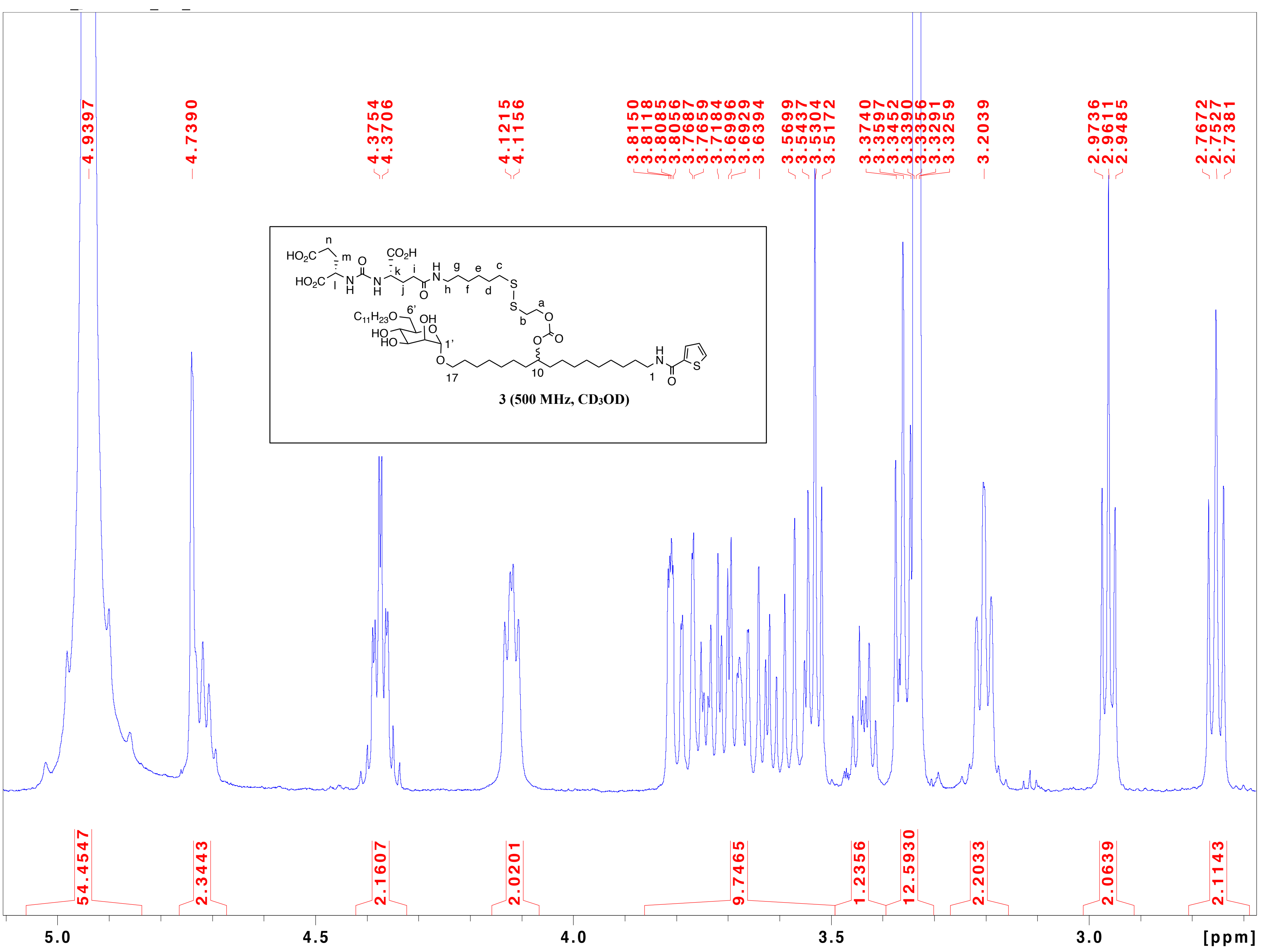
4.9397
4.7390
4.3754
4.3706
4.1215
4.1156
3.8150
3.8118
3.8085
3.8056
3.7687
3.7659
3.7184
3.6996
3.6929
3.6394
3.5699
3.5437
3.5304
3.5172
3.3740
3.3597
3.3452
3.3390
3.3356
3.3291
3.3259
3.2039
2.9736
2.9611
2.9485
2.7672
2.7527
2.7381
3 (500 MHz, CD3OD)
54.4547
2.3443
2.1607
2.0201
9.7465
1.2356
12.5930
2.2033
2.0639
2.1143
5.0
4.5
4.0
3.5
3.0
[ppm]

3 (500 MHz, CD$_3$OD)

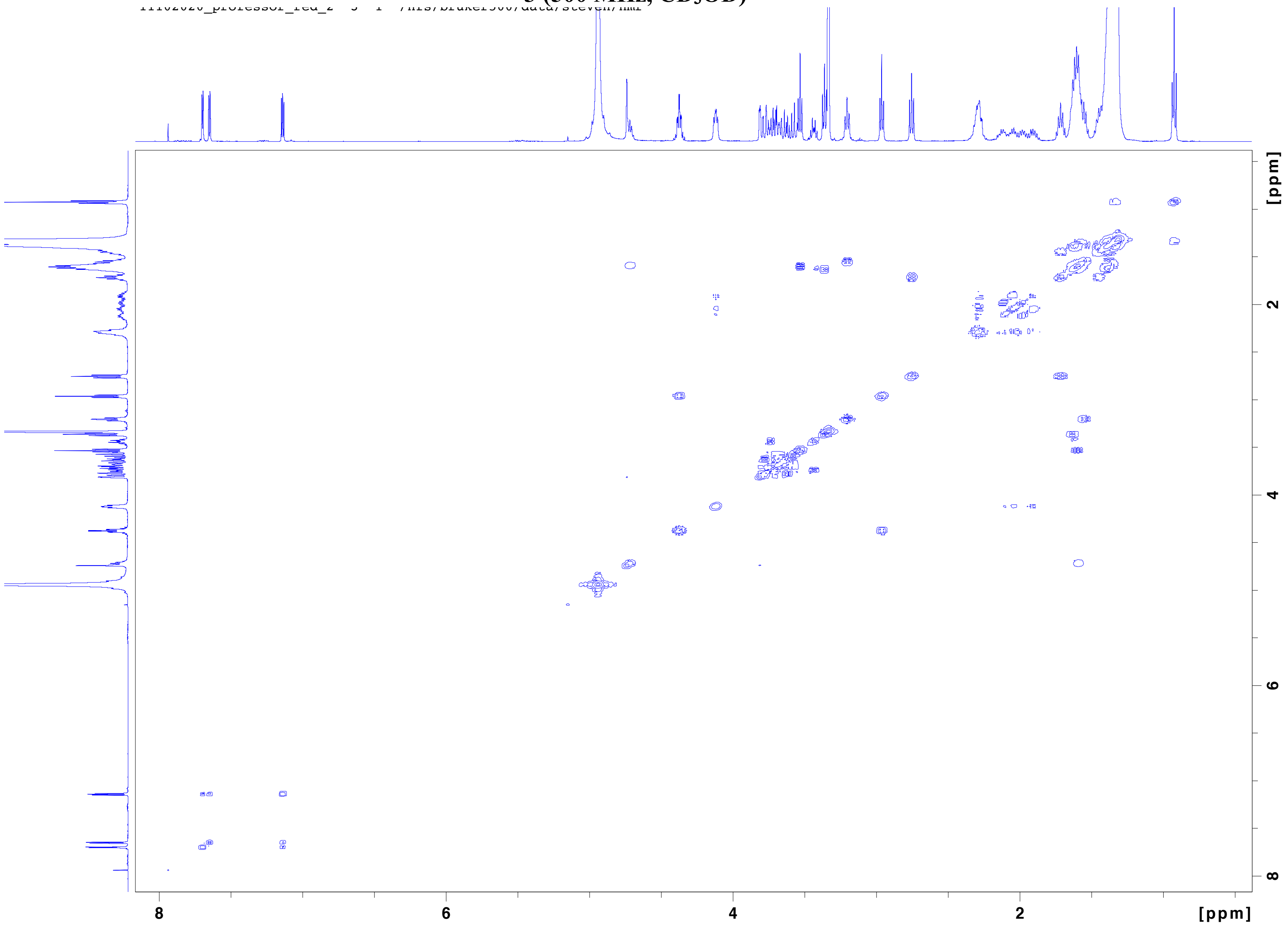

3 (125 MHz, CD_3OD)

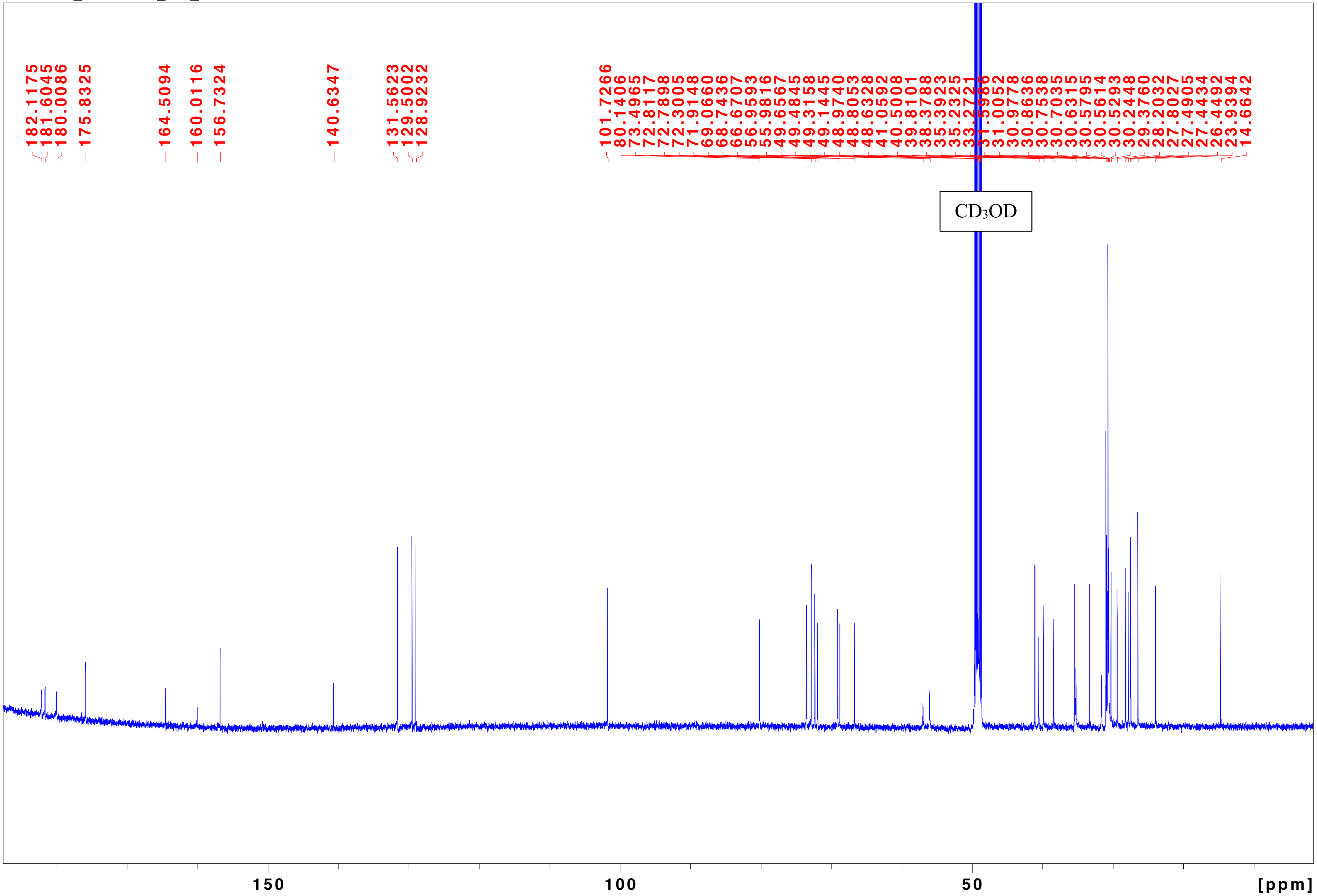

3 (125 MHz, CD_3OD)

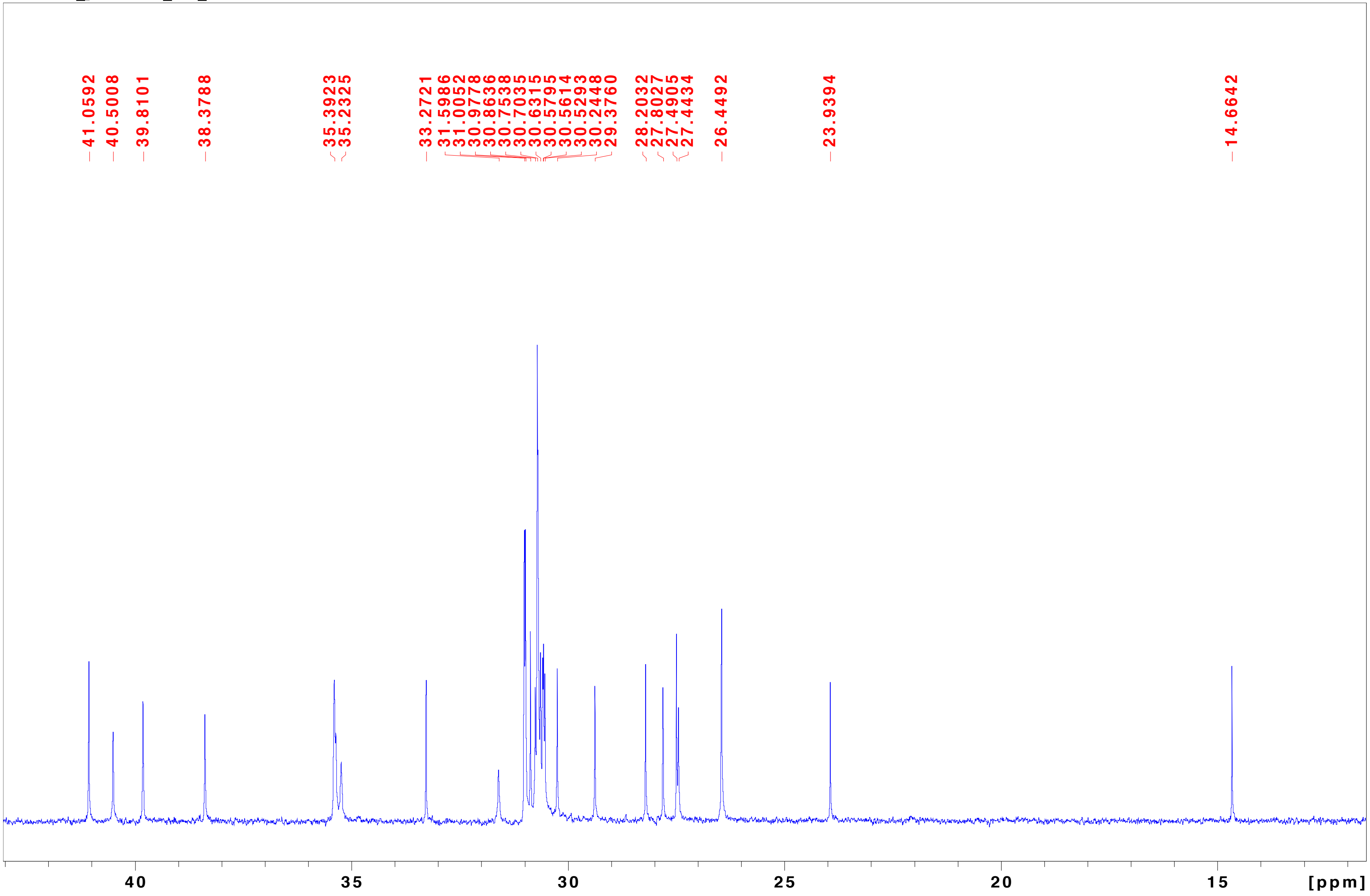

3 (125 MHz, CD_3OD)

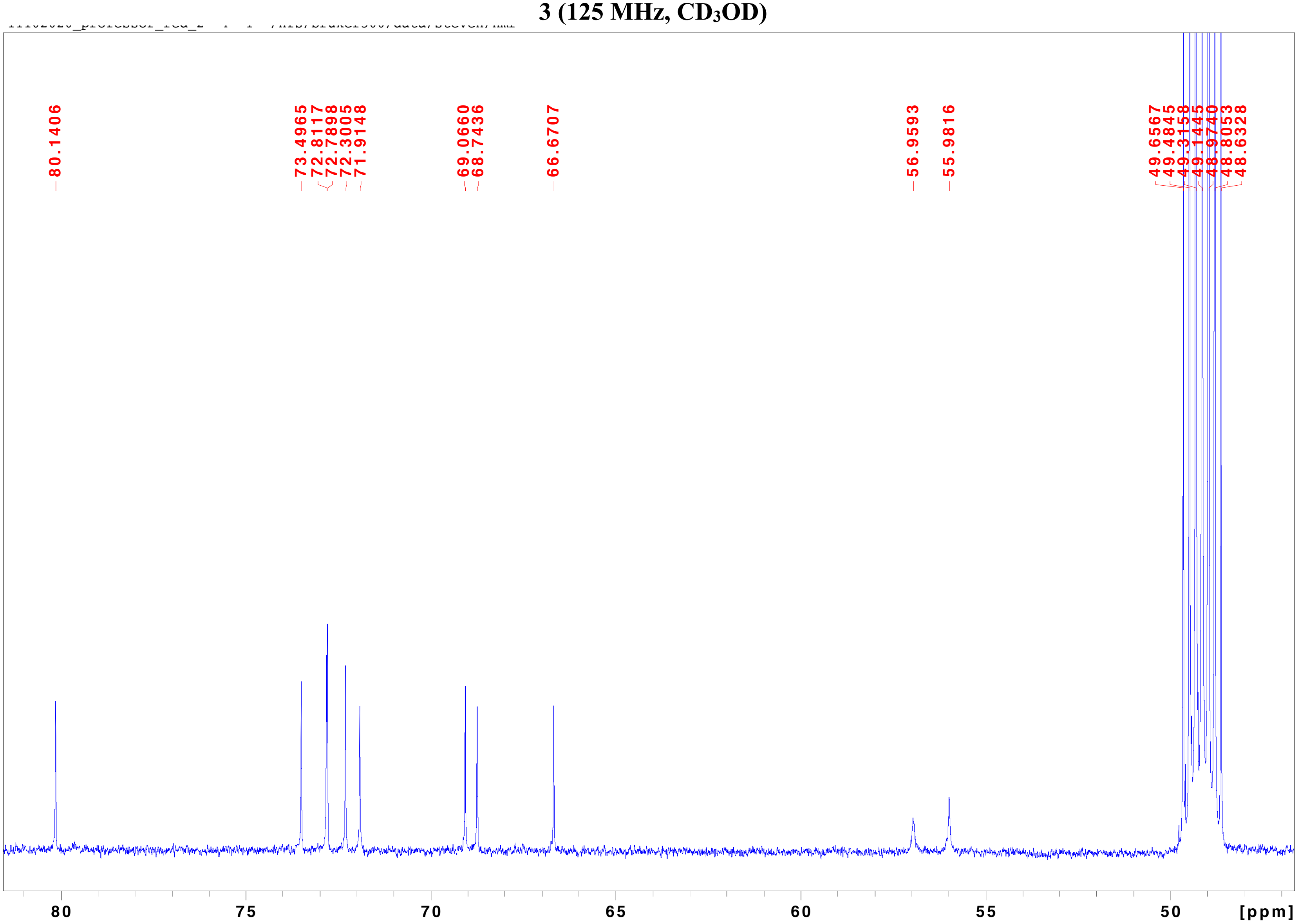

3 (125 MHz, CD_3OD)

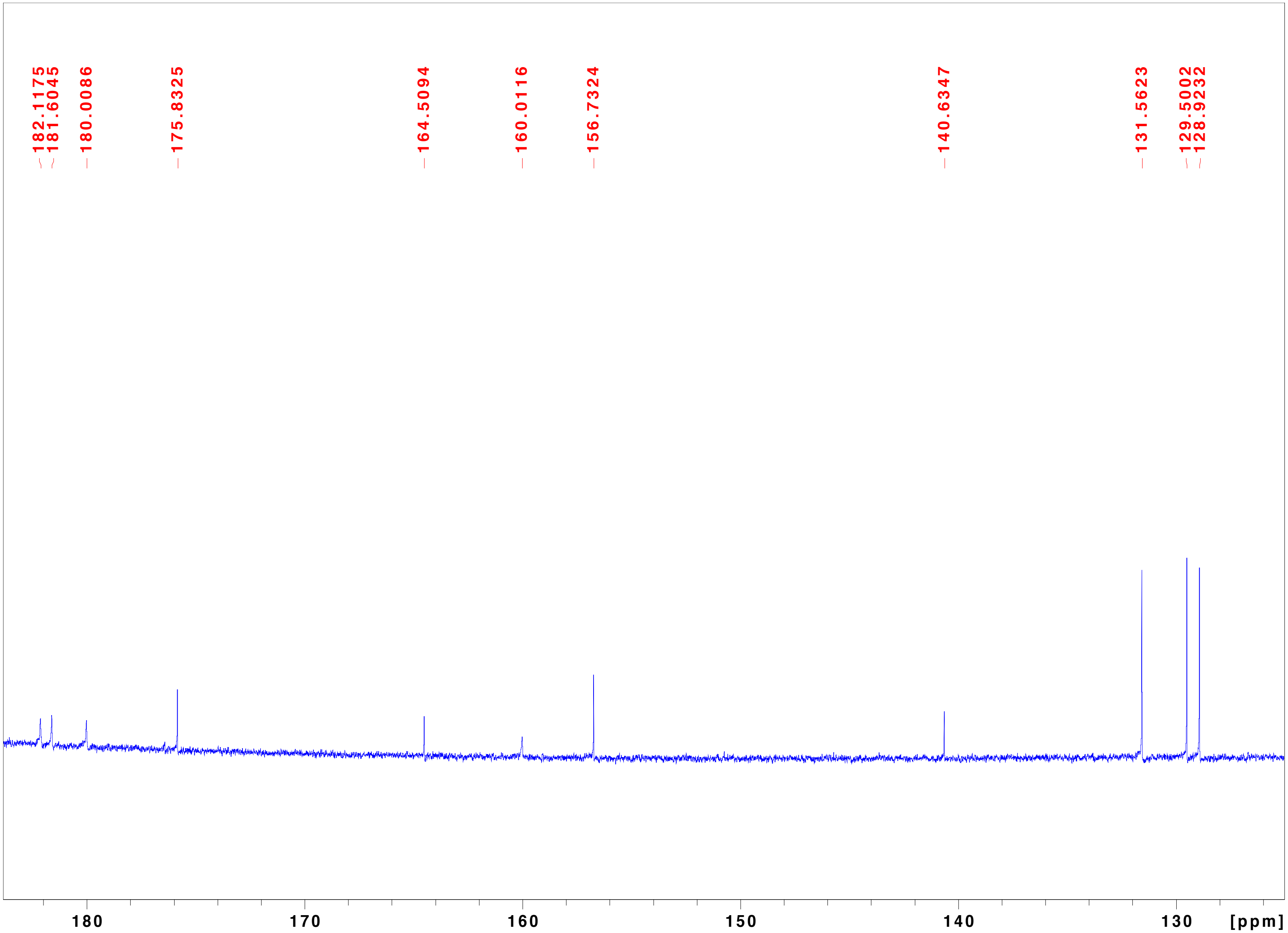

3 (500 MHz, CD3OD)

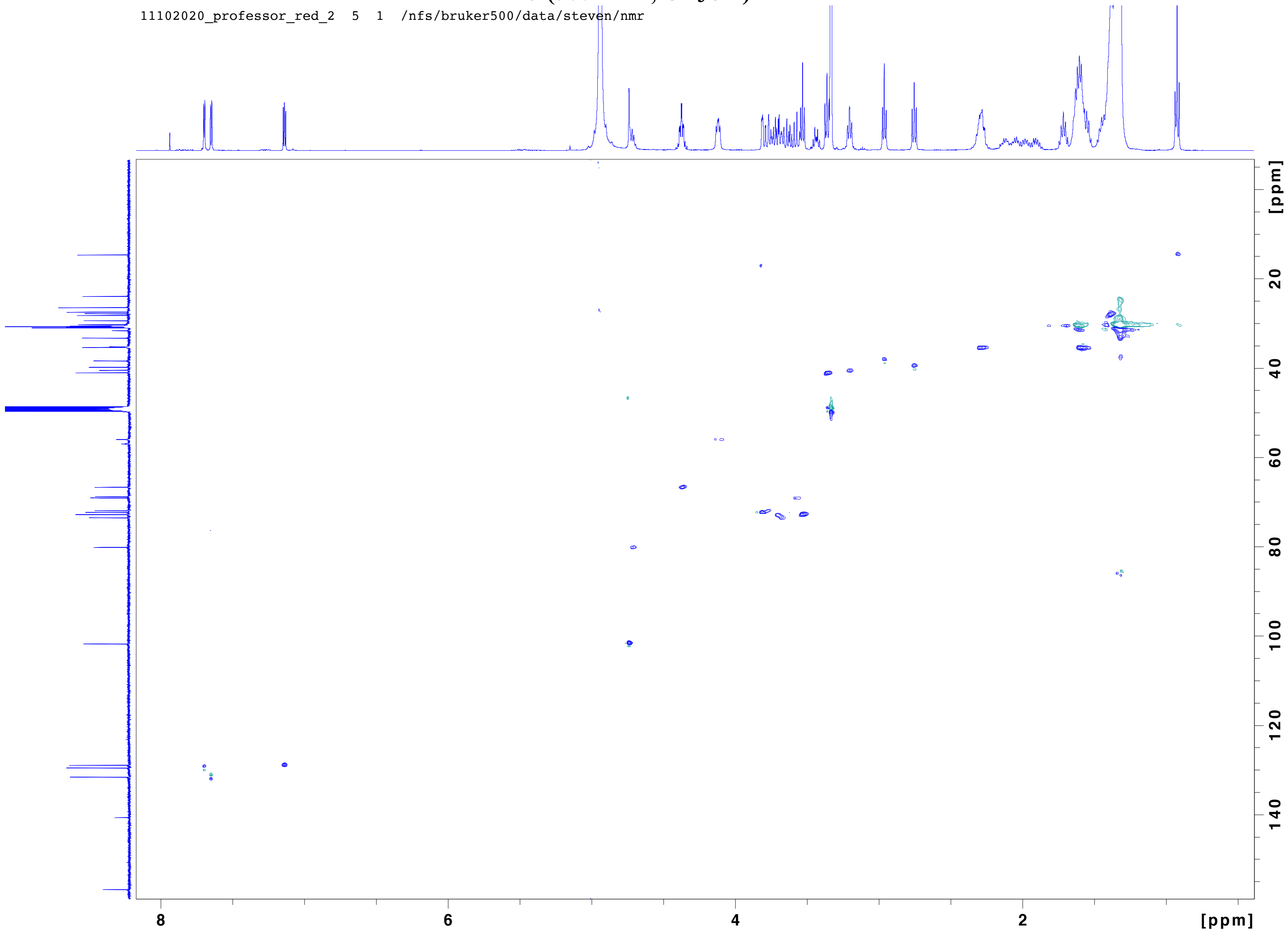

3 (500 MHz, CD_3OD)

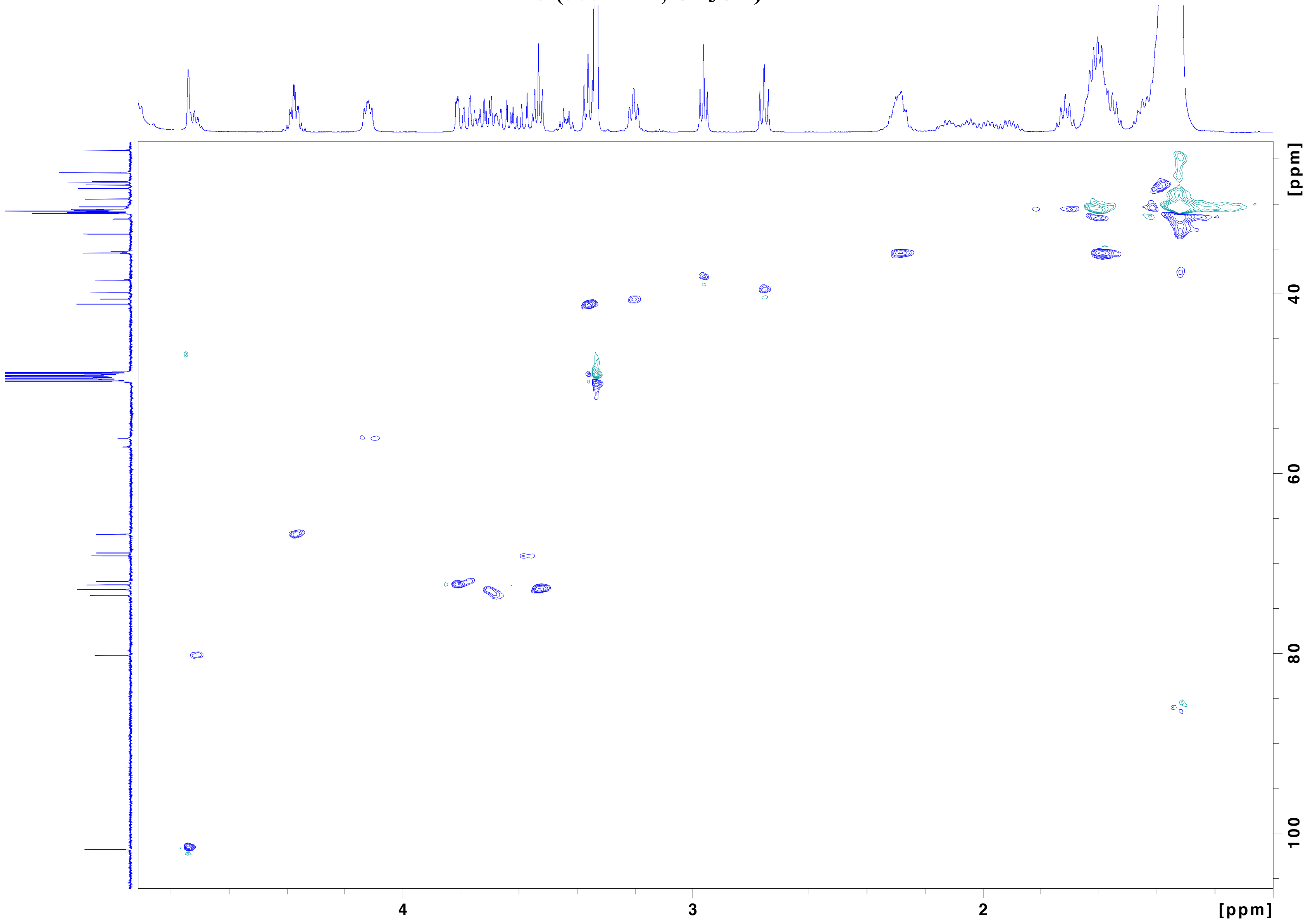

3 (500 MHz, CD_3OD)

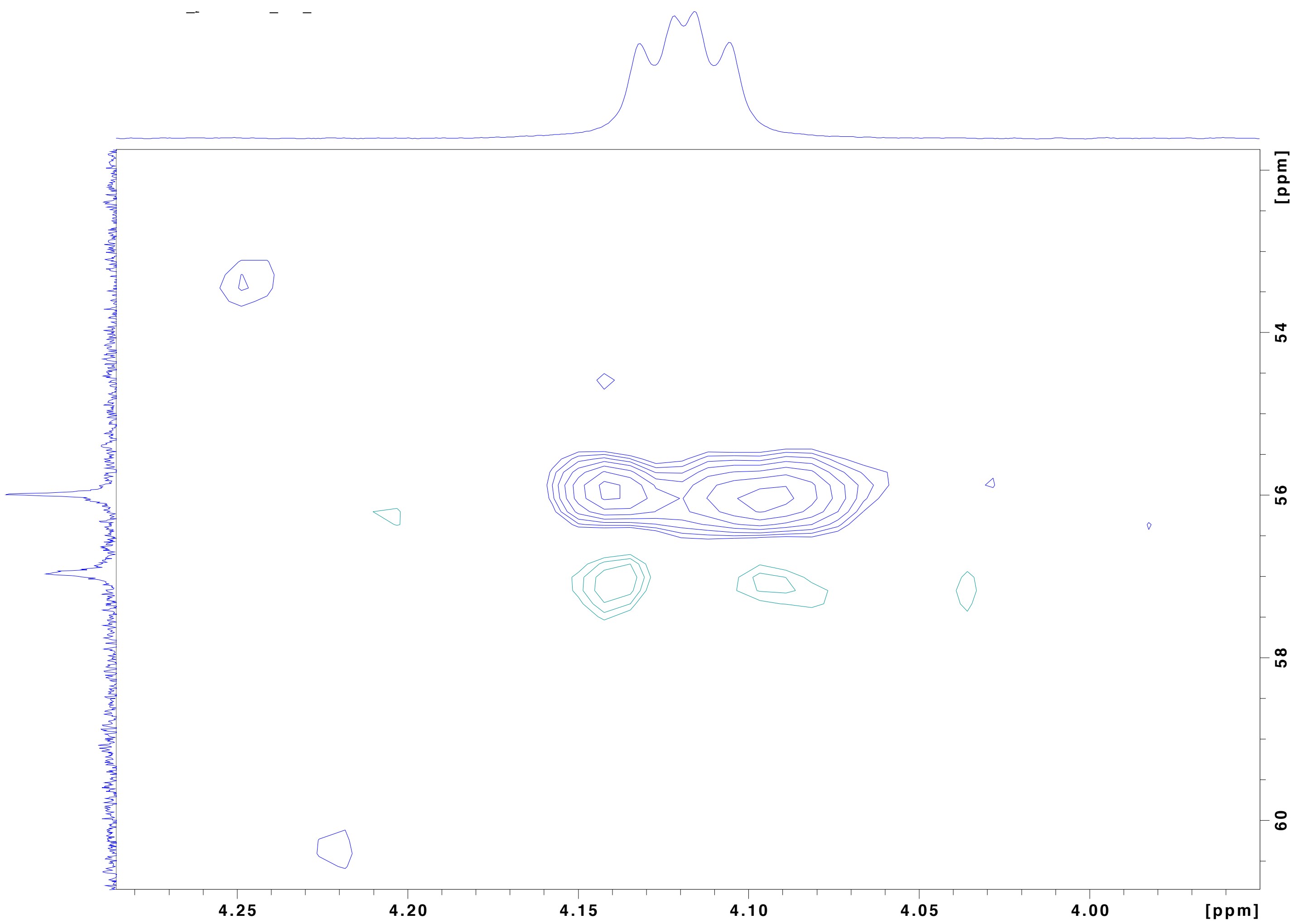

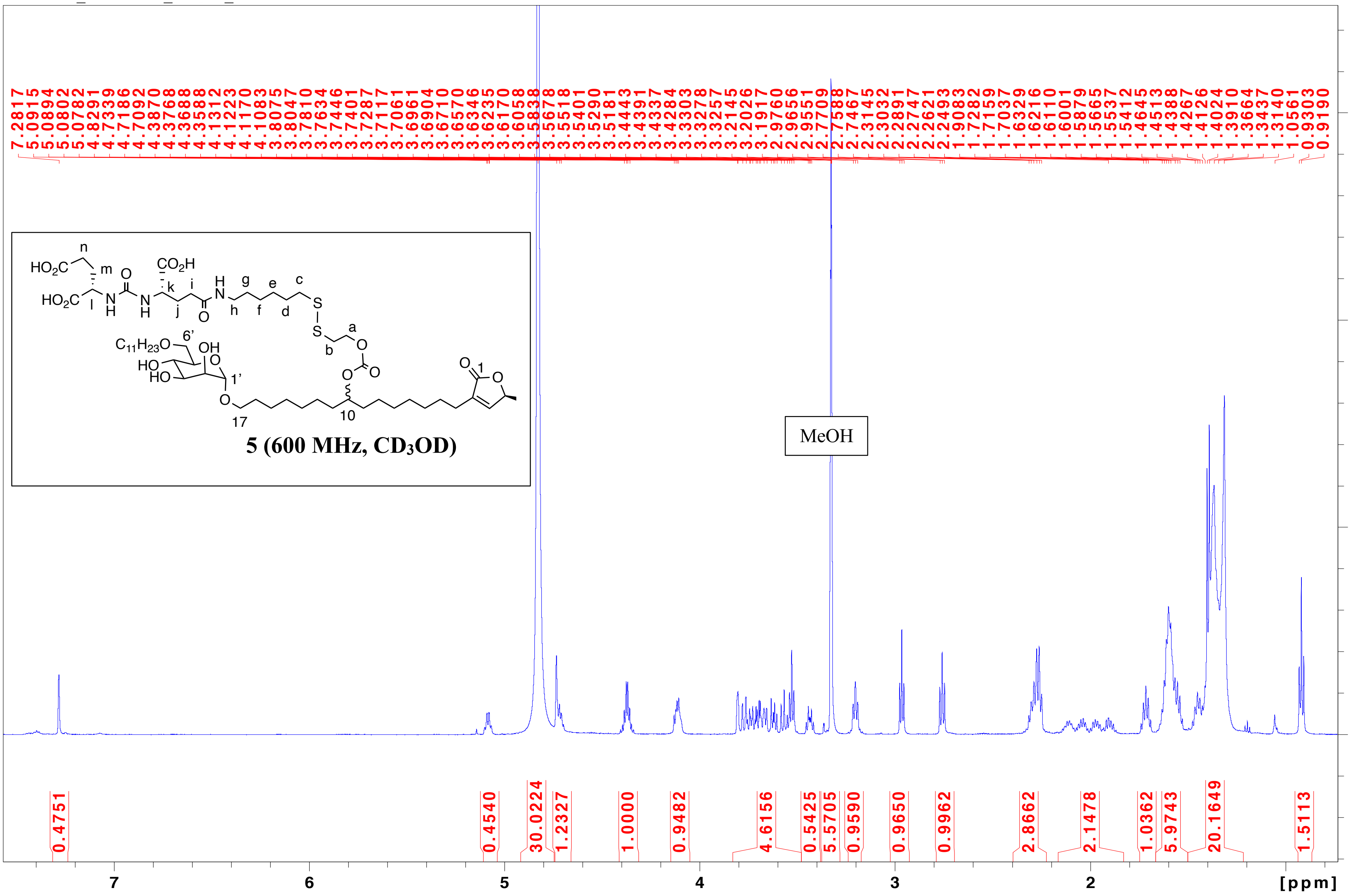
7.2817 5.0915 5.0894 5.0802 5.0782 4.8291 4.7339 4.7186 4.7092 4.3870 4.3768 4.3688 4.3588 4.1312 4.1223 4.1170 4.1083 4.1075 3.8047 3.7810 3.7634 3.7446 3.7401 3.7287 3.7117 3.7061 3.6961 3.6904 3.6710 3.6570 3.6346 3.6235 3.6170 3.6058 3.5838 3.5678 3.5518 3.5401 3.5290 3.5181 3.4443 3.4391 3.4337 3.4284 3.3078 3.3257 3.2145 3.2026 3.1917 2.9760 2.9656 2.9551 2.7709 2.7588 2.7467 2.3145 2.3032 2.2891 2.2747 2.2621 2.2493 1.9083 1.7282 1.7159 1.7037 1.6329 1.6216 1.6101 1.6079 1.5879 1.5665 1.5537 1.5512 1.4645 1.4513 1.4388 1.4267 1.4126 1.4024 1.3910 1.3664 1.3437 1.3140 1.0561 0.9303 0.9190
5 (600 MHz, CD3OD)
MeOH
0.4751 0.4540 30.0224 1.2327 1.0000 0.9482 4.6156 0.5425 5.5705 0.9590 0.9650 0.9962 2.8662 2.1478 1.0362 5.9743 20.1649 1.5113
7 6 5 4 3 2 [ppm]

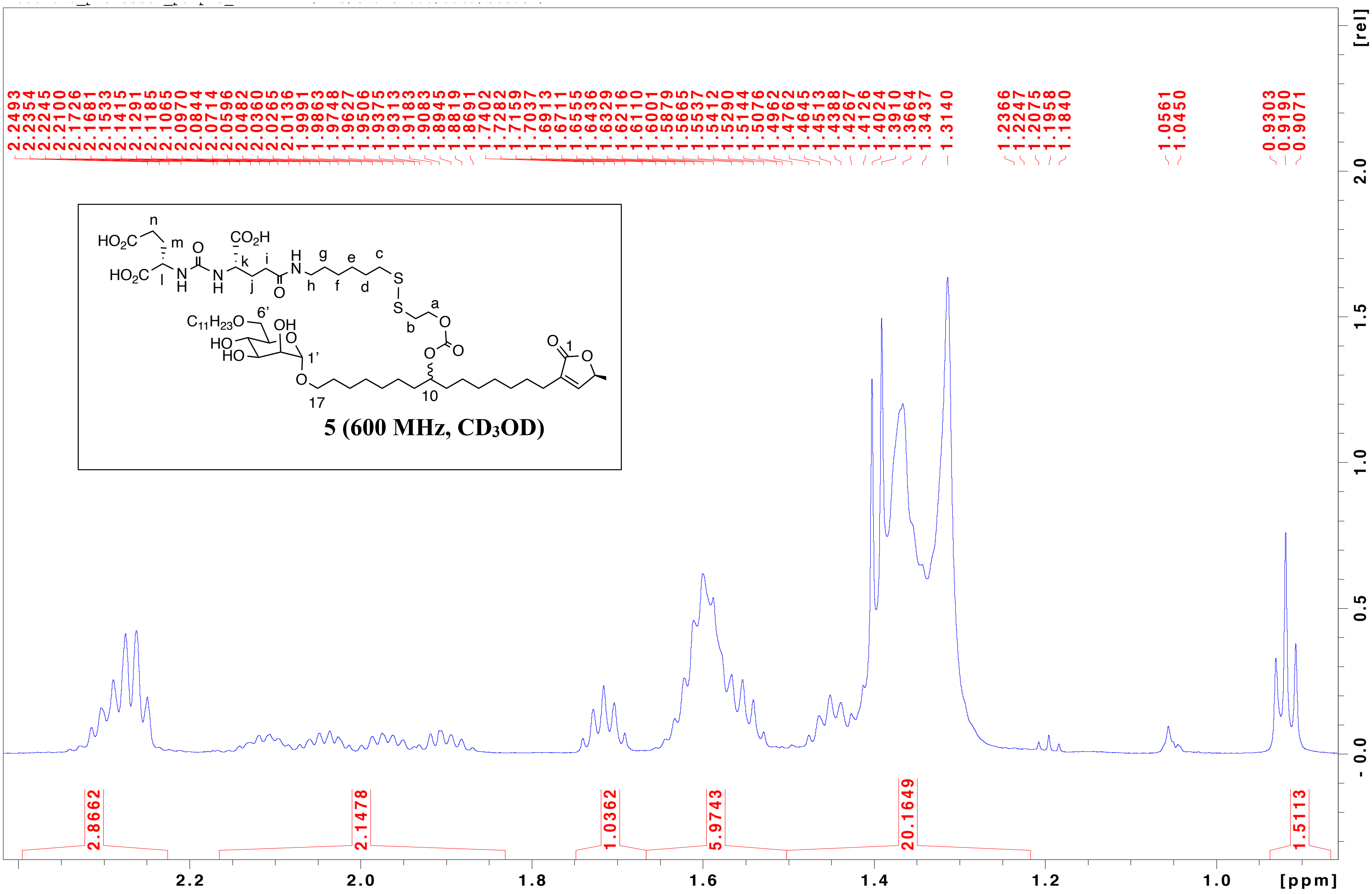
2.2493 2.2354 2.2245 2.2100 2.1726 2.1681 2.1533 2.1415 2.1291 2.1185 2.1065 2.0970 2.0844 2.0714 2.0596 2.0482 2.0360 2.0265 2.0136 1.9991 1.9863 1.9748 1.9627 1.9506 1.9375 1.9313 1.9183 1.9083 1.8945 1.8819 1.8691 1.7402 1.7282 1.7159 1.7037 1.6913 1.6711 1.6555 1.6436 1.6329 1.6216 1.6110 1.6001 1.5879 1.5665 1.5537 1.5412 1.5290 1.5144 1.5076 1.4962 1.4762 1.4645 1.4513 1.4388 1.4267 1.4126 1.4024 1.3910 1.3664 1.3437 1.3140 1.2366 1.2247 1.2075 1.1958 1.1840 1.0561 1.0450 0.9303 0.9190 0.9071
5 (600 MHz, CD3OD)
2.8662
2.1478
1.0362
5.9743
20.1649
1.5113
[rel]
[ppm]

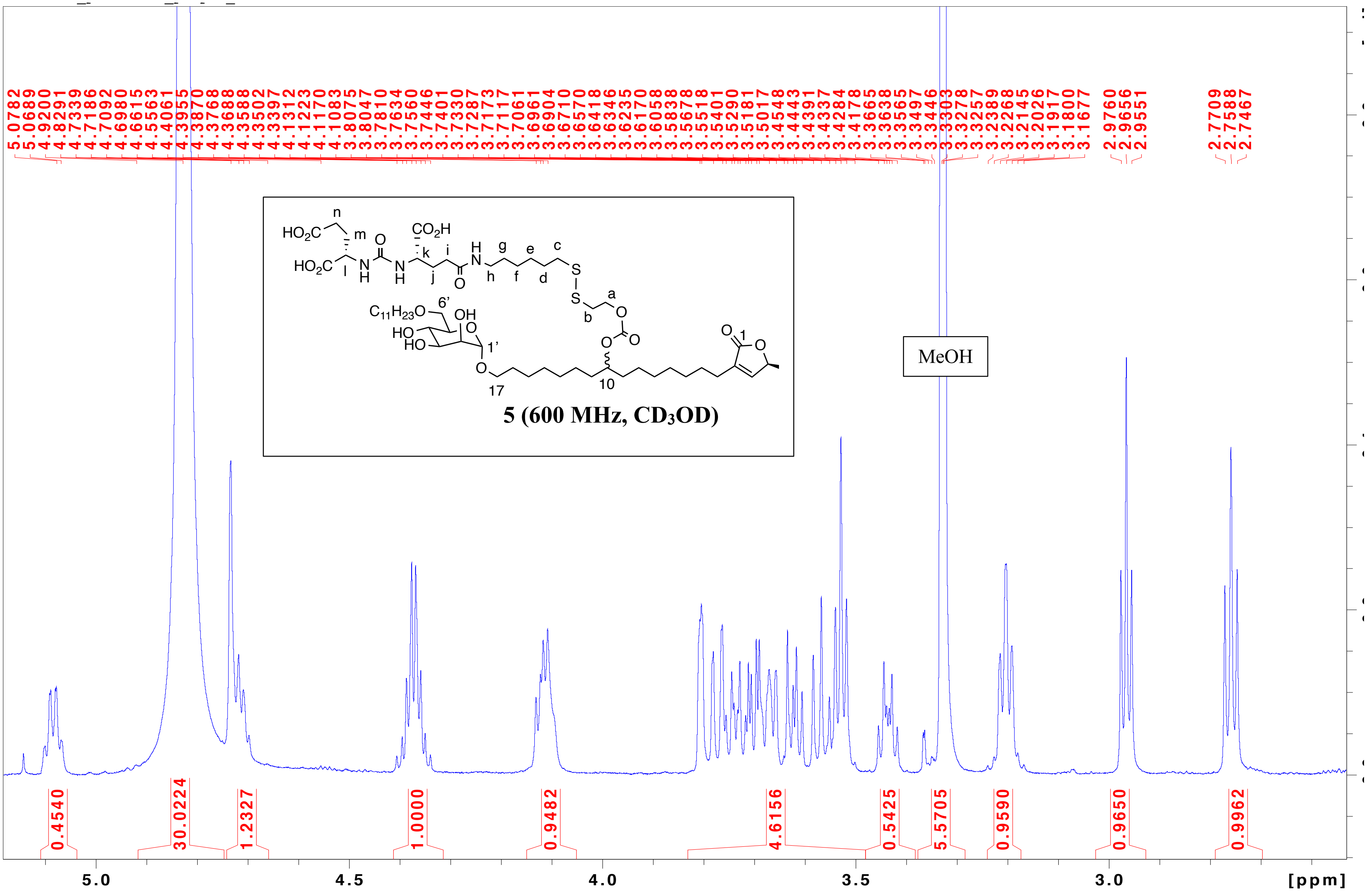
5 (600 MHz, CD3OD)
MeOH
[ppm]
5.0
4.5
4.0
3.5
3.0

5 (500 MHz, CD_3OD)

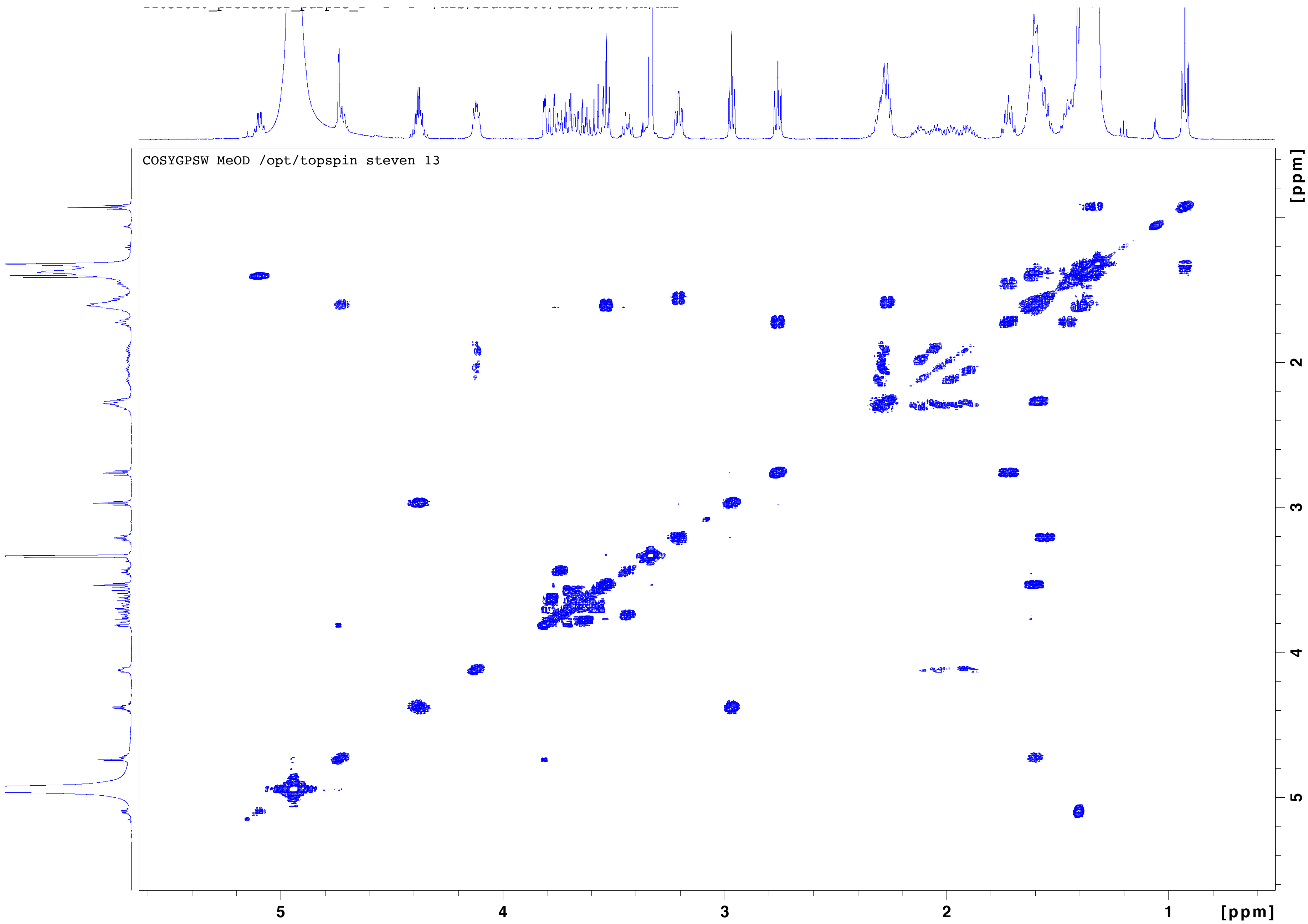

5 (500 MHz, CD3OD)

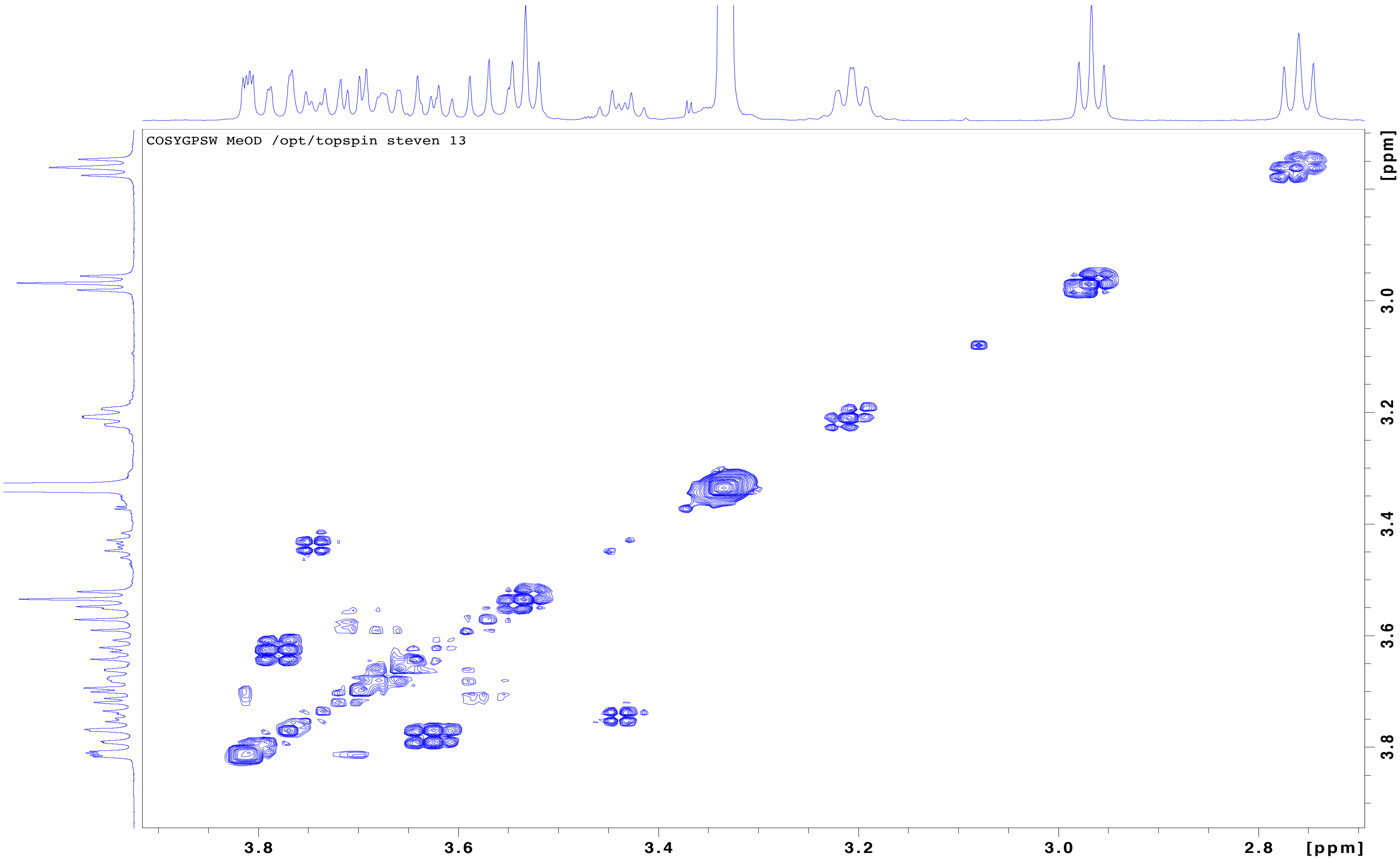

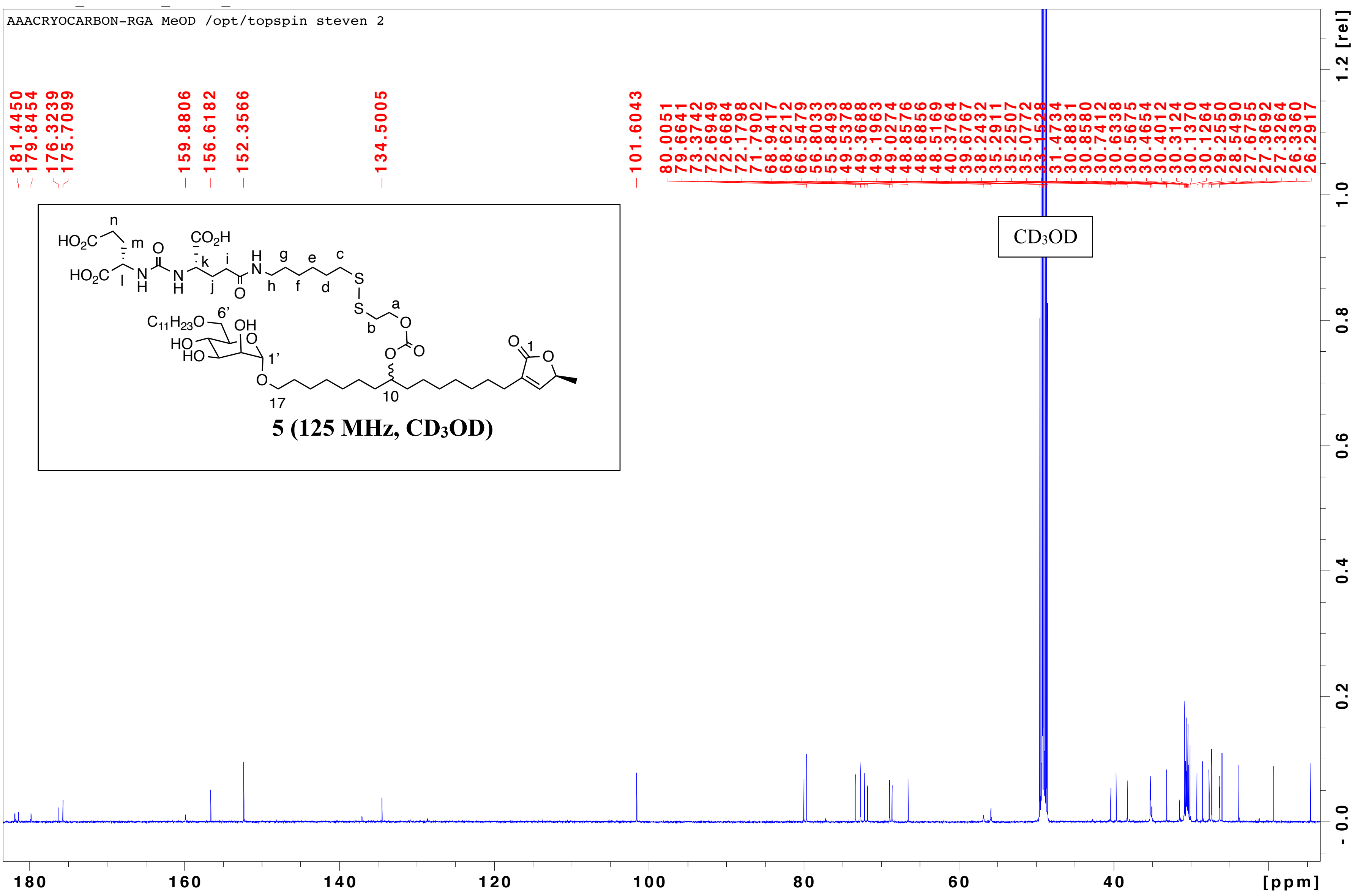
AAACRYOCARBON-RGA MeOD /opt/topspin steven 2
181.4450
179.8454
176.3239
175.7099
159.8806
156.6182
152.3566
134.5005
101.6043
80.0051
79.6641
73.3742
72.6949
72.6684
72.1798
71.7902
68.9412
68.6212
66.5479
56.8033
55.8493
49.5378
49.3688
49.1963
49.0274
48.8576
48.6856
48.5169
40.3764
39.6767
38.2432
35.2911
35.2507
35.0772
33.1528
31.4734
30.8831
30.8580
30.7412
30.6338
30.5675
30.4654
30.4012
30.3124
30.1370
30.1264
29.2550
28.5490
27.6755
27.3692
27.3264
26.3360
26.2917
CD3OD
5 (125 MHz, CD3OD)
[ppm]
[rel]

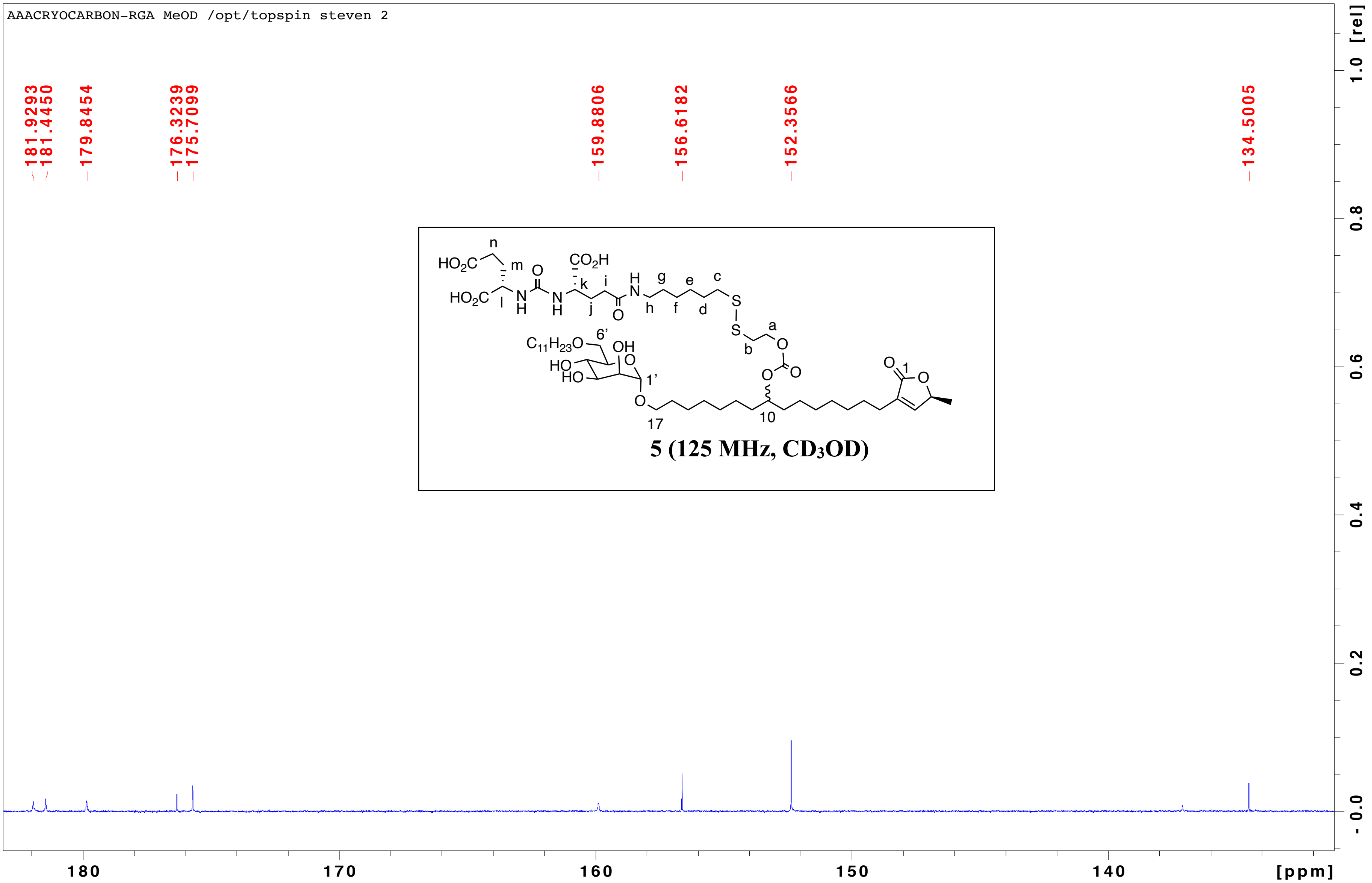
AAACRYOCARBON-RGA MeOD /opt/topspin steven 2
181.9293
181.4450
179.8454
176.3239
175.7099
159.8806
156.6182
152.3566
134.5005
5 (125 MHz, CD3OD)
180
170
160
150
140
[ppm]
[rel]

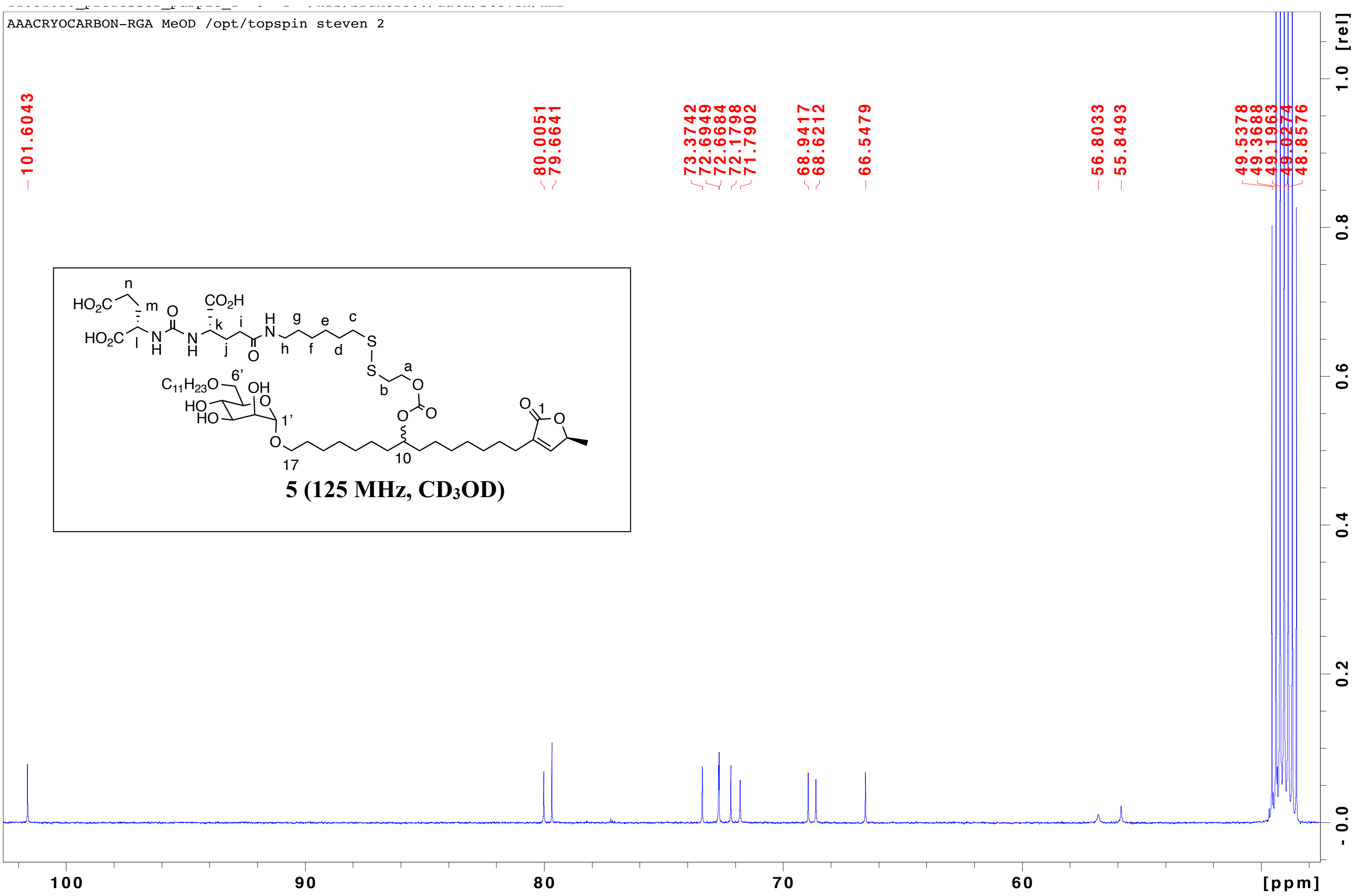
AAACRYOCARBON-RGA MeOD /opt/topspin steven 2
101.6043
80.0051
79.6641
73.3742
72.6949
72.6684
72.1798
71.7902
68.9417
68.6212
66.5479
56.8033
55.8493
49.5378
49.3688
49.1963
49.0274
48.8576
5 (125 MHz, CD3OD)
100
90
80
70
60
[ppm]
1.0 [rel]
0.8
0.6
0.4
0.2
- 0.0

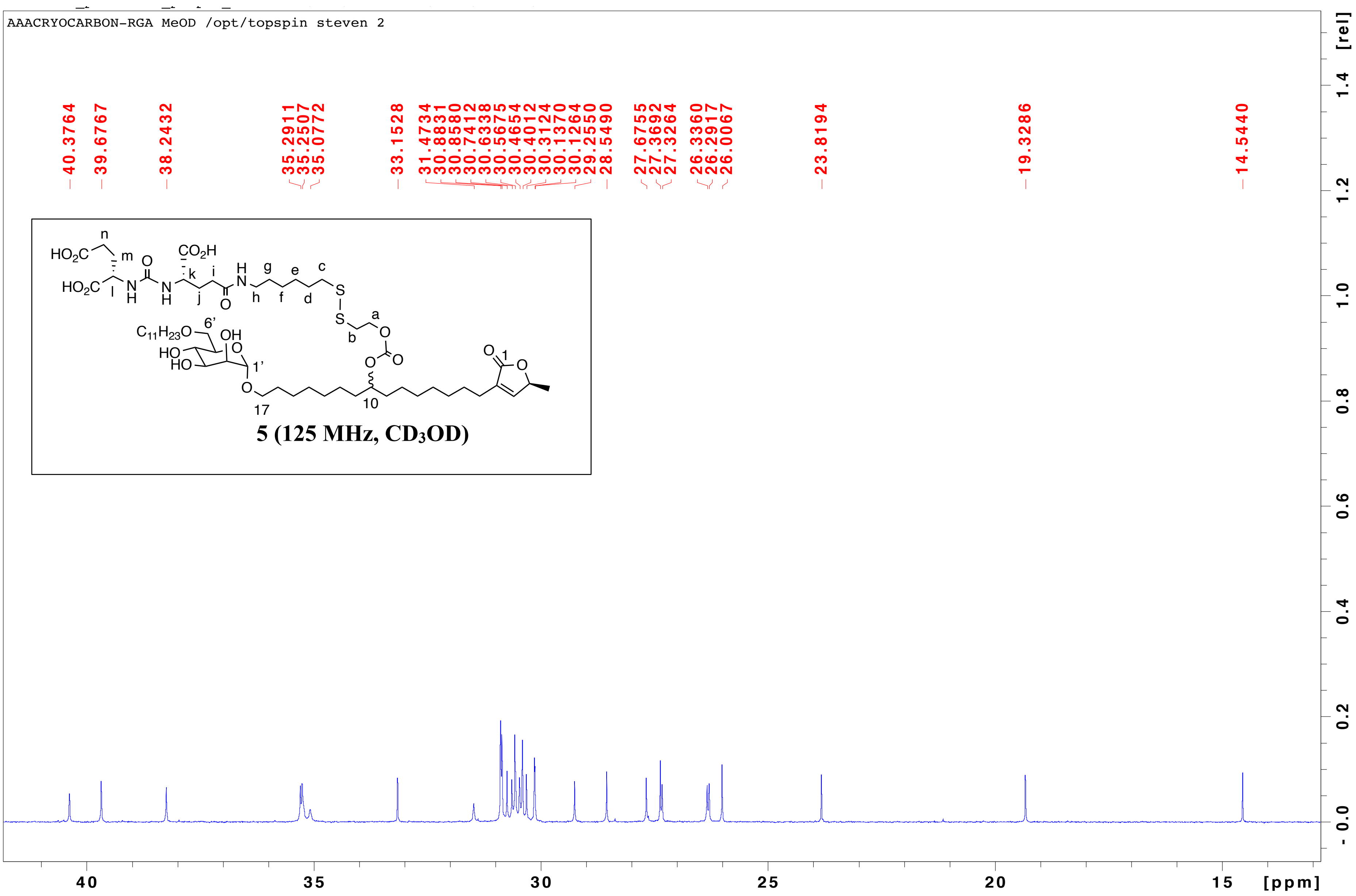

AAACRYOCARBON-RGA MeOD /opt/topspin steven 2
40.3764
39.6767
38.2432
35.2911
35.2507
35.0772
33.1528
31.4734
30.8831
30.8580
30.7412
30.6338
30.5675
30.4654
30.4012
30.3124
30.1370
30.1264
29.2550
28.5490
27.6755
27.3692
27.3264
26.3360
26.2917
26.0067
23.8194
19.3286
14.5440
5 (125 MHz, CD3OD)
[ppm]
[rel]

5 (500 MHz, CD3OD)

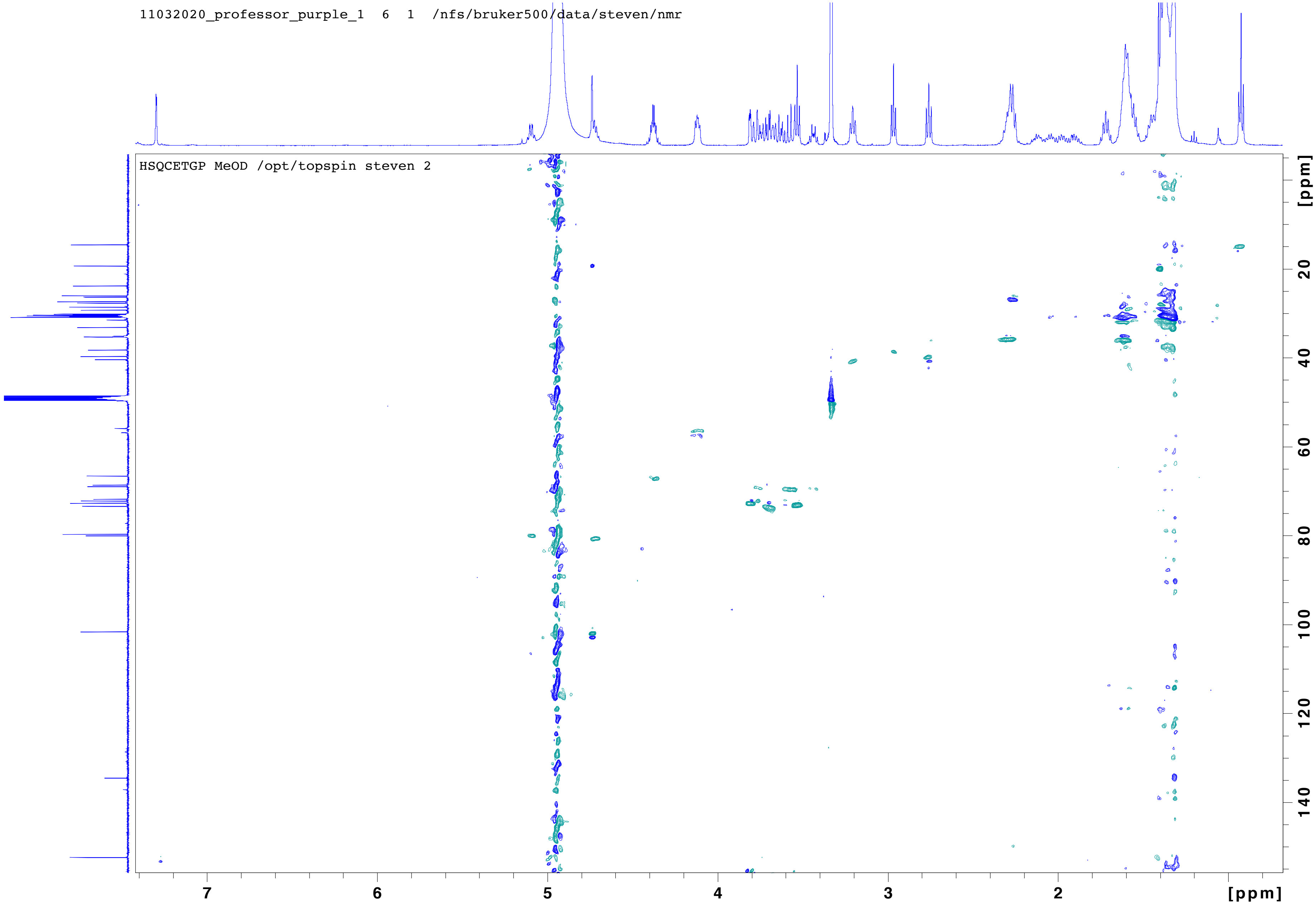

5 (500 MHz, CD_3OD)

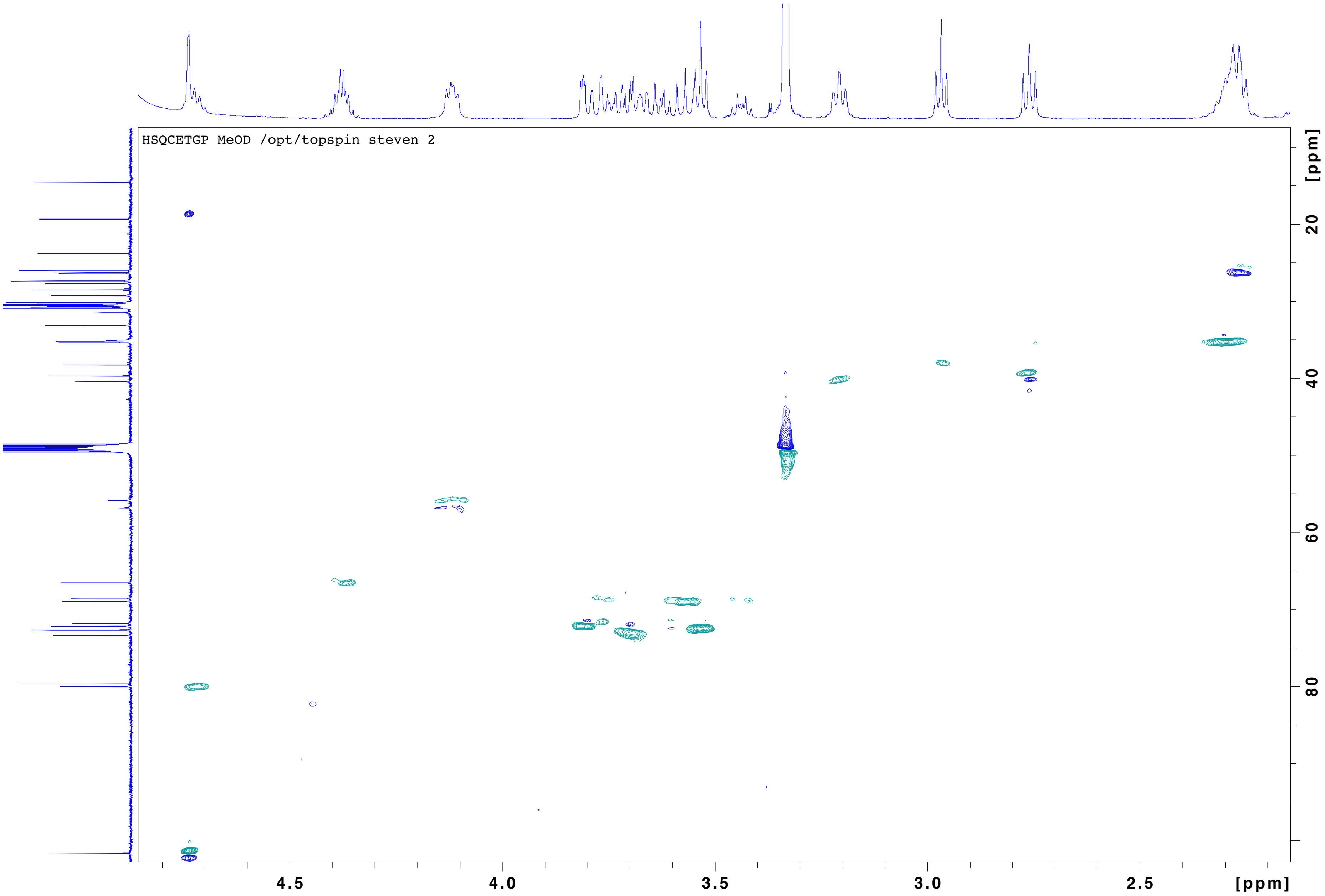

5 (500 MHz, CD3OD)

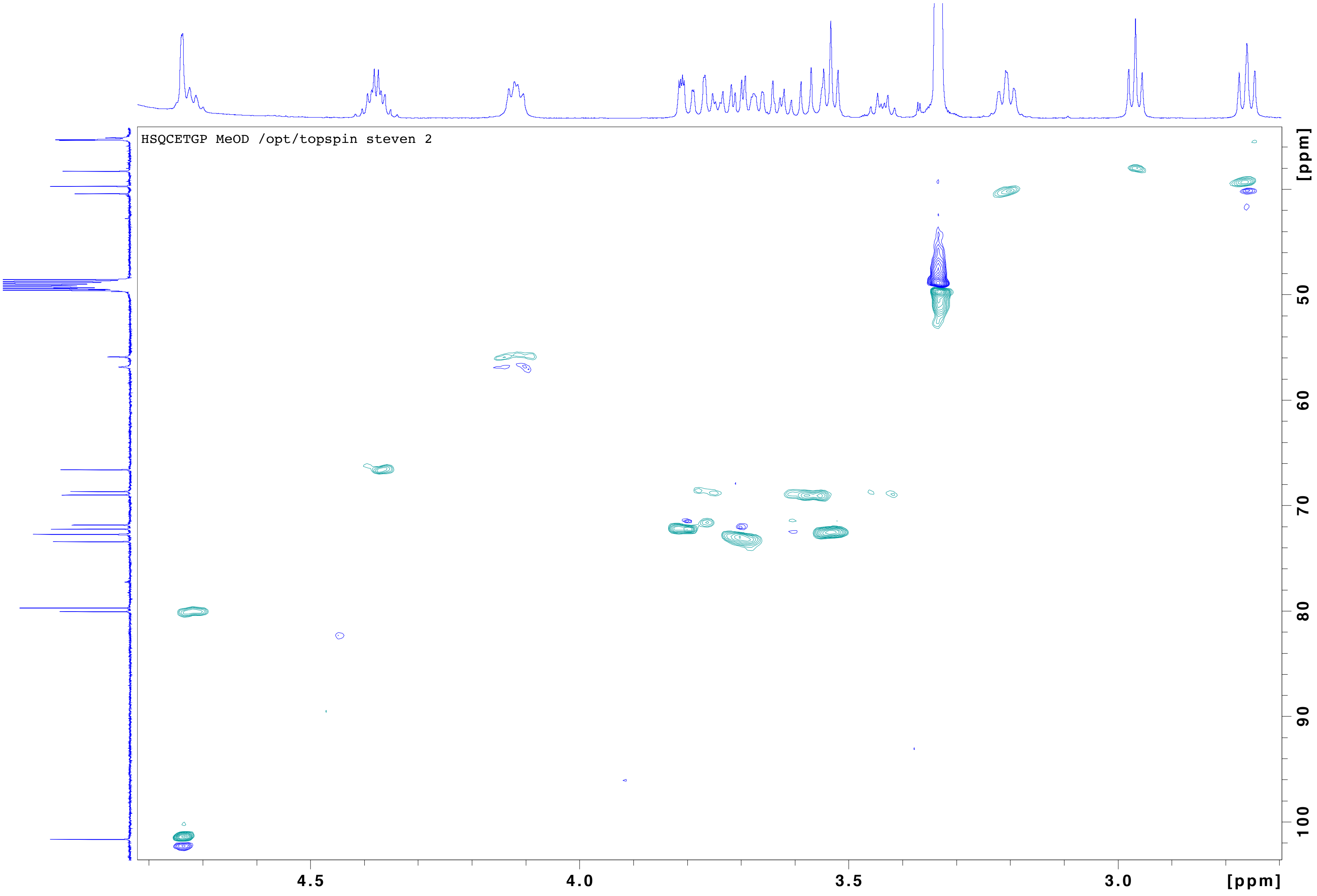

5 (500 MHz, CD_3OD)

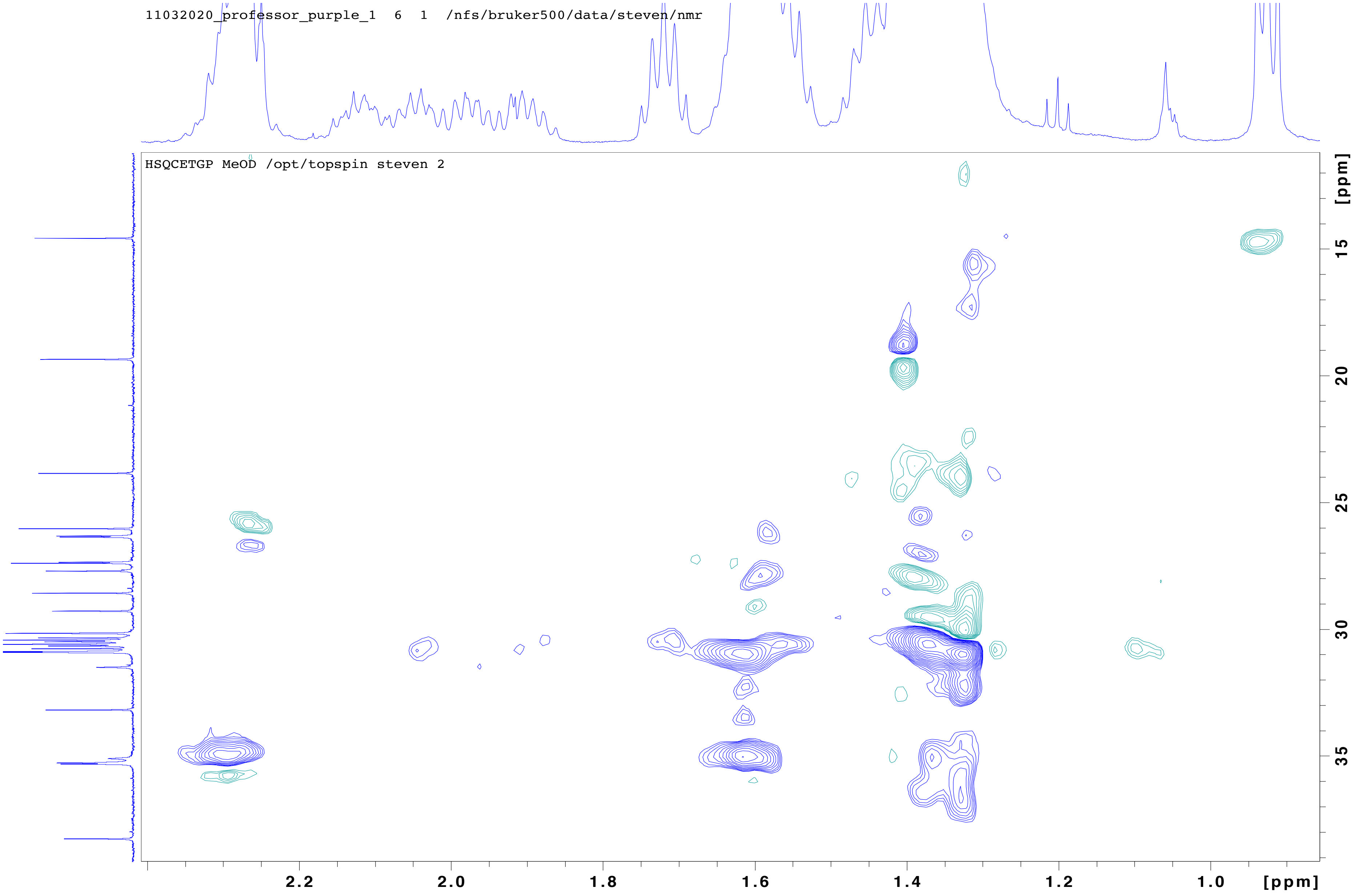

CYTOXICITY ASSAYS

Cell number optimization: Serial dilutions of cells in culture media were plated in triplicate from 100-100000 in a 96 well plate. Cells were incubated at 37C in a CO_2 incubator for 72hr. Activated solution of XTT reagent was made by adding 0.1ml of activation reagent to 5ml of XTT reagent. 50uL of activated XTT reagent was added per well. The plate was incubated for 2hr and absorbance from wells was measured at 475nM. Background was measured at 660nM. Background subtraction was made, and absorbance was plotted against cell number. Optimum cell number was determined based on the linear range of XTT standard curve.

IC50 determination: Serial dilutions of compounds to be tested were made in culture media from 1nM-1mM concentration. Determined cell number from the standard graph were plated in 96 well plate in triplicate per each dilution of the compounds. The cells were allowed to attach overnight by incubation at 37 ^{o}C in a CO_2 incubator. The media from the well was removed and media with serial dilutions of the compounds was added to the cells. The cells were incubated with the compound for 48h at 37 ^{o}C in CO_2 incubator. Activated XTT reagent was added to the wells and incubated for 2h. Absorbance from the wells was measured at 475nM and background at 660nM. Background subtraction was made, and all the data was plotted in prism and IC50 values were determined.

TEST COMPOUNDS

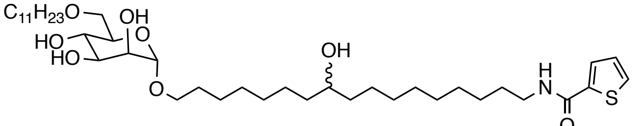

2

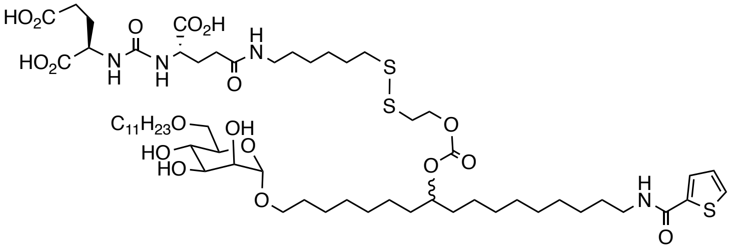

3

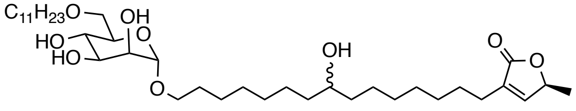

4

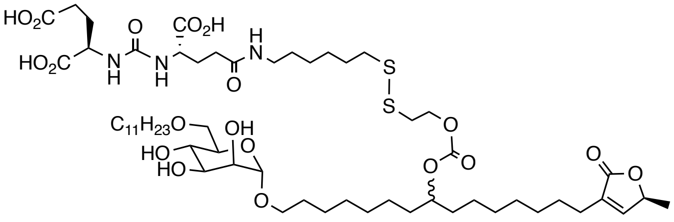

5

Doxorubicin (Adriamycin)[1]

[1] O. Tacar, P. Sriamornsak, C.R. Dass, Doxorubicin: an update on anticancer molecular action, toxicity and novel drug delivery systems. *J. Pharm. Pharmacol*. **2013**, *65* 157–170.

LNCaP

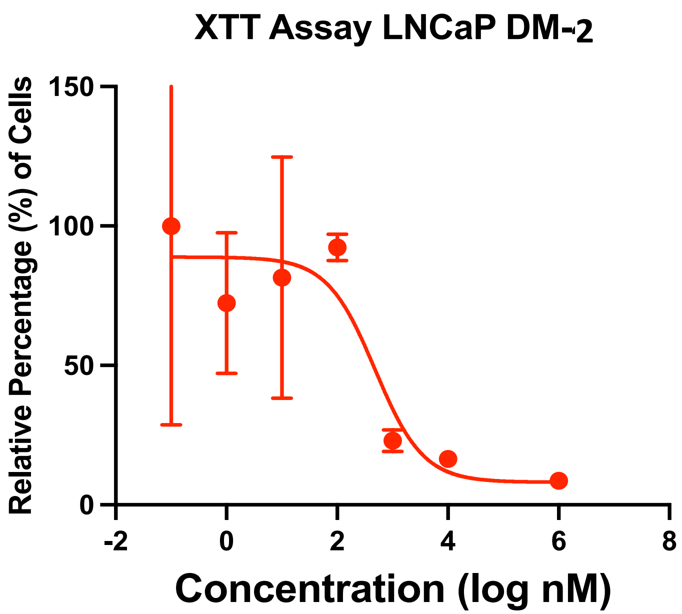

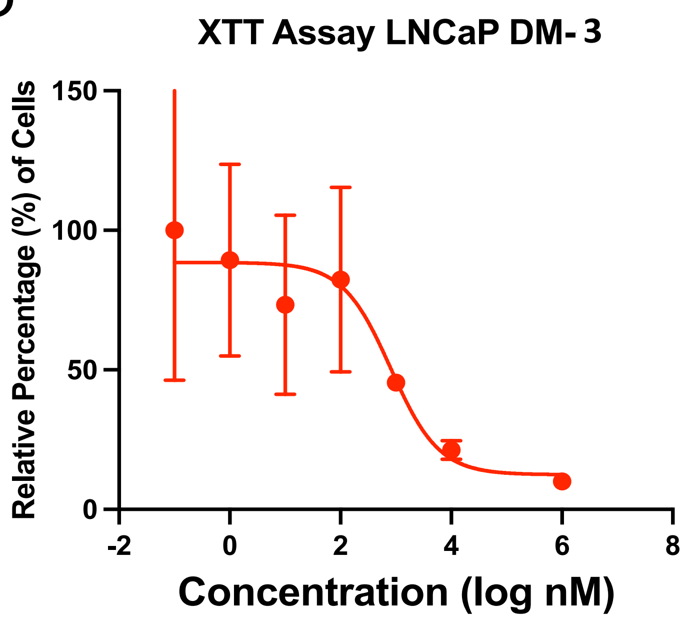

Compound Name	2
IC50 (nM)	483.9
95% CI (nM)	71.08 to 36666
R squared (goodness to fit)	0.5783

Compound Name	3
IC50 (nM)	810.5
95% CI (nM)	72.08 to 7762
R squared (goodness to fit)	0.6078

PC3

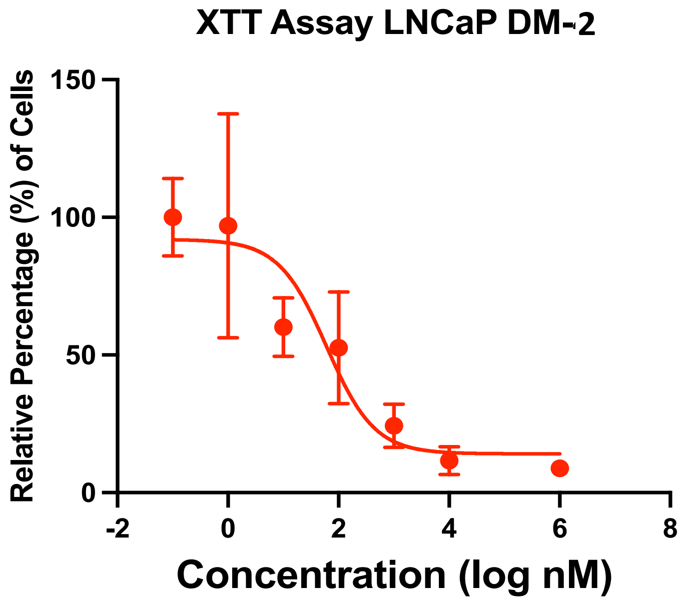

Compound Name	2
IC50 (nM)	60.81
95% CI (nM)	3.761 to 469.7
R squared (goodness to fit)	0.7724

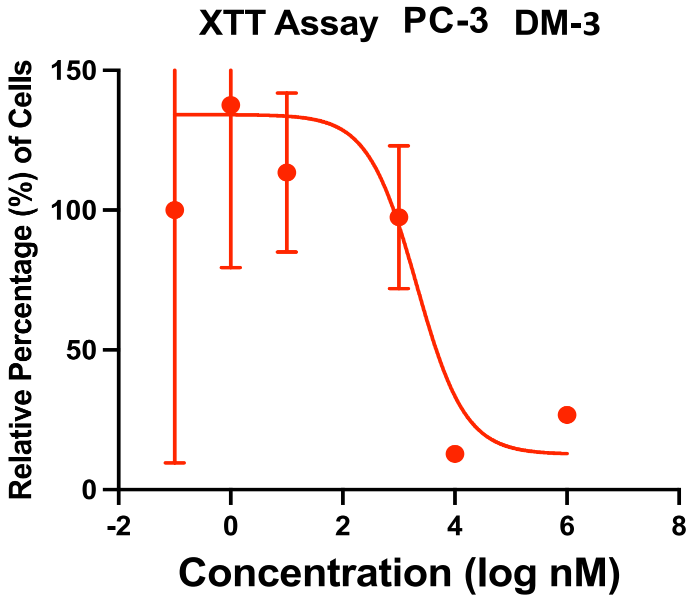

Compound Name	3
IC50 (nM)	2074
95% CI (nM)	187.7 to 23468
R squared (goodness to fit)	0.4165

LNCaP

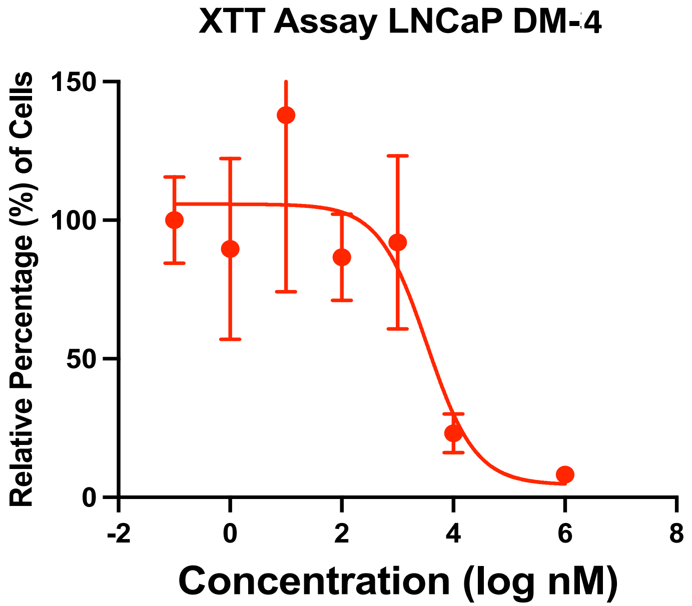

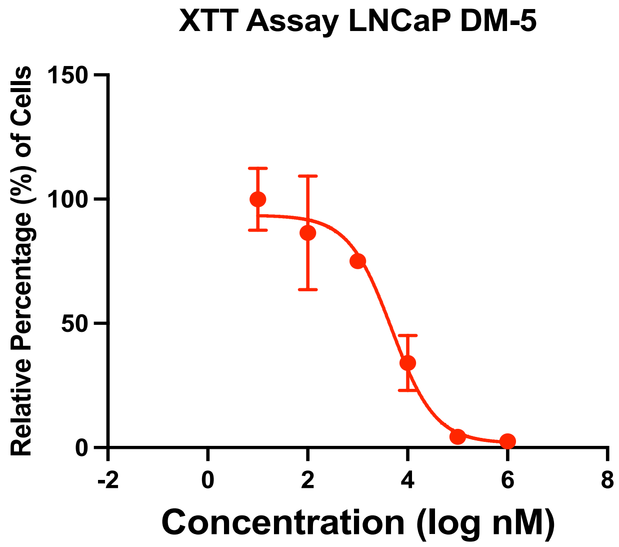

Compound Name	4
IC50 (nM)	3369
95% CI (nM)	501.1 to 17077
R squared (goodness to fit)	0.6316

Compound Name	5
IC50 (nM)	4809
95% CI (nM)	1703 to 12182
R squared (goodness to fit)	0.9477

PC3

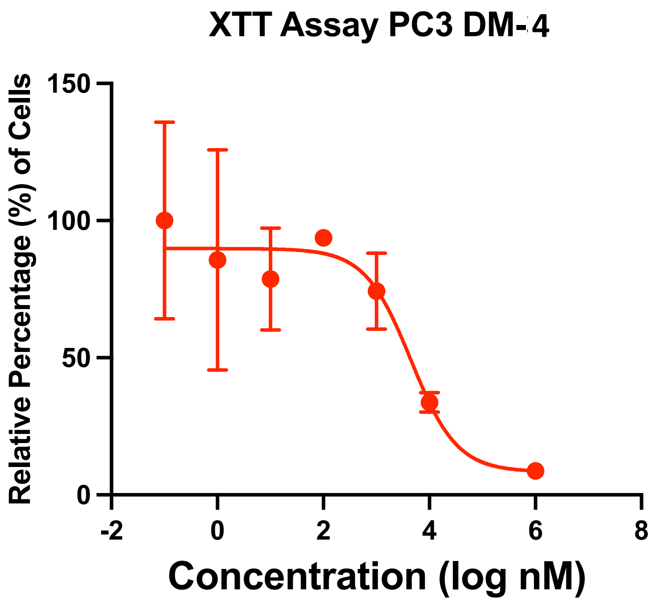

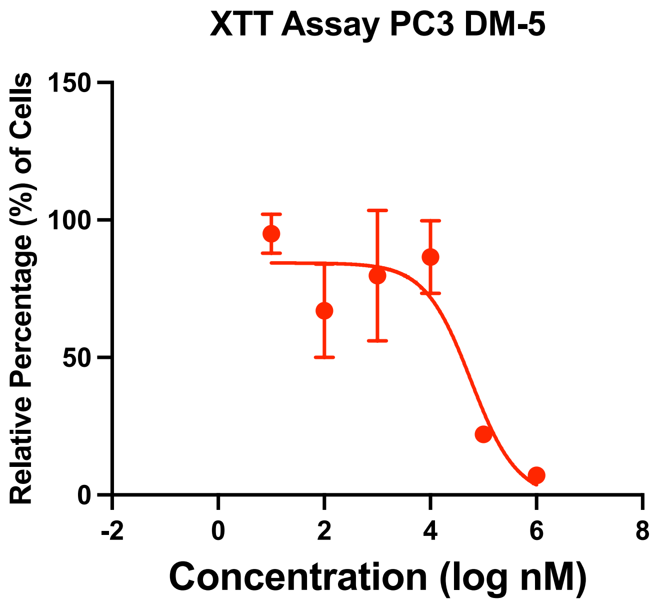

Compound Name	4
IC50 (nM)	4491
95% CI (nM)	892.0 to 18624
R squared (goodness to fit)	0.7175

Compound Name	5
IC50 (nM)	58040
95% CI (nM)	13523 to 261332
R squared (goodness to fit)	0.8239

LNCaP

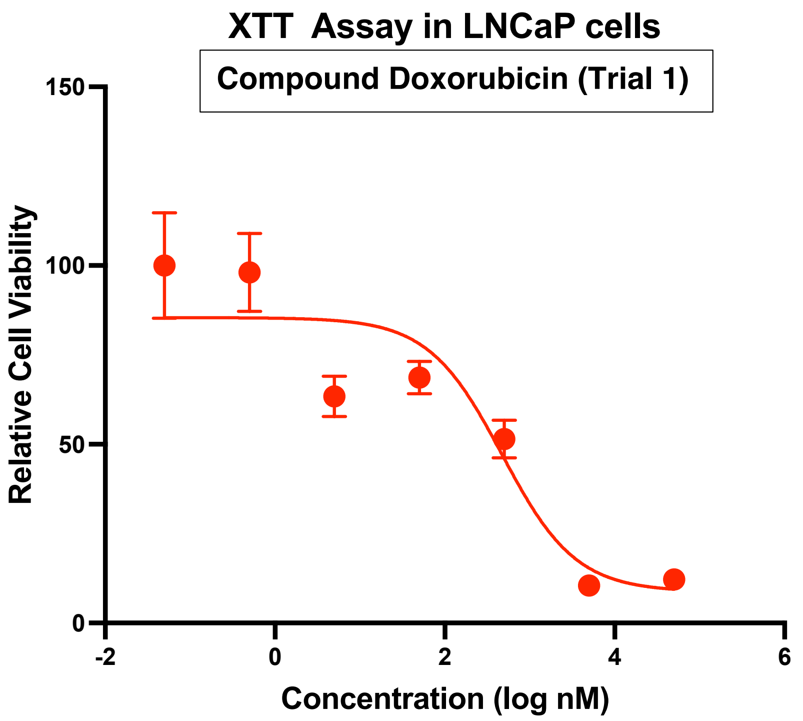

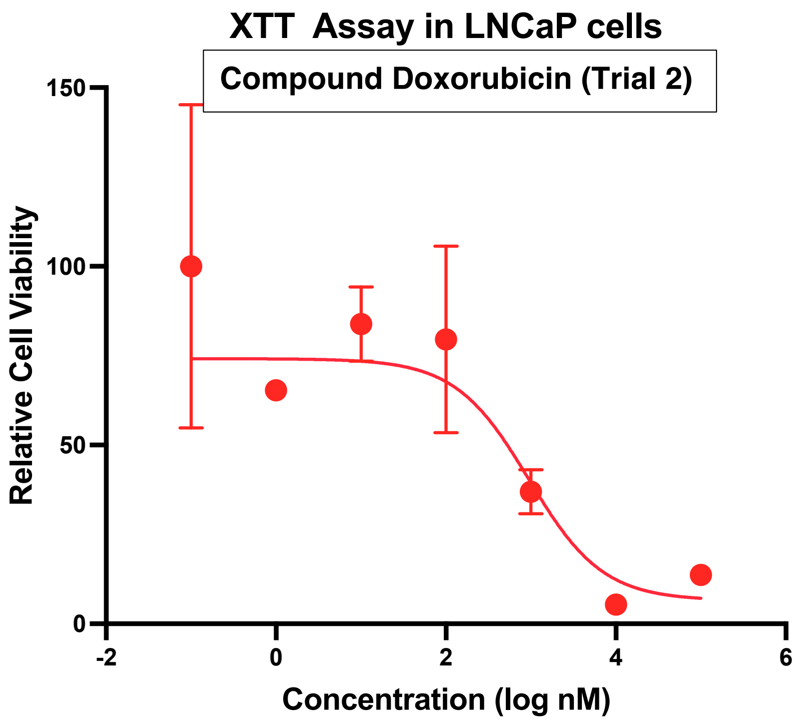

Compound Name	Doxorubicin (Trial 1)
IC50 (nM)	472.0
95% CI (nM)	66.52 to 1847
R squared (goodness to fit)	0.7928

Compound Name	Doxorubicin (Trial 2)
IC50 (nM)	947.7
95% CI (nM)	175.0 to 5490
R squared (goodness to fit)	0.6431

PC-3

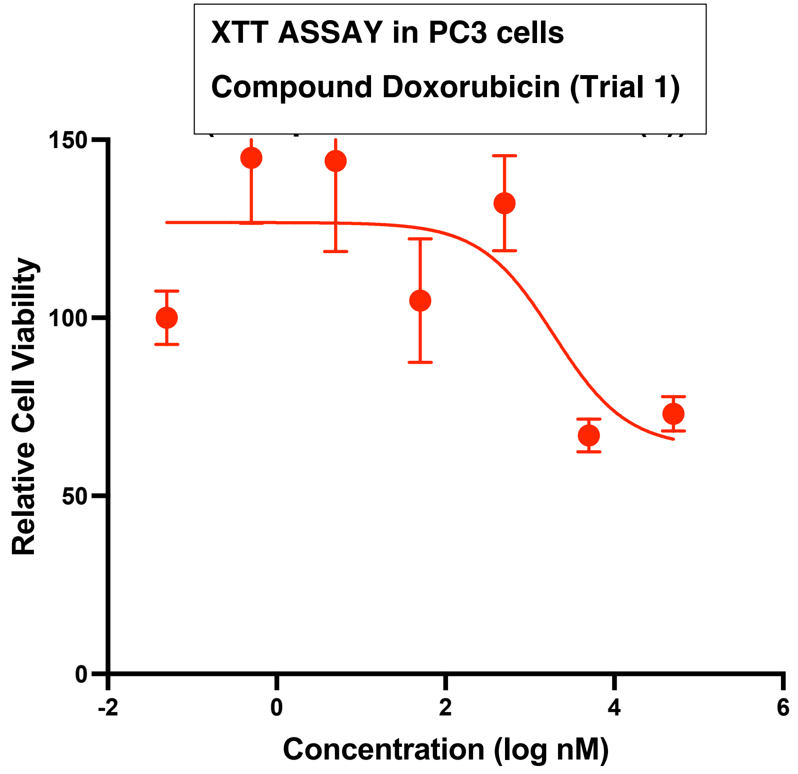

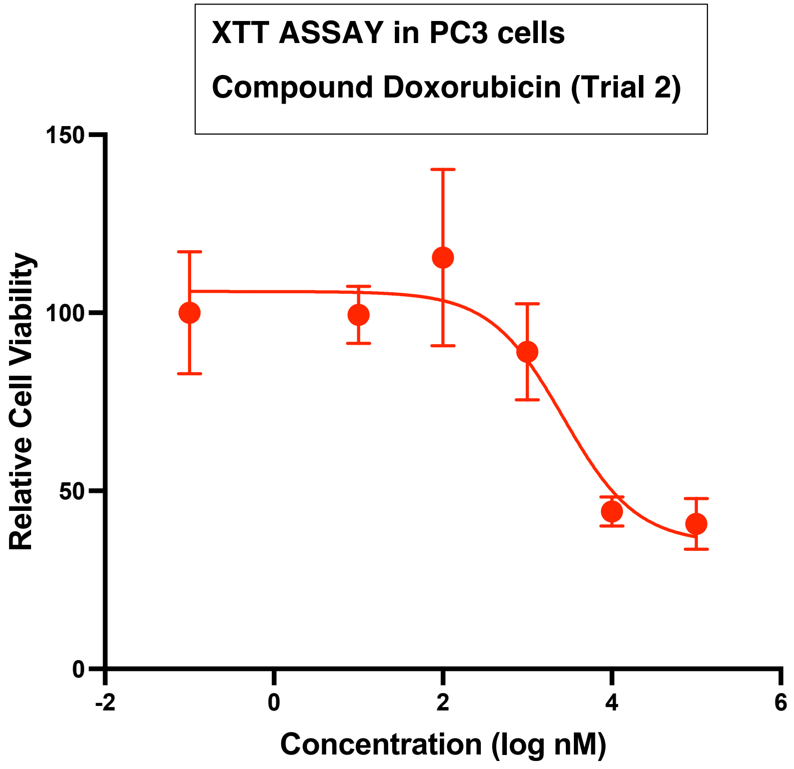

Compound Name	Doxorubicin (Trial 1)
IC50 (nM)	1919
95% CI (nM)	65.46 to 29442
R squared (goodness to fit)	0.4127

Compound Name	Doxorubicin (Trial 2)
IC50 (nM)	2656
95% CI (nM)	413.4 to 18085
R squared (goodness to fit)	0.6312
